# Reverse barrier layer and interfacial M–O bond reinforcement in an S-scheme high-entropy oxide heterojunction for efficient photo-assisted lithium–oxygen batteries

**DOI:** 10.1039/d6sc05480c

**Published:** 2026-08-04

**Authors:** Bai Zheng, Qinghua Sun, Rourou Yi, Huiying Zhou, Wenju Xie, Fuli Xie, Jie Zhao, Yanhe Xiao, Shuijin Lei, Baochang Cheng

**Affiliations:** a School of Physics and Materials Science, Nanchang University Jiangxi 330031 P. R. China jzhao@ncu.edu.cn bcheng@vip.sina.com; b School of Electronic Information and Physics, Central South University of Forestry and Technology Hunan 410004 P. R. China T20070600@csuft.edu.cn; c College of Ecology and Resources Engineering, Fujian Provincial Key Laboratory of Eco-Industrial Green Technology, Wuyi University Fujian 354300 P. R. China

## Abstract

Lithium–oxygen batteries (LOBs), as a promising next-generation high-energy-density system, are fundamentally limited by sluggish oxygen reduction (ORR) and oxygen evolution (OER) kinetics, resulting in high overpotentials and poor cycling stability. Herein, a p-type high-entropy spinel oxide (FeCoNiMnZnCrO_*x*_, HEO) was integrated with n-type photoactive TiO_2_ to construct an S-scheme heterojunction cathode (HEO@TiO_2_). At the heterointerface, Ti–O–M bonds (M = Fe, Co, Ni, Mn, Zn, Cr) enhance electron transport through Ti-induced electronic modulation and strengthen orbital hybridization between transition-metal 3d and O 2p, optimizing the adsorption energies of key intermediates (O_2_^−^ and LiO_2_) and lowering the energy barriers of the ORR/OER. Concurrently, a reverse barrier layer promotes reversible Li_2_O_2_ formation and decomposition. Additionally, a photoelectric effect accelerates charge transport and electrode kinetics, while electron enrichment on HEO amplifies the high-entropy cocktail effect. Benefiting from the synergy of high-entropy mixing, interfacial M–O bond reinforcement, and light-assisted S-scheme heterojunction, HEO@TiO_2_ delivers an ultrahigh specific capacity of 29 545 mA h g^−1^ and stable cycling over 867 cycles under dark conditions. Under illumination, an ultralow overpotential of 0.15 V is achieved, the charging rate increases by 22.4%, and Li_2_CO_3_ byproduct formation is effectively suppressed. This work presents a novel interface-coupling strategy for high-performance LOB cathodes and dual-function photo-assisted electrocatalysis.

## Abstract 2

An S-scheme heterojunction of photoresponsive high-entropy FeCoNiMnZnCrO_*x*_ with TiO_2_ enables charge transfer and kinetics in Li–O_2_ batteries, with entropy, covalency, and heterointerface giving dark performance and light boosting photocatalysis.

## Graphical abstract

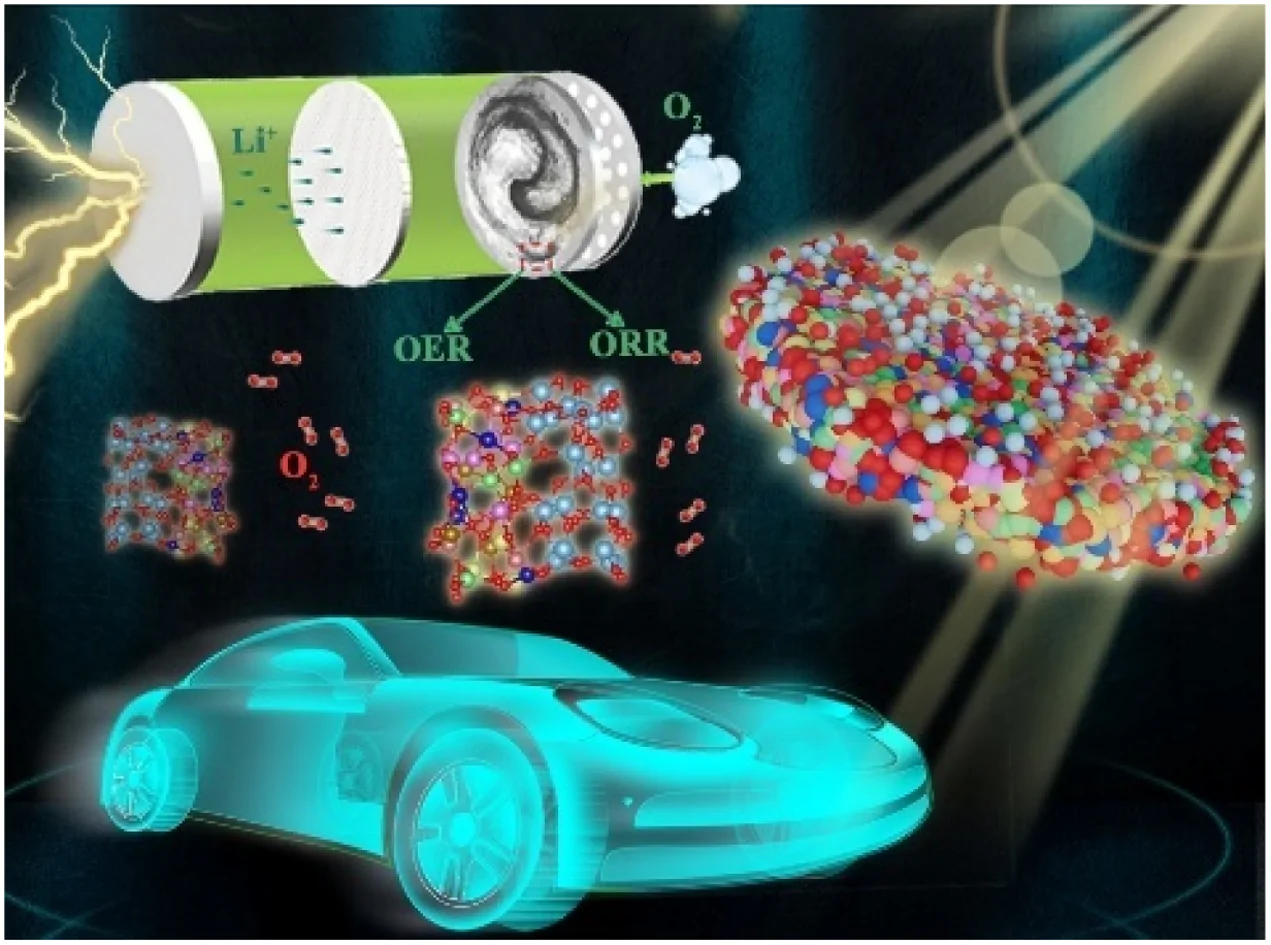

## 1 Introduction

The escalating global energy crisis and intensifying environmental imperatives demand advanced energy storage systems for a sustainable, low-carbon economy. While lithium-ion batteries (LIBs) dominate the market, their limited energy density increasingly falls short for high-performance applications.^1–4^ Lithium–oxygen batteries (LOBs) offer a compelling alternative, boasting a theoretical energy density of ∼3500 Wh kg^−1^*via* the reversible reaction: 2Li^+^ + O_2_ + 2e^−^ ⇌ Li_2_O_2_ (*E*^0^ = 2.96 V *vs.* Li/Li^+^).^5–10^ However, practical LOB deployment faces significant challenges, primarily sluggish oxygen reduction (ORR) and evolution (OER) kinetics. Initial strategies employed high-surface-area porous carbon cathodes to accommodate Li_2_O_2_ and enhance reversibility. Unfortunately, carbon's susceptibility to oxidation at high potentials triggers parasitic reactions, leading to rapid capacity fade.^11,12^ Noble metal catalysts (*e.g.*, Ru and Ir) were subsequently introduced to accelerate ORR/OER kinetics, yet their high cost hinders large-scale adoption.^13,14^ This limitation shifted focus toward abundant transition metal-based compounds (*e.g.*, Fe, Co, and Ni), where catalytic performance, governed by the d-band electronic structure, can be optimized through coordination environment modulation.^15–19^ Despite progress, persistent issues like metal dissolution and detrimental electrolyte decomposition continue to compromise performance.^18,20,21^ Recent strategies, including single-atom catalysts,^22,23^ metal oxides,^24,25^ and sulfides,^26–28^ have diversified cathode design paradigms. Yet, achieving commercially viable LOBs necessitates fundamental breakthroughs in reaction mechanism elucidation, interfacial engineering, and integrated system optimization—specifically targeting synergistic enhancement of catalytic activity, structural stability, and oxygen species transport.^29^

High-entropy oxides (HEOs), an emerging class of multi-cationic materials, present unique advantages for electrocatalysis.^30^ Comprising five or more near-equimolar metal elements, HEOs leverage the high-entropy effect to stabilize single-phase solid solutions and suppress phase segregation.^31^ Their exceptional properties—including lattice distortion, sluggish diffusion, and the crucial cocktail effect—collectively optimize the electronic structure and reaction energetics.^32^ The cocktail effect, arising from synergistic multi-metal interactions, significantly enhances catalytic performance.^33^ To address the OER trade-off between lattice-oxygen activation and structural stability, Xu's team synthesized single-crystalline porous hollow high-entropy ZnVCrMoMn spinel oxides *via* a dual-sacrificial template; multi-component electronic interactions and high-valence cations strengthened M–O covalency, favouring a stable lattice-oxygen mechanism (LOM) pathway.^34^ Beyond the OER, Niu *et al.* designed spinel (CrMnFeCoNi)_3_O_4_ HEO oxide nanofibers by electrospinning and heat treatment, where abundant oxygen vacancies and redox-active octahedral sites improved conductivity, charge transfer, and three-phase boundaries for efficient ORR/OER.^35^ Tian *et al.* found that optimized Co valence in (Co_0.2_Mn_0.2_Ni_0.2_Fe_0.2_Cr_0.2_)_3_O_4_ promotes LiO_2_ adsorption and kinetics in LOBs.^36^ Beyond entropy effects, recent work underscores interfacial engineering in HEO as decisive—*e.g.*, Zhang's team combined single-atom Cu with high-entropy oxides, using interfacial electric fields and electronic modulation to overcome sluggish Li_2_S oxidation in lithium–sulphur batteries.^37^ Similarly, constructing allotropic dual-phase heterointerfaces in HEOs enables strong electronic coupling and balanced adsorption of reaction intermediates, thereby significantly reducing reaction energy barriers for OER/HER processes.^38^ Similarly, precise heterostructure integration in high-entropy systems induces interfacial charge redistribution, optimizes the d-band centre of active sites, and strengthens M–O covalency, ultimately lowering kinetic barriers and improving catalytic stability.^39^ This highlights that catalytic activity is fundamentally governed by the interaction between catalyst electronic states and oxygen intermediates in lithium–oxygen batteries (LOBs).^40^

Concurrently, TiO_2_, an established photocatalytic material, possesses a suitable band gap and exceptional photostability, thereby effectively suppressing photogenerated electron–hole recombination.^41^ Crucially, its anatase phase demonstrates excellent corrosion resistance and maintains structural integrity under both acidic and alkaline conditions during illumination.^42^ These attributes make TiO_2_ a promising candidate for heterojunction-based photocathodes in LOBs, where it aims to enhance electrochemical performance by accelerating reaction kinetics.^43–45^ Specifically, under illumination, the ORR proceeds at the photocathode: adsorbed O_2_ molecules are reduced by photogenerated electrons, subsequently combining with Li^+^ ions to form Li_2_O_2_. Concurrently, photogenerated holes in the valence band are balanced by electrons from the external circuit. During charging, these holes facilitate Li_2_O_2_ oxidation, resulting in O_2_ evolution.^46,47^ Theoretically, this light-assisted mechanism can significantly accelerate both Li_2_O_2_ formation and decomposition, thus lowering overpotentials and improving energy efficiency. However, persistent challenges include insufficient light harvesting and inefficient separation of photogenerated charge carriers, representing critical limitations.

To overcome these issues, heterostructures incorporating built-in electric fields have been developed to promote spatial separation and migration of photogenerated carriers. Herein, the nature of the heterojunction interface is paramount, as it dictates charge transport behaviour. If a blocking-type (Schottky-like) interface forms, majority carrier depletion at the junction creates a potential barrier, restricting charge flow unidirectionally. Conversely, when both semiconductors form ohmic-type contacts, majority carriers accumulate at the interface, enabling bidirectional charge transport. Given that efficient battery operation necessitates bidirectional charge conduction, constructing such ohmic-type interfaces not only facilitates unimpeded carrier transport across the heterojunction but also further enhances the separation of electron–hole pairs under illumination.

Building on these principles, we designed an S-scheme heterojunction cathode (HEO@TiO_2_) integrating p-type high-entropy spinel oxide (HEO) and n-type TiO_2_. This design establishes efficient electron transport channels *via* interfacial Ti–O–M (M = Fe, Co, Ni, Mn, Zn, Cr) bonds. Ti modulation weakens Ti–O bonds while enhancing the character of adjacent M–O bonds, promoting strong hybridization between transition metal 3d and oxygen 2p orbitals. This synergy optimizes adsorption energies for key intermediates (*e.g.*, O_2_ and LiO_2_) and significantly reduces ORR/OER energy barriers. Critically, the HEO/TiO_2_ p–n junction generates a directional built-in electric field, driving efficient charge separation and enabling high-performance LOBs. The S-scheme structure further harnesses light to amplify redox kinetics: electron accumulation on the HEO surface intensifies the high-entropy cocktail effect, boosting ORR activity and elevating the discharge plateau. As an LOB cathode, HEO@TiO_2_ delivers a high specific capacity of 29 545 mA h g^−1^ and stable cycling over 867 cycles (dark). Under illumination, the discharge potential increases while the charge potential decreases, reducing the overpotential to 0.15 V. Charging rates increase by ∼22.4% in constant-voltage mode, demonstrating exceptional photoelectrochemical synergy. This study achieves targeted interfacial modification of HEO *via* TiO_2_, constructing an S-scheme heterojunction with a defined built-in field. It harnesses the multiscale synergy of the high-entropy cocktail effect, interfacial M–O bond reinforcement, and directional S-scheme charge transfer, providing novel design principles for high-performance bifunctional LOB cathodes and efficient light-assisted systems.

## 2 Results and discussion

### 2.1. Synthesis and characterization of HEO@TiO_2_ materials

In this work, TiO_2_-modified HEO-based heterojunction materials (HEO@TiO_2_) were rationally designed and synthesized. As schematically illustrated in Fig. 1a, equimolar metal nitrates (Fe, Co, Ni, Mn, Zn, and Cr) were dissolved in deionized water, followed by glycine addition as a combustion fuel. Vigorous stirring yielded a homogeneous gel-like precursor, which was subsequently combusted in air to form the 6-component HEO spinel (FeCoNiMnZnCrO_*x*_). The HEO powder was then hydroxylated, dispersed in ethanol, and subjected to hydrothermal treatment with tetrabutyl titanate to grow TiO_2_ nanoparticles on its surface. Centrifugation, washing, and drying yielded the final HEO@TiO_2_ composite.

**Fig. 1:**
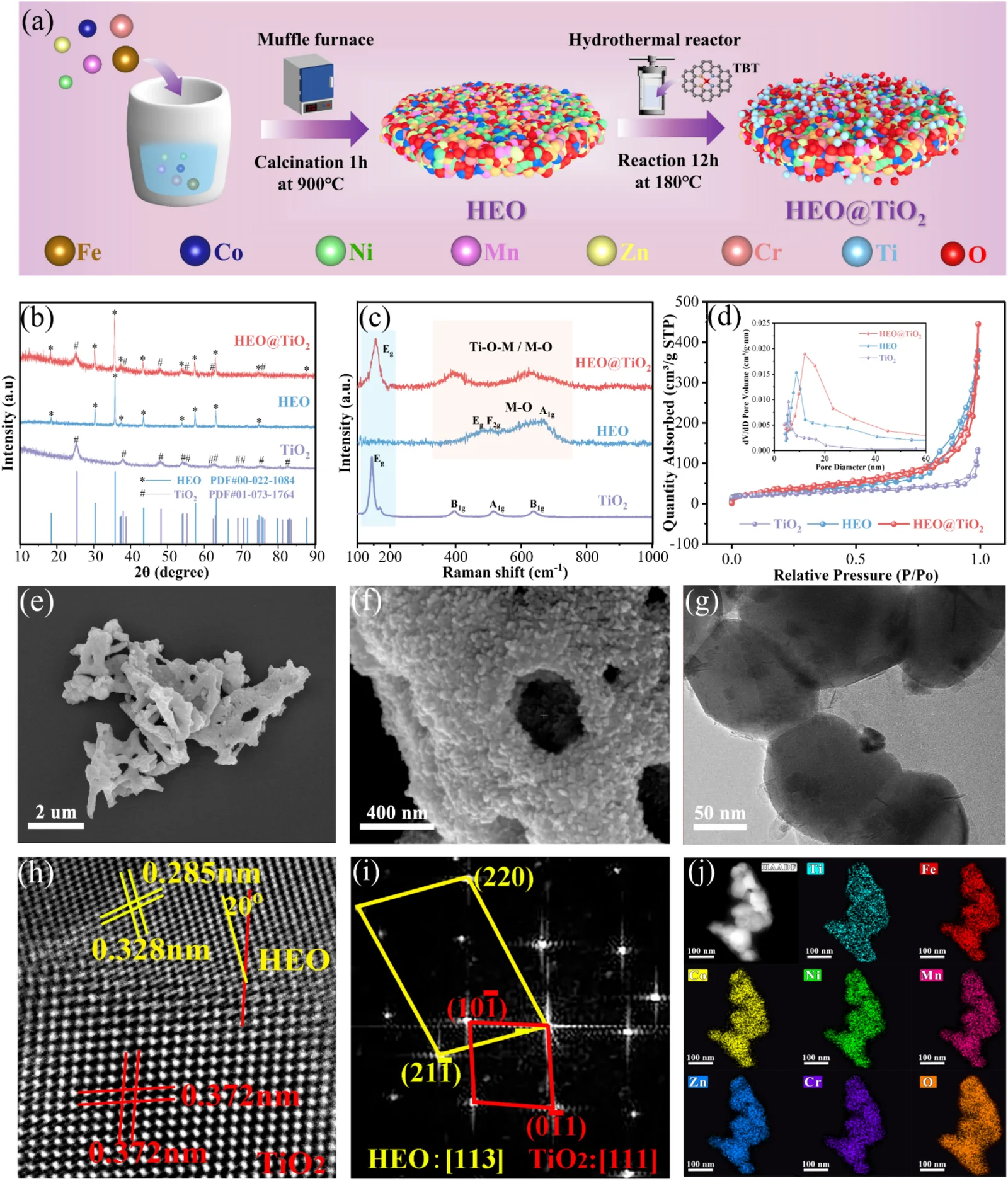
Morphological and structural characterization of the as-synthesized products. (a) Schematic illustration of the synthesis process for HEO@TiO_2_. (b) XRD patterns of TiO_2_, HEO, and HEO@TiO_2_. (c) Raman spectra of TiO_2_, HEO, and HEO@TiO_2_. (d) N_2_ adsorption–desorption isotherms and corresponding pore size distribution (inset). (e) and (f) SEM images of HEO@TiO_2_ and corresponding magnified view. (g) Bright-field TEM image of HEO@TiO_2_. (h) and (i) HRTEM image of the HEO@TiO_2_ heterointerface and corresponding FFT pattern. (j) HAADF-STEM image and elemental mapping profiles.

X-ray diffraction (XRD) analysis (Fig. 1b) confirmed the phase composition. Pristine HEO exhibited peaks at 30.38°, 35.72°, 40.43°, 57.37°, and 63.09°, corresponding to the (220), (311), (400), (511), and (440) planes of a cubic spinel structure (matched to CoCr_2_O_4_, PDF # 00-022-1084). It is worth noting that we performed Rietveld refinement comparisons between the CoCr_2_O_4_ spinel oxide and the HEO (Fig. S30 and Table S5). The HEO exhibits significantly larger lattice constants and unit-cell volumes (Table S5). The changes in lattice constants reflect substantial lattice distortion, which arises from the random occupancy of six metal cations in the HEO. In addition, the notable variation in microstrain also accounts for the broadening of the superlattice diffraction peaks observed in the HEO. Pure TiO_2_ showed characteristic anatase peaks (PDF # 01-073-1764) at 25.28° (101), 37.93° (004), 48.18° (200), 54.05° (105), and 62.86° (204). Critically, the HEO@TiO_2_ pattern displayed superimposed peaks from both phases, confirming successful heterostructure formation without detectable impurities.

Raman spectroscopy (Fig. 1c) further validated the phases and revealed interfacial interactions. Pure TiO_2_ exhibited anatase vibrations at 144 cm^−1^ (E_g_), 280 cm^−1^ (B_1g_), 432 cm^−1^ (A_1g_), and 618 cm^−1^ (B_1g_).^48^ Pristine HEO showed peaks at 475.3 cm^−1^ (E_g_), 508.34 cm^−1^ (F_2g_), and 663.27 cm^−1^ (A_1g_), consistent with *Fd*3*m* spinel oxides.^36^ Notably, the metal–oxygen (M–O) vibrational bands broadened significantly, indicating interfacial Ti–O–M bond formation. This suggests strong electronic coupling conducive to charge transfer across the heterojunction.^49–51^

Nitrogen adsorption–desorption isotherms (Fig. 1d) for TiO_2_, HEO, and HEO@TiO_2_ all exhibit type-IV behaviour with distinct hysteresis loops, confirming mesoporous characteristics. The Brunauer–Emmett–Teller (BET) specific surface areas are 61.28, 102.15, and 127.68 m^2^ g^−1^, respectively, with the corresponding average pore diameters of 5.63, 8.75, and 12.02 nm (inset in Fig. 1d). The notably higher surface area and broader pore-size distribution of HEO@TiO_2_ provide abundant active sites, promote efficient formation/decomposition of Li_2_O_2_ (in relevant electrochemical contexts), and enhance light-harvesting capability.

Morphological features were examined by scanning electron microscopy (SEM) and transmission electron microscopy (TEM). SEM images (Fig. 1e and f) reveal that the HEO@TiO_2_ composite comprises numerous TiO_2_ nanoparticles uniformly anchored onto the dendritic, porous framework of HEO, yielding a distinctly rough and hierarchical morphology. In contrast, pure HEO exhibits smooth porous dendrites (Fig. S2), while pure TiO_2_ consists of aggregated nanoparticles (Fig. S3). This unique composite architecture substantially increases the interfacial area, favouring efficient charge transfer.

TEM analysis (Fig. 1g) confirms intimate attachment of TiO_2_ nanoparticles to the HEO surface, forming a well-defined heterostructure. High-resolution TEM (HRTEM) imaging (Fig. 1h) and the corresponding fast Fourier transform (FFT) pattern (Fig. 1i) demonstrate that the (101) plane of anatase TiO_2_ is oriented at approximately 20° relative to the (211) plane of the spinel HEO, resulting in a near-coherent interface with minimal dislocations. Such lattice alignment significantly diminishes interfacial defect density and consequently suppresses the formation of interface states, thereby facilitating the establishment of a near-ideal heterojunction.

The HAADF-STEM image (Fig. 1j) combined with elemental mapping shows a uniform distribution of Fe, Co, Ni, Mn, Zn, Cr, and O throughout the HEO core, while Ti is predominantly enriched on the surface, forming a thin yet uniform coating. These observations align with semi-quantitative SEM-EDS results (Table S1) and ICP-OES elemental analysis (Table S2), which confirm near-equimolar ratios of the transition metals in HEO and a relatively low-coverage TiO_2_ layer. Selected-area electron diffraction (SAED) patterns (Fig. S4) display polycrystalline rings with *d*-spacings of 0.350, 0.251, 0.208, 0.169, 0.160, and 0.136 nm, corresponding to the (311), (400), and (511) planes of spinel HEO and the (101), (105), and (116) planes of anatase TiO_2_, in excellent agreement with the XRD findings.

Collectively, these analyses confirm the successful synthesis of a high-quality HEO@TiO_2_ heterostructure with intimate interfacial contact, enhanced surface area, and optimal structural features for advanced applications.

### 2.2. Theoretical calculation of M–O covalent bond enhancement at the HEO@TiO_2_ interface

To elucidate the electronic structure evolution and interfacial M–O bonding changes upon HEO loading onto TiO_2_, systematic density functional theory (DFT) calculations were conducted. Model surfaces comprised the HEO (101) and TiO_2_ (200) facets. Surface dangling bonds, known to enhance electronic activity, suggest a potential bandgap narrowing or closure, as indicated by the total density of states (TDOS).^52,53^ Critically, the electronic density of states near the Fermi level (*E*_f_) for HEO@TiO_2_ significantly exceeds those of pristine HEO and TiO_2_ (Fig. 2b), implying substantially improved electrical conductivity. Furthermore, the d-band center of HEO@TiO_2_ shifts to −0.897 eV, positioned closer to *E*_f_ than those of HEO (−1.969 eV) and TiO_2_ (−2.331 eV). This shift signifies enhanced adsorption capability for O_2_ and the LiO_2_ intermediate, thereby facilitating accelerated electron transfer and modulated Li_2_O_2_ nucleation.^54^

**Fig. 2:**
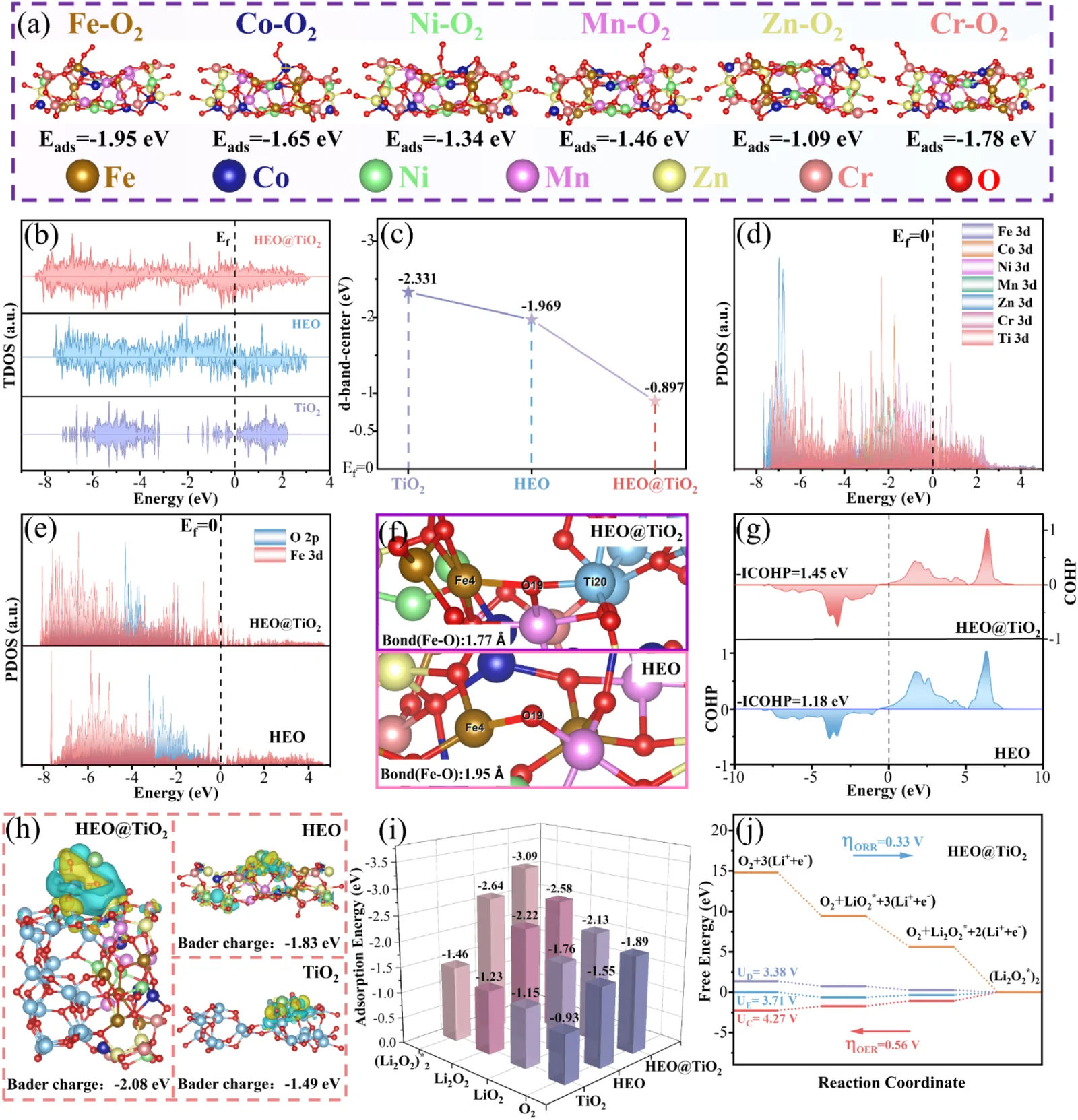
DFT calculation. (a) Adsorption configurations of O_2_ molecules on different metal active sites of the HEO surface and the corresponding adsorption energies. (b) TDOS for HEO@TiO_2_, HEO, and TiO_2_. (c) Comparison of d-band centre positions. (d) PDOS for HEO@TiO_2_. (e) Comparison of PDOS for Fe-3d and O-2p orbitals in HEO@TiO_2_ and HEO. (f) Changes in Fe–O bond lengths in HEO and HEO@TiO_2_. (g) COHP analysis of Fe–O bonds in HEO and HEO@TiO_2_ after oxygen adsorption. (h) Differential charge density distribution models for the interaction of HEO@TiO_2_, HEO, and TiO_2_ with the LiO_2_ intermediate. (i) Comparison of adsorption energies for different species (O_2_, LiO_2_, Li_2_O_2_, and the 
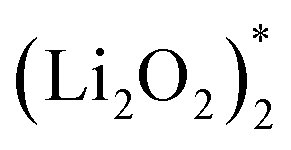
 dimer transition state). (j) Free energy diagram for the redox reactions on the HEO@TiO_2_ surface.

Strong electronic coupling at the heterointerface is evidenced by extensive d-orbital overlap among the constituent metal species in HEO@TiO_2_ (Fig. 2d). Notably, Ti 3d orbitals exhibit significant broadening across an energy range (−7.88 eV to +2.56 eV) that nearly encompasses the entire d-orbital region of the HEO metals. This observation positions Ti as an effective electron regulation center, promoting interfacial electron transport.^55^ Concurrently, the formation of a broad Ti 3d band crossing *E*_f_ predicts a substantially reduced energy barrier for electron transfer to LiO_2_ and O_2_ adsorbed on the HEO@TiO_2_ surface.

Given that Fe sites in HEO demonstrate the most favorable O_2_ adsorption energy (Fig. 2a), the Fe–O bond was selected to probe the M–O bonding mechanism. Projected density of states (PDOS) analysis (Fig. 2e) reveals that heterojunction formation with TiO_2_ markedly increases the overlap region between Fe-3d and O-2p orbitals, indicating enhanced orbital hybridization. This hybridization, driven by TiO_2_-induced charge redistribution, promotes Ti–O–Fe bond formation. Consequently, the interfacial strength distribution of O-centered Ti–O and Fe–O bonds is reconfigured, enhancing M–O covalency.^56^

Bond length analysis further corroborates this covalent strengthening (Fig. 2f and S5). The Fe–O bond shortens from 1.95 Å in pristine HEO to 1.77 Å in HEO@TiO_2_, with analogous trends observed for other M–O bonds. To further evaluate the Fe–O bond character post-O_2_ adsorption, Crystal Orbital Hamilton Population (COHP) analysis was employed (Fig. 2g). The integrated −ICOHP value for HEO@TiO_2_ (1.45 eV) surpasses that of HEO (1.18 eV), indicating reduced antibonding state occupancy, enhanced Fe–O_2_ orbital overlap, and ultimately, stronger M–O bond formation.^57^ Collectively, these results demonstrate that the HEO@TiO_2_ heterojunction establishes an efficient M–O electron transfer channel, promoting electron exchange between cations and oxygen species and significantly boosting ORR/OER catalytic activity.

The adsorption energy (Δ*E*_ads_) of oxygen species critically determines cathodic catalytic performance in LOBs. HEO@TiO_2_ exhibits stronger adsorption for all oxygen-containing intermediates compared to HEO or TiO_2_ alone (Fig. 2i). Reaction pathway analysis confirms that HEO@TiO_2_ possesses the highest O_2_ adsorption energy, favouring the 
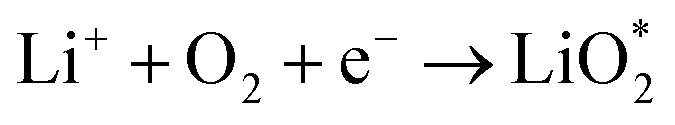
 reaction. Moreover, the Δ*E*_ads_ of LiO_2_ (−1.905 eV on HEO@TiO_2_*vs.* −1.065 eV on HEO and −1.473 eV on TiO_2_) not only influences the ORR but also critically regulates Li_2_O_2_ nucleation and growth.^58^ Similarly, higher adsorption energies for Li_2_O_2_ and (Li_2_O_2_)_2_ on HEO@TiO_2_ signify strong intermediate affinity, promoting uniform nucleation and reversible decomposition, thereby enhancing electrochemical performance.

Gibbs free energy profiles for the ORR/OER on HEO@TiO_2_ (Fig. 2j) identify the primary discharge rate-limiting steps as1
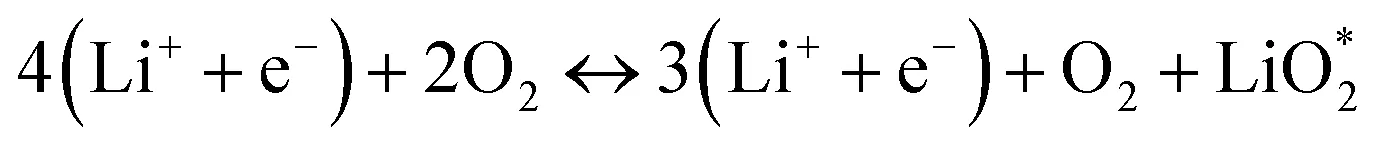
2
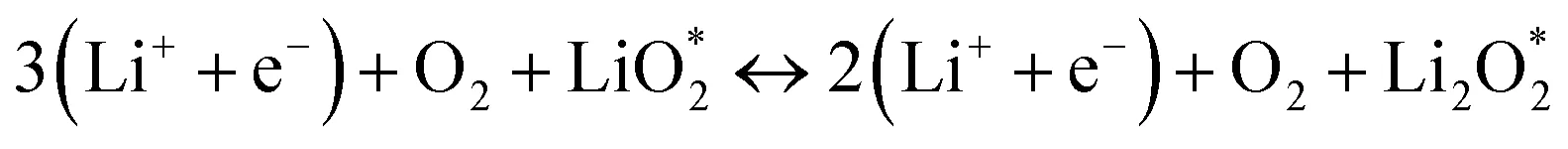
3
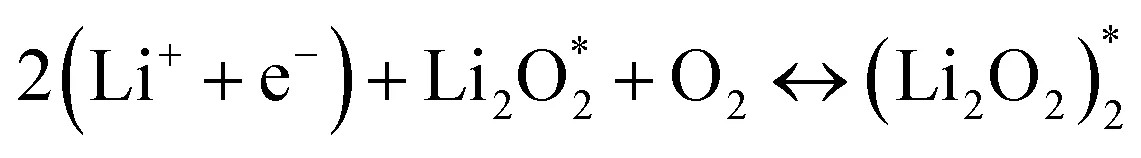


Among them, step 
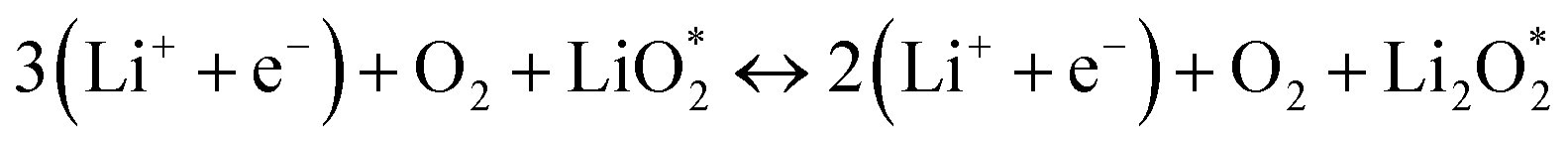
 is the primary rate-limiting step during discharge, while 
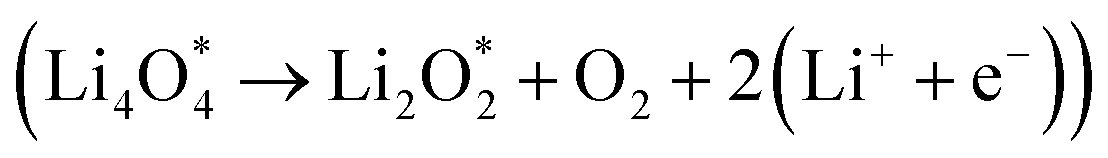
 is the main rate-limiting step during charge. The discharge and charge overpotentials are defined as *η*_ORR_ = *U*_D_ − *U*_E_ and *η*_OER_ = *U*_C_ − *U*_E_, where *U*_E_, *U*_D_, and *U*_C_ represent the equilibrium potential, discharge potential, and charge potential, respectively.^59,60^ HEO@TiO_2_ exhibits significantly lower discharge (Δ*η* = 0.33 eV *vs.* 0.42 eV for HEO and 0.51 eV for TiO_2_; Fig. S6a and b) and charge (Δ*η* = 0.56 eV *vs.* 0.63 eV for HEO and 0.71 eV for TiO_2_) overpotentials. This minimal overall overpotential (*U*_C_ − *U*_D_) underscores that the enhanced M–O electron channel not only strengthens intermediate adsorption but also markedly accelerates electron transfer kinetics. Complementing this, differential charge density analysis (Fig. 2h) reveals pronounced charge redistribution (yellow: accumulation; blue: depletion) between HEO@TiO_2_ and LiO_2_, with significantly greater interfacial charge transfer compared to HEO or TiO_2_, further confirming superior electron transport capability.

Overall, the consistent evidence from DOS, COHP, differential charge density, and Gibbs free energy calculations demonstrates that the HEO@TiO_2_ heterojunction optimizes the electronic structure *via* interfacial coupling, weakens Ti–O bonds while strengthening M–O covalency, and enhances adsorption of key reaction species. These effects collectively improve interfacial electron transport and reduce ORR/OER energy barriers, providing a robust theoretical foundation for designing high-performance bifunctional catalytic cathodes enabling LOBs with enhanced electrochemical performance.

### 2.3. Verification of interfacial M–O covalent bond enhancement by synchrotron radiation

To validate the theoretically calculated enhancement of M–O bond covalency, synchrotron radiation X-ray absorption fine structure (XAFS) spectroscopy was employed to probe the electronic structure and local coordination of Fe in pristine high-entropy oxide (HEO) and HEO@TiO_2_ heterostructures. Fig. 3a compares the Fe K-edge XANES spectra of HEO and HEO@TiO_2_ with those of reference compounds (Fe_2_O_3_, Fe_3_O_4_, and Fe foil). The absorption threshold energy, determined from spectral first derivatives, progressively shifts from 7131.5 eV (Fe_3_O_4_) to 7132.6 eV (HEO) and 7133.4 eV (HEO@TiO_2_). This positive energy shift indicates a higher average Fe oxidation state in HEO@TiO_2_, implying reduced Fe 3d-orbital occupancy and an elevated d-band center. Such electronic perturbation originates from interfacial charge redistribution: electrons transfer from HEO to TiO_2_ across the heterojunction to equilibrate Fermi levels, generating a built-in electric field.

**Fig. 3:**
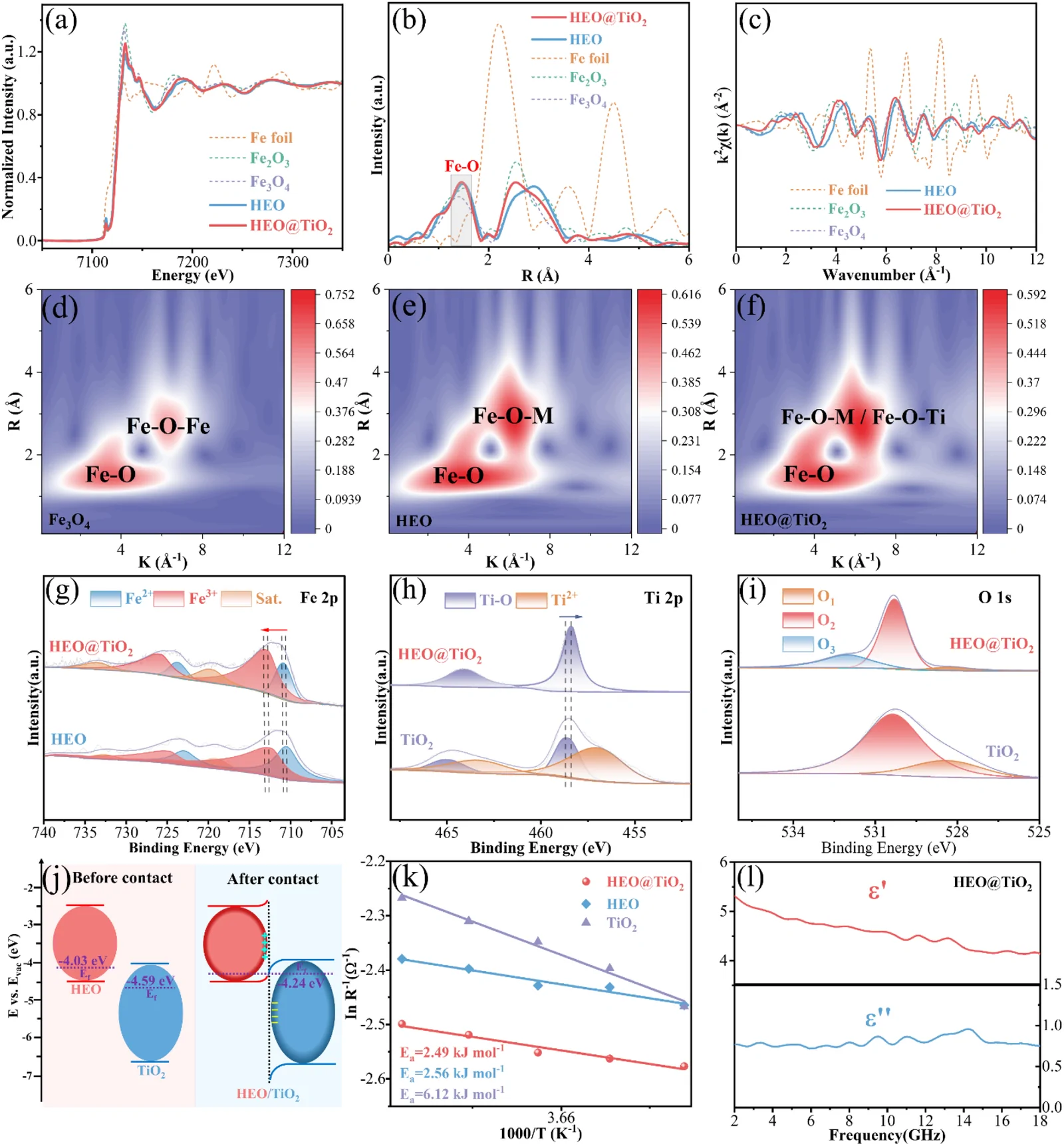
Analysis of synchrotron radiation and energy bands. (a) Fe K-edge XANES spectra of HEO, HEO@TiO_2_, and reference samples (Fe_2_O_3_ and Fe_3_O_4_). (b) Corresponding FT-EXAFS spectra and (c) EXAFS oscillation signals (*k*^2^-weighted *χ*(*k*)). (d–f) WT-EXAFS contour plots for Fe_3_O_4_, HEO, and HEO@TiO_2_. (g) Comparison of high-resolution Fe 2p XPS spectra for HEO and HEO@TiO_2_. Comparison of (h) Ti 2p and (i) O 1s XPS spectra for TiO_2_ and HEO@TiO_2_. (j) Schematic illustration of *E*_f_ changes upon heterojunction formation between HEO and TiO_2_. (k) Comparison of intrinsic activation energies for HEO, TiO_2_, and HEO@TiO_2_. (l) Microwave dielectric properties (real and imaginary parts of permittivity) of HEO@TiO_2_ measured using a vector network analyser.

Fig. 3b presents the Fourier-transform extended X-ray absorption fine structure (FT-EXAFS) spectra for HEO and HEO@TiO_2_. Both exhibit a prominent main peak corresponding to the first coordination shell of Fe–O bonds. Fitting results for the Fe–O bond lengths in the respective samples reveal average values of 1.52 Å for Fe_3_O_4_, 1.49 Å for HEO, and 1.45 Å for HEO@TiO_2_. The significant shortening of the Fe–O bond length in HEO@TiO_2_ indicates that the introduction of Ti induces substantial reconstruction of the local Fe–O–Ti structure and markedly enhances the covalent character of the Fe–O bond. Further comparison of the EXAFS oscillation curves in *k*-space (Fig. 3c) shows that both HEO and HEO@TiO_2_ exhibit higher oscillation frequencies than Fe_2_O_3_ and Fe_3_O_4_, reflecting their shorter average bond lengths. In the high-*k* region (approximately 8–11 Å^−1^), the oscillation amplitude of HEO@TiO_2_ is slightly higher than that of HEO, suggesting lower structural disorder (smaller Debye–Waller factor *σ*^2^) and an increase in the first-shell coordination number (details in Table S4). Collectively, these results demonstrate that HEO@TiO_2_ possesses a more ordered local atomic arrangement. The strengthening of the M–O bonds effectively mitigates the inherent lattice distortion of the high-entropy oxide, thereby significantly enhancing the overall structural stability.

Wavelet transform (WT) analysis of the EXAFS data offers complementary two-dimensional *k*–*R* resolved visualization (Fig. 3d–f). The WT contour map of Fe_3_O_4_ resolves two distinct intensity maxima at *k* ≈ 3.45 Å^−1^ and 6.28 Å^−1^, attributable to the Fe–O single-scattering path and the Fe–O–Fe multiple-scattering path, respectively. In contrast, the WT map of HEO@TiO_2_ reveals an additional characteristic feature (Fig. 3f) corresponding to the newly formed interfacial Fe–O–Ti coordination pathway. Direct comparison of the WT contours for HEO and HEO@TiO_2_ (Fig. 3e and f) shows that the primary Fe–O peak remains centered at *k* ≈ 3.48 Å^−1^ in both cases, whereas the secondary peak shifts from *k* ≈ 6.35 Å^−1^ in HEO (predominantly Fe–O–M) to a more concentrated and slightly shifted contribution associated with Fe–O–Ti in HEO@TiO_2_. Moreover, the maximum intensity of the Fe–O path decreases from 0.612 in HEO to 0.582 in HEO@TiO_2_, confirming that interfacial modulation suppresses lattice distortion, promotes higher structural ordering, increases the oxygen coordination number around Fe sites, and stabilizes the atomic configuration.

It is worth noting that we selected Fe as the representative active site for the DFT model based on its optimal O_2_ adsorption energy and its strong spectral fingerprint features at the K-edge in XAFS.

Moreover, the observed interfacial modulation—particularly the enhanced M–O covalency and charge redistribution—is mutually corroborated by the enhanced 3d–2p multi-orbital hybridization in DFT calculations, the results of XAFS analysis, and the consistent chemical-state changes of Co, Ni, Mn, Zn, and Cr observed in XPS analysis (Fig. S7). In combination with the “cocktail effect” of the HEO material, this effectively enhances the reaction kinetics of the bifunctional ORR/OER in LOBs.

### 2.4. Construction of the HEO@TiO_2_ S-Scheme heterojunction with the reverse-blocking layer and interfacial charge regulation mechanism

To elucidate interfacial chemical state evolution and band structure modulation in the HEO/TiO_2_ heterojunction, we conducted systematic XPS analyses of key elements (Fe, Co, Ni, Mn, Zn, Cr, O, and Ti). The high-resolution Fe 2p spectrum of pristine HEO (Fig. 3g) resolves into Fe^2+^ (710.74 eV and 723.08 eV), Fe^3+^ (712.26 eV and 725.70 eV), and satellite peaks (718.8 eV and 732.6 eV).^43^ In HEO@TiO_2_, Fe 2p peaks exhibit a pronounced negative shift alongside a significantly increased Fe^3+^/Fe^2+^ ratio, indicating interfacial oxidation of Fe^2+^ to Fe^3+^. Fe^3+^ possesses a higher effective nuclear charge than Fe^2+^, strengthening electron binding. According to Pauling's electronegativity principle,^56^ this reduced electronegativity difference enhances bond covalency. Electronically, Fe^3+^ has one fewer d-electron than Fe^2+^, shifting its d-band center toward the Fermi level and reducing O 2p orbital repulsion. This promotes stronger Fe 3d-orbital and O 2p-orbital hybridization, underpinning enhanced covalency.^61^ Furthermore, high-spin Fe^3+^ adopts a half-filled t_2g_ configuration, enabling dual π-electron donor/acceptor functionality: e_g_ orbitals form σ-coordinate bonds (Fe → O) with O 2p, while O 2p π electrons back-donate into vacant t_2g_ orbitals (O → Fe), reinforcing covalency. Consequently, TiO_2_ compositing elevates the HEO Fe^3+^/Fe^2+^ ratio. The resultant Ti–O–Fe interfacial bridges redistribute bond strengths, weakening Ti–O bonds while intensifying Fe–O covalency. Critically, similar trends emerge for Co, Ni, Mn, Zn, and Cr (Fig. S7): binding energies shift positively with increased higher-valent ion proportions, confirming multi-metal interfacial oxidation. Higher valency universally enhances M–O covalency per Pauling's theory. Conversely, Ti 2p binding energy in TiO_2_ shifts negatively post-heterojunction (Fig. 3h), signifying Ti^4+^ reduction despite dominant Ti–O stability. The O 1s spectrum (Fig. 3i) corroborates charge transfer: pristine TiO_2_ shows peaks at 529.7 eV (Ti–O lattice oxygen) and 531.6 eV (oxygen vacancies), while HEO@TiO_2_ exhibits an additional peak at 533.2 eV (surface-adsorbed oxygen), confirming Ti–O–M bridge formation. Collectively, these systematic binding energy shifts demonstrate substantial interfacial charge redistribution, manifesting as net electron transfer from HEO to TiO_2_. Ti–O–M bridges optimize metal–oxygen bond covalency and lengths, thereby enhancing oxygen intermediate adsorption/desorption energetics for improved catalytic activity. Band alignment analysis *via* UV-Vis/Tauc plots (indirect transition, *n* = 4) yields bandgaps of 1.86 eV (HEO) and 2.83 eV (TiO_2_) (Fig. S8a and b).^62^ Mott–Schottky measurements confirm p-type HEO (*E*_fb_ = −0.5 V *vs.* NHE) and n-type TiO_2_ (*E*_fb_ = −0.1 V *vs.* NHE) (Fig. S9a–c). Converted to the vacuum scale (*E*_vac_ = −*E*_NHE_ − 4.44), Fermi levels are 4.14 eV (HEO) and 4.54 eV (TiO_2_). Theoretical work functions align closely: 4.03 eV (HEO) and 4.59 eV (TiO_2_) (Fig. S10a and b). Upon heterojunction formation, Fermi level alignment establishes a composite *E*_fb_ = −0.4 V (W* > ≈4.24 eV). As depicted in Fig. 3j, the lower work function of HEO positions its Fermi level above that of TiO_2_. After contact, electrons transfer spontaneously from HEO to TiO_2_ until *E*_f_ reaches equilibrium, creating a built-in electric field directed toward TiO_2_. This accumulates holes at the p-HEO interface and electrons at the n-TiO_2_ interface, forming multi-carrier accumulation layers on both sides, termed the “reverse-blocking layer”. Unlike conventional depletion barriers, this accumulation-type structure enables highly efficient interfacial charge conduction.

This mechanism is directly evidenced by activation energy analysis (Fig. 3k). Over the temperature range of 265–285 K, electrochemical impedance data at five selected average temperatures were analyzed *via* the Arrhenius equation *k* = *A*e^(−*E*_a_/*RT*)^ (where *k* is the rate constant, *A* the pre-exponential factor, *E*_a_ the activation energy, *R* the gas constant, and *T* the absolute temperature), with ln(*R*_ct_^−1^) plotted against 1/*T*. The slope yields *E*_a_. The HEO@TiO_2_ heterojunction exhibits significantly lower *E*_a_ compared to pristine HEO and TiO_2_, demonstrating that the reverse-blocking layer substantially reduces the interfacial reaction energy barrier. Furthermore, microwave vector network analysis (Fig. 3l) provides the real (*ε*′) and imaginary (*ε*″) dielectric components for HEO@TiO_2_, yielding an average dielectric constant modulus |*ε*| = 6.455 across 2–18 GHz. Using the Mott–Schottky carrier concentration formula:
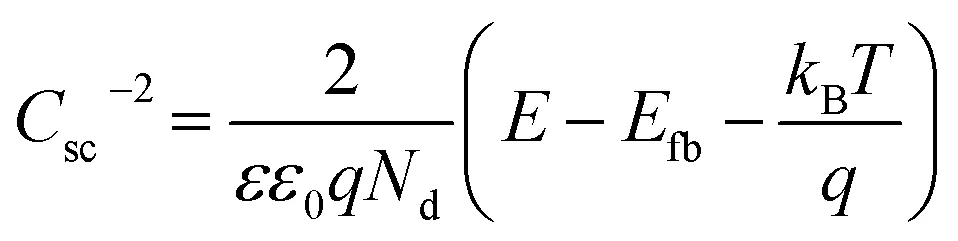
where *N*_d_ is the carrier density, *C*_sc_ is the space charge capacitance, *ε* is the dielectric constant, *ε*_0_ is the vacuum permittivity, *q* is the elementary charge, *E*_fb_ is the flat-band potential, *k*_B_ is the Boltzmann constant, *T* is the absolute temperature, and *E* is the electrode potential. Combined with the Mott–Schottky slope |*k*| = 2.065 from Fig. S9c, the carrier density for HEO@TiO_2_ is calculated to be 1.057 × 10^19^ m^−3^. Comparative analyses based on Mott–Schottky slopes (Fig. S9a and b) and microwave data (Fig. S11a–d) yield carrier densities of 2.863 × 10^18^ m^−3^ for pristine HEO and 2.792 × 10^18^ m^−3^ for pristine TiO_2_.

Based on the above, the work function difference between HEO and TiO_2_ drives the electron flow from the HEO to TiO_2_ upon their contact, promoting the Fermi levels to reach equilibrium and thereby establishing a built-in electric field directed from the HEO to TiO_2_. Unlike conventional p–n junctions that form a depletion layer, the Fermi-level equilibrium here leads to the accumulation of majority carriers on both sides of the interface—holes accumulate on the p-type HEO side and electrons accumulate on the n-type TiO_2_ side. This unique configuration is what we term a “reverse barrier layer”. The substantial carrier accumulation at the interface significantly reduces the interfacial resistance, enabling bidirectional charge transport and exhibiting ohmic-contact-like behaviour. This is corroborated by the high carrier densities obtained from Mott–Schottky and microwave dielectric analyses. The built-in electric field generated by this reverse barrier layer configuration serves as the driving force for the S-scheme charge transfer, providing the foundation for the subsequent S-scheme charge-transfer pathway under light irradiation.

### 2.5. Electrochemical performance of HEO@TiO_2_ as a LOB cathode in the dark

Galvanostatic charge–discharge tests at 200 mA g^−1^ (Fig. 4a) demonstrate that the HEO@TiO_2_ cathode delivers an exceptional discharge capacity of 29 545 mA h g^−1^ under dark conditions. This value significantly surpasses those of pure HEO (22 055 mA h g^−1^) and TiO_2_ (9965 mA h g^−1^) cathodes. An initial coulombic efficiency exceeding 98% further confirms the superior electrochemical reversibility of HEO@TiO_2_ materials. Critically, this performance advantage persists at higher current densities (600 and 1000 mA g^−1^) (Fig. S12a and b). Capacity retention data (Fig. 4b) corroborate its enhanced rate capability: HEO@TiO_2_ retains 72% and 61% of its capacity at 600 and 1000 mA g^−1^, respectively, markedly outperforming HEO (68%, 54%) and TiO_2_ (57%, 45%). CV within 2.0–4.5 V (Fig. 4c) reveals that the HEO@TiO_2_ cathode exhibits the highest cathodic reduction and anodic oxidation peak currents, indicating optimal ORR and OER kinetics. Consequently, at 200 mA g^−1^ and a fixed capacity of 1000 mA h g^−1^, HEO@TiO_2_ achieves a first-cycle charge–discharge overpotential of just 0.94 V (Fig. 4d), substantially lower than those of HEO (1.16 V) and TiO_2_ (1.38 V). This trend holds at 600 and 1000 mA g^−1^ (Fig. S13a and b).

**Fig. 4:**
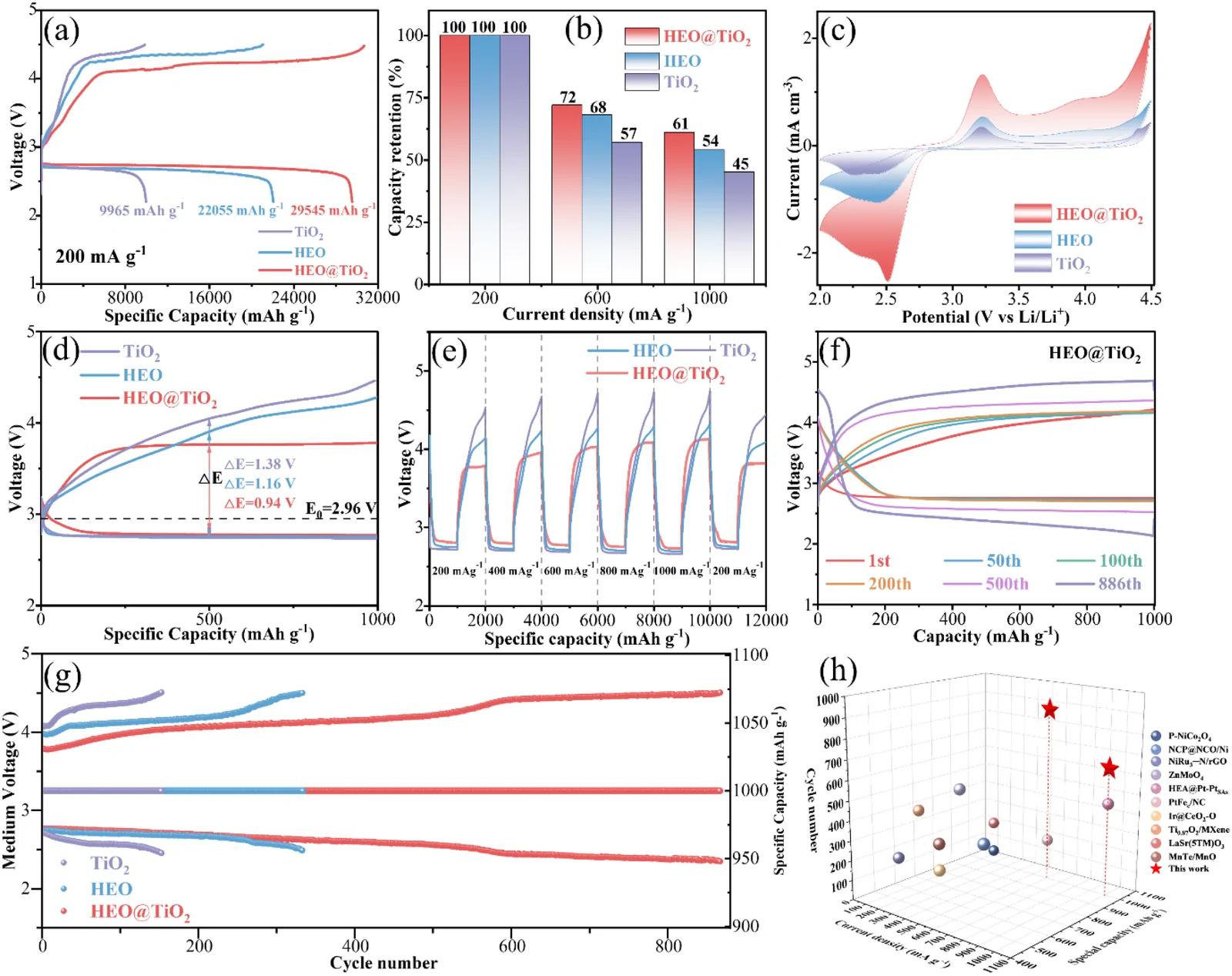
Electrochemical performance. (a) initial charge–discharge profiles of different electrodes at 200 mA g^−1^. (b) Capacity retention at 600 and 1000 mA g^−1^. (c) Comparison of CV curves (scan rate: 0.01 mV s^−1^). (d) First-cycle overpotential at 200 mA g^−1^ with a limited capacity of 1000 mA h g^−1^. (e) Rate capability measurement. (f) Selected charge–discharge profiles of the HEO@TiO_2_ electrode at 600 mA g^−1^ with a limited capacity of 1000 mA h g^−1^. (g) Long-term cycling performance of different electrodes. (h) Comparison of cycle number for HEO@TiO_2_ with reported electrocatalysts at different current densities and cut-off capacities.

Long-term cycling stability tests further highlight the superior durability of HEO@TiO_2_ (Fig. 4g). Under conditions of 600 mA g^−1^ current density, 1000 mA h g^−1^ fixed capacity, and 2.0–4.5 V voltage range, the HEO@TiO_2_ cathode sustains stable operation for 867 cycles—far surpassing HEO (334 cycles) and TiO_2_ (168 cycles). Even at a higher current density of 1000 mA g^−1^, stable cycling persists for 639 cycles (Fig. S14). Consistently, overpotential evolution (Fig. 4e, S15a and b) shows only a gradual increase for HEO@TiO_2_, whereas HEO and TiO_2_ rapidly approach the 4.5 V charge cut-off. Cycling profiles at 1000 mA g^−1^ (Fig. S16a–c) further validate this stability. Notably, comparative analysis with state-of-the-art cathodes (Fig. 4h and Table S3) confirms the superior performance of HEO@TiO_2_ at both 600 and 1000 mA g^−1^.

Rate capability evaluations (Fig. 4f) demonstrate that, under stepwise increases in current density from 200 to 1000 mA g^−1^ at a fixed capacity of 1000 mA h g^−1^, the charge–discharge voltage plateaus of HEO@TiO_2_ remain consistently lower than those of HEO and TiO_2_. In conjunction with the overpotential results, these findings indicate that the multi-element, multi-channel Ti–O–M surface bonding configuration, coupled with enhanced M–O covalency, effectively mitigates battery polarization.

Collectively, the HEO@TiO_2_ heterojunction cathode, engineered with a reverse blocking layer, significantly enhances LOB performance, concretely manifested in elevated capacities, minimized polarization, and extended cycling stability. This improvement stems directly from synergistic enhancement of bifunctional ORR/OER activity, consistent with the proposed M–O enhancement mechanism. Meanwhile, the built-in electric field at the heterojunction interface promotes efficient charge carrier separation and transport, thereby accelerating redox kinetics and boosting electrocatalytic performance.

### 2.6. *In situ* and *ex situ* characterization of the LOB cathode electrode in the dark

To elucidate the enhanced redox kinetics observed in electrochemical tests, we conducted comprehensive *in situ* and *ex situ* characterization of the HEO@TiO_2_ cathode. *In situ* electrochemical impedance spectroscopy (EIS) during galvanostatic cycling revealed characteristic trends in charge transfer resistance (*R*_ct_). The discharge-induced formation of insulating Li_2_O_2_ increased *R*_ct_, while its decomposition during charging reduced it. DRT analysis with corresponding contour plots (Fig. 5a and d) further resolved distinct resistive contributions migration through SEI), *τ*_3_ (Li_2_CO_3_ decomposition), and *τ*_4_ (Li_2_O_2_ based on relaxation time constants (*τ*): *τ*_1_ (contact resistance), *τ*_2_ (Li^+^ decomposition). Critically, in the *τ*_3_ regime (∼2.2 V), HEO@TiO_2_ exhibited significantly lower Li_2_CO_3_ decomposition impedance than pristine HEO or TiO_2_ (Fig. S17a and c), consistent with suppressed Li_2_CO_3_ formation. Moreover, during *τ*_4_, HEO@TiO_2_ showed a smooth, progressive decrease in Li_2_O_2_ decomposition impedance, approaching zero at 4.5 V. In stark contrast, both HEO and TiO_2_ electrodes displayed pronounced *τ*_4_ impedance fluctuations during charging, indicating overlapping Li_2_CO_3_ decomposition, and substantial residual impedance, confirming inferior rechargeability. *Ex situ* XRD of the fully discharged HEO@TiO_2_ electrode confirmed Li_2_O_2_ formation *via* (100), (101), and (110) reflections (Fig. 5b). Notably, these peaks vanished completely after charging and remained absent even after 100 cycles, demonstrating exceptional reversibility. Conversely, while HEO and TiO_2_ electrodes showed Li_2_O_2_ peaks upon discharge, residual signals persisted after 1, 50, and 100 cycles (Fig. S18a and c), indicating sluggish OER kinetics and limited rechargeability—aligning with EIS results. *Ex situ* XPS analysis (Fig. 5e) provided deeper mechanistic insights. The Li 1s spectrum of discharged HEO@TiO_2_ was dominated by Li_2_O_2_ (55.4 eV), with only a minor Li_2_CO_3_ contribution (56.4 eV).^63^ Strikingly, no Li_2_O_2_ or Li_2_CO_3_ signals were detected after any charging cycle, reaffirming high reversibility. For comparison, pristine HEO and TiO_2_ exhibited higher Li_2_CO_3_/Li_2_O_2_ ratios after initial discharge, signifying severe side reactions. Furthermore, Li_2_CO_3_ accumulated progressively on both electrodes during cycling, while Li_2_O_2_ decomposition remained incomplete (Fig. S18b and d). The primary source of Li_2_CO_3_ is the reaction of Li^+^ ions with carbonate ions (CO_3_^2−^) derived from the decomposition of the unstable electrolyte during charge/discharge (Li^+^ + CO_3_^2−^ ⇌ Li_2_CO_3_), impeding the complete reversible decomposition of Li_2_O_2_.^64^

**Fig. 5:**
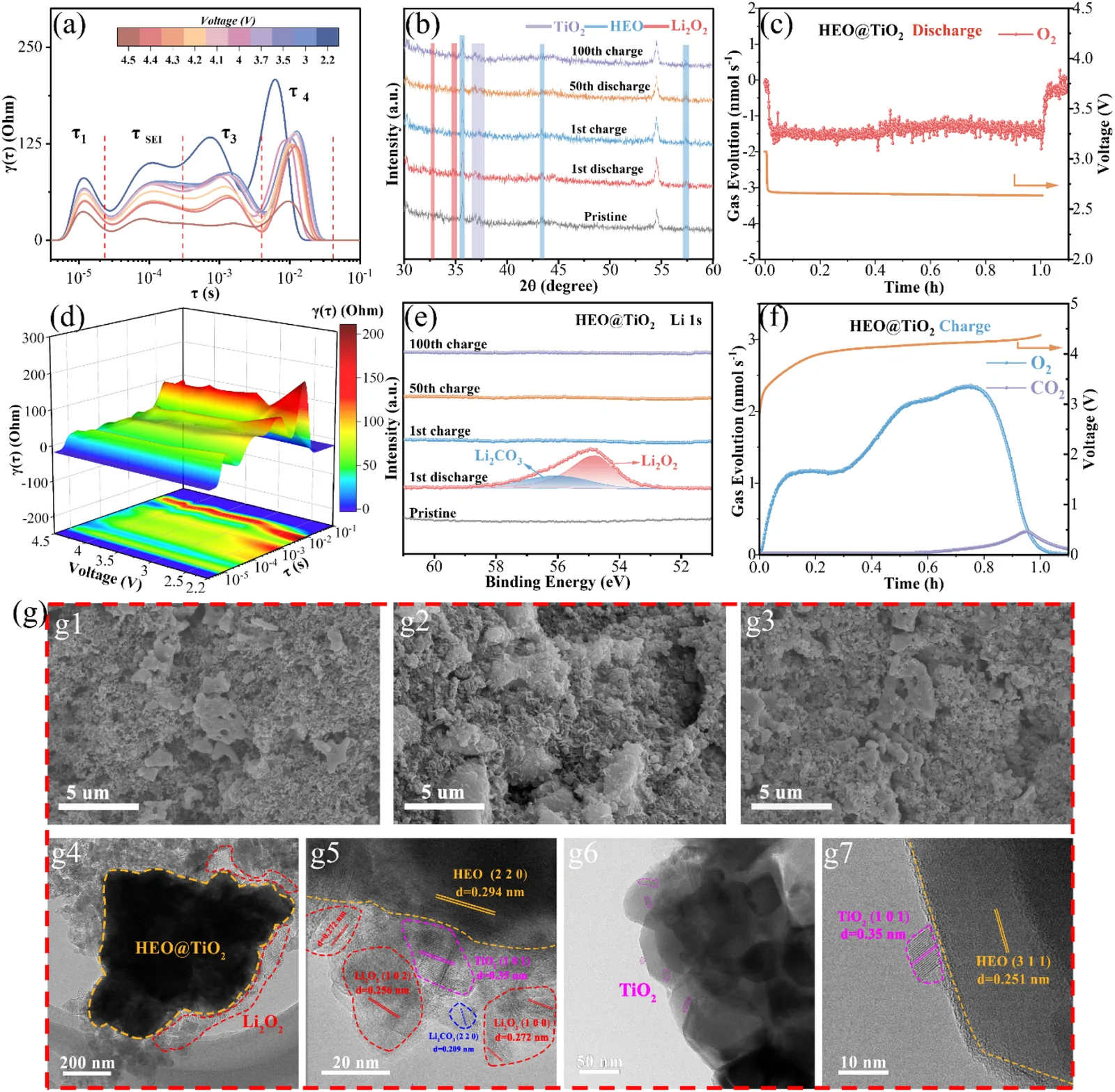
*In situ* and *ex situ* characterization in the dark. (a) and (d) *In situ* EIS Nyquist plots, corresponding DRT analysis, and contour plots for the HEO@TiO_2_ electrode. (b) XRD patterns and (e) XPS spectra of the HEO@TiO_2_ electrode at different charge–discharge stages. (c) and (f) DEMS analysis of the HEO@TiO_2_ electrode during discharge and recharge processes. (g1–g3) SEM images of the HEO@TiO_2_ electrode in the pristine, fully discharged, and recharged states. (g4–g7) TEM and HRTEM images of the electrode in the fully discharged and recharged states.

*In situ* differential electrochemical mass spectrometry (DEMS) quantified reaction reversibility *via* e^−^/O_2_ ratios (ideal = 2.0).^27,65^ During the ORR, HEO@TiO_2_ achieved a near-ideal ratio of 2.07 (Fig. 5c), confirming Li_2_O_2_ as the primary product with negligible side reactions. By contrast, HEO and TiO_2_ yielded higher ratios (2.15 and 2.49; Fig. S19a and c), indicating parasitic processes. Similarly, during the OER, HEO@TiO_2_ maintained a ratio of 2.05 with minimal CO_2_ evolution (Fig. 5f), evidencing near-complete Li_2_O_2_ decomposition. Conversely, HEO and TiO_2_ showed elevated ratios (2.24 and 2.58) with significant CO_2_ release (Fig. S19b and d), underscoring severe side reactions during charging.

The SEM and TEM results directly confirm the above conclusion from the perspectives of morphology and structure. The pristine HEO@TiO_2_ electrode displayed a clean, smooth surface (Fig. 5g1). Discharged HEO@TiO_2_ formed thin, flake-like Li_2_O_2_ deposits primarily on the catalyst surface (Fig. 5g2), which were fully removed upon charging, restoring the pristine morphology (Fig. 5g1 and g3). In contrast, the HEO electrode exhibited dense toroidal Li_2_O_2_ deposits on both the catalyst and carbon after discharge, with noticeable residues post-charging (Fig. S20a–d). The TiO_2_ electrode showed extensive blocky and flake-like products distributed across large areas, coupled with pronounced residuals after charging (Fig. S21a–d), underscoring limited reversibility.

Critically, TEM analysis of the discharged HEO@TiO_2_ electrode revealed flake-like Li_2_O_2_ with characteristic (101) and (102) facets, together with trace Li_2_CO_3_ (Fig. 5g4 and g5). Remarkably, after charging, these products vanished completely, preserving the distinct heterojunction interface and the epitaxial relationship between HEO (311) and TiO_2_ (101) planes (Fig. 5g6 and g7), indicative of outstanding structural reversibility. Even after 500 cycles, the heterostructure remained intact and well-defined (Fig. S22), highlighting exceptional structural stability that effectively accommodates the mechanical stresses from repeated Li_2_O_2_ formation/decomposition.

Collectively, the cross-characterization results from *in situ* EIS and DEMS and *ex situ* XRD, XPS, SEM and TEM unequivocally demonstrate that HEO@TiO_2_ significantly reduced parasitic reactions and achieves superior reversibility in dark environments. The distinctive heterostructure, based on the M–O covalency enhancement mechanism, establishes a “reverse-blocking layer”-like heterointerface, optimizing electronic conductivity and bifunctional ORR/OER kinetics, thereby achieving efficient bifunctional catalytic performance.

### 2.7. Optical characterization of TiO_2_, HEO, and HEO@TiO_2_

To evaluate the photo-assisted catalytic potential of the HEO@TiO_2_ S-scheme heterostructure, steady-state photoluminescence (PL) measurements were first performed. As shown in Fig. 6a, HEO@TiO_2_ exhibits the most pronounced PL quenching compared to pristine HEO and TiO_2_, indicating effective suppression of electron–hole recombination. Transient photocurrent and photovoltage measurements further reveal the highest photoresponse for HEO@TiO_2_ (Fig. 6b and S23), confirming enhanced mobility of photogenerated carriers. These results preliminarily suggest that the interfacial electric field of the S-scheme heterojunction, in synergy with reinforced M–O covalent bonds, efficiently facilitates charge separation and transport.

**Fig. 6:**
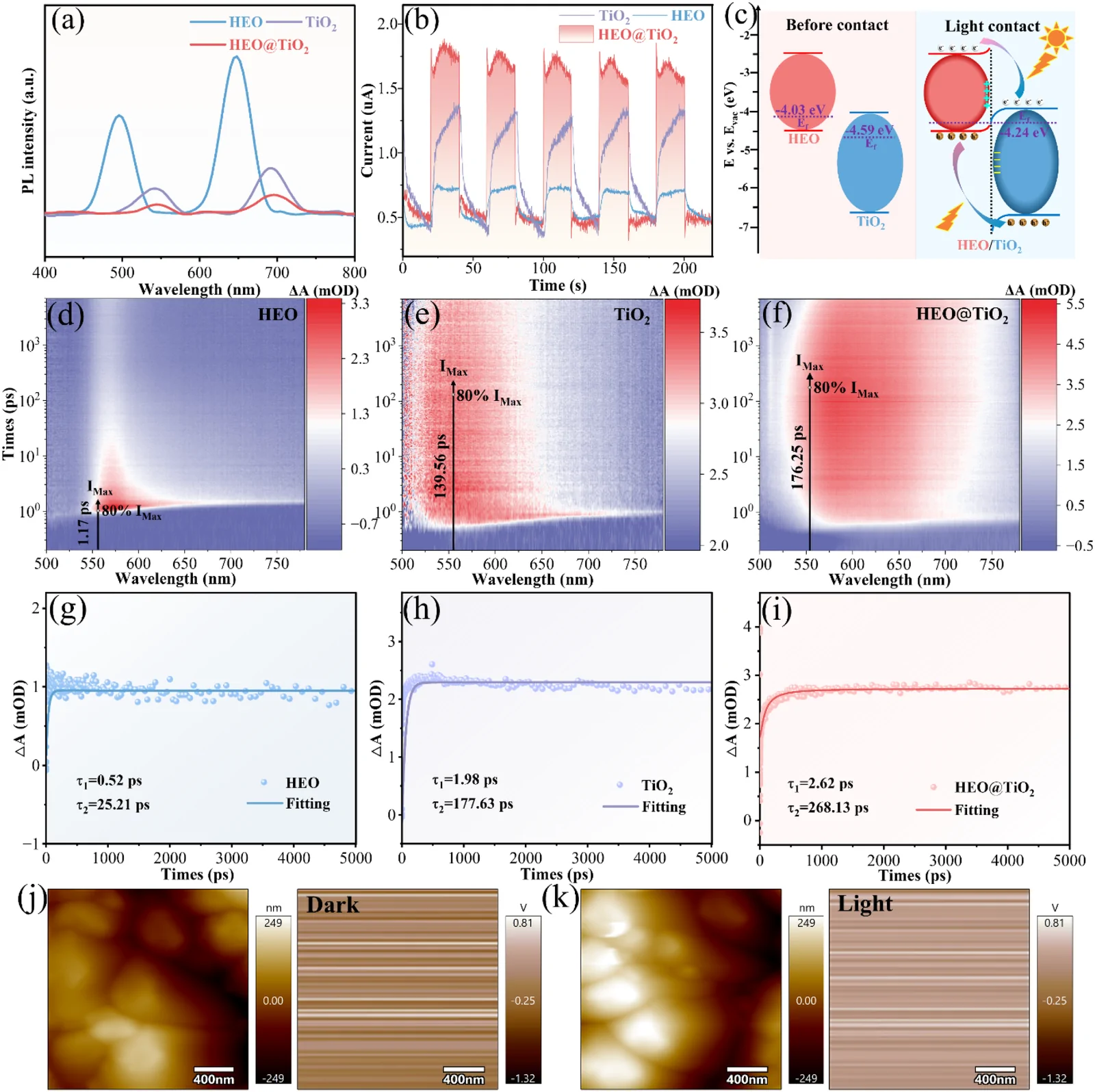
Optical characterization. (a) Photoluminescence measurements and (b) transient photocurrent responses of TiO_2_, HEO, and HEO@TiO_2_. (c) Schematic diagram of external light field-assisted HEO@TiO_2_. (d–f) 2D pseudocolor fs-TA spectra of TiO_2_, HEO, and HEO@TiO_2_. (g–i) TA kinetic plots and typical fitting curves of TiO_2_, HEO, and HEO@TiO_2_ at 555 nm. AFM topographic image and KPFM surface potential map of HEO@TiO_2_ (j) without and (k) with illumination.

To probe the ultrafast charge separation dynamics, femtosecond transient absorption (fs-TA) spectroscopy under 405 nm excitation was conducted. The two-dimensional fs-TA spectra (Fig. 3d–f) display a broad negative absorption band from 500 to 775 nm, corresponding to overlapping ground-state bleaching (GSB) and stimulated emission (SE) signals. The decay of these signals reflects the recombination of photogenerated carriers.^66^ Compared with HEO (1.17 ps) and TiO_2_ (139.56 ps), HEO@TiO_2_ exhibits an extended 80% decay lifetime of 176.25 ps, indicating that interfacial charge redistribution within the heterojunction effectively prolongs electron–hole recombination.

Biexponential fitting of the kinetic traces at 600 nm (Fig. 3g–i) resolves two electron quenching pathways: a short-lived component (*τ*_1_) assigned to electron diffusion on the crystal lattice and a long-lived component (*τ*_2_) corresponding to shallow charge trapping at surface/interface states. Notably, *τ*_2_ for HEO@TiO_2_ is significantly extended to 268.13 ps, far exceeding HEO (25.21 ps) and TiO_2_ (177.63 ps). This indicates prolonged charge excitation and transfer processes, enabling more active electrons to participate in the ORR/OER.

Overall, combining fs-TA with PL and transient photocurrent/voltage results demonstrates that the synergistic effect of the “reverse-blocking layer” in the S-scheme heterojunction and reinforced M–O covalent bonds not only enhances interfacial charge separation and extends carrier lifetimes, but also effectively harnesses photo-assisted activation to accelerate ORR/OER kinetics in Li–O_2_ batteries. As illustrated in Fig. 6c, under illumination, this field drives photogenerated holes toward TiO_2_ and electrons toward HEO, generating a distinct photovoltaic effect characteristic of an S-scheme heterojunction. This configuration preserves strong redox potentials, thereby endowing the material with enhanced catalytic activity. These results collectively underscore the excellent photovoltaic properties of HEO@TiO_2_ and its strong potential as a self-powered photoelectrode. Consequently, HEO@TiO_2_ will be selected as the cathode for photo-assisted LOBs in subsequent comparative analyses.

### 2.8. Photo-assisted electrochemical performance of HEO@TiO_2_ as a LOB cathode

Kelvin probe force microscopy (KPFM) was used to investigate the surface potential distribution of HEO@TiO_2_ before and after light illumination (Fig. 6j and k). Fig. 6j represents the topography and surface potential under dark conditions, showing a relatively uniform surface potential ranging from −1.32 V to 0.81 V. After light illumination (Fig. 6k), significant bright contrast emerges in the surface potential map, indicating enhanced local photovoltage and effective photogenerated charge separation, demonstrating that light exposure induces redistribution of photogenerated electrons and holes across the heterojunction interface. Under testing conditions of 200 mA g^−1^ current density and 1000 mA h g^−1^ limited specific capacity, standard solar illumination (AM 1.5G, 100 mW cm^−2^) significantly improved the electrochemical performance of the HEO@TiO_2_ cathode compared to operation in the dark. The discharge plateau voltage increased markedly from 2.90 to 3.37 V (+0.53 V), while the charge plateau voltage decreased from 3.86 to 3.52 V (−0.34 V). This resulted in an extremely low charge–discharge overpotential of approximately 0.15 V and a high energy round-trip efficiency of 97.7% (Fig. 7a). Notably, the magnitude of the discharge plateau elevation (0.53 V) was significantly greater than that of the charge plateau reduction (0.34 V). This indicates that the HEO component, benefiting from the synergistic cocktail effect of its multiple active sites, exhibits a more pronounced enhancement in reducing capability under illumination compared to the improvement in the oxidizing capability of the TiO_2_ component.

**Fig. 7:**
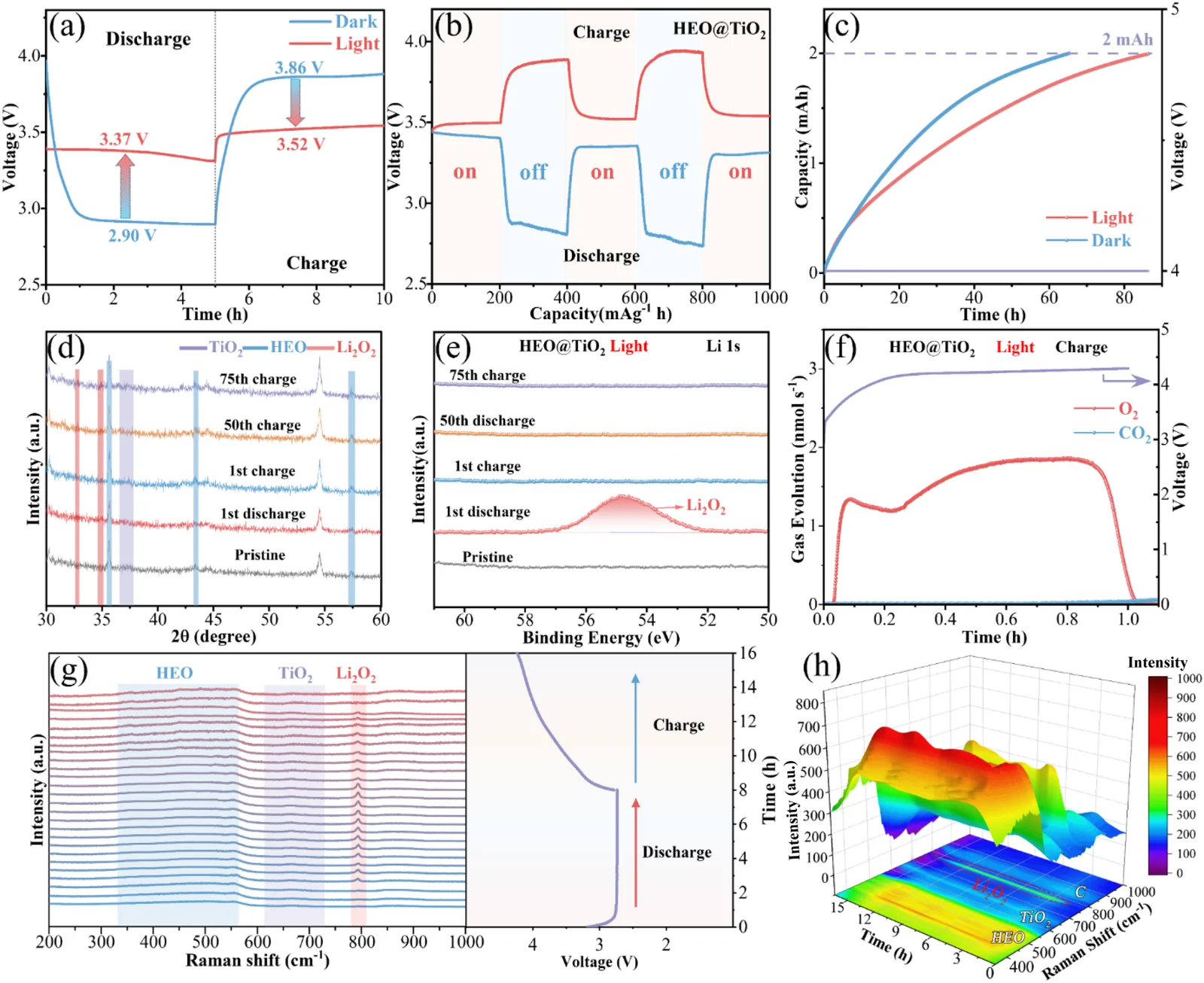
Electrochemical performance and *in*/*ex situ* characterization under VIS illumination. (a) First-cycle overpotential (200 mA g^−1^, limited capacity: 1000 mA h g^−1^) for HEO@TiO_2_ under light and dark conditions. (b) Photoresponse curve under intermittent illumination. (c) Constant-voltage charging rate under limited capacity. (d) XRD patterns and (e) XPS spectra of the HEO@TiO_2_ electrode at different charge–discharge stages under illumination. (f) DEMS spectra during the charging process of the illuminated HEO@TiO_2_ electrode. (g) and (h) *In situ* Raman spectra collected over 16 h of cycling and corresponding time-intensity contour maps.

Intermittent charge–discharge cycling tests with xenon lamp switching at 1 h intervals (Fig. 7b) demonstrated that the battery overpotential stabilized at approximately 0.1 V during illumination phases, rapidly increased to 0.9 V upon light cessation, and quickly decreased back to approximately 0.2 V upon light restoration. This reversible modulation unequivocally confirms the efficacy of the multi-channel photo-assisted mechanism in suppressing LOB polarization. Rate capability evaluations further validated the effect (Fig. S24). Even at a high current density of 1000 mA g^−1^ (with the capacity still limited to 1000 mA h g^−1^), illumination significantly suppressed polarization, maintaining the total overpotential stably below 0.7 V. The aforementioned polarization reduction effect directly manifests in the charging rate. As shown in Fig. 7c, during constant-voltage recharging at 4 V with a limited capacity of 2.0 mA h, the charging curve under illumination exhibited a significantly more stable slope. The charging time was reduced by approximately 21.1 h compared to that under dark conditions, representing a nearly 22.4% improvement in the charging rate. This underscores the substantial enhancement in charging efficiency afforded by reduced polarization, offering valuable implications for fast-charging battery technologies.

Furthermore, CV measurements in the voltage range of 2.0–4.5 V (Fig. S25) revealed that both the reduction and oxidation peak currents of the LOB were significantly higher under illumination compared to dark conditions. Integration and comparison of the anodic and cathodic peak areas further confirmed that the photo-assisted effect of the HEO@TiO_2_ composite substantially enhanced the bifunctional catalytic activity. EIS results (Fig. S26) demonstrated that the *R*_ct_ under illumination was only 42.8 Ω, considerably lower than the 78.3 Ω observed in the dark. This indicates that photo-assistance effectively mitigates the impedance caused by Li_2_CO_3_ byproducts during the charging process, leading to significantly optimized OER kinetics.

To assess potential thermal contributions from prolonged xenon lamp irradiation and evaluate material stability, long-term cycling tests (150 h, Fig. S27) were conducted under dark and illuminated conditions (200 mA g^−1^, capacity limited to 200 mA h g^−1^). In the dark, the overpotential remains stable at ∼0.9 V. Under illumination, the charge plateau increases only modestly (∼0.1 V relative to the initial cycle), whereas the discharge plateau declines by ∼0.5 V. This discharge plateau decay is primarily attributed to the progressive accumulation of the side product Li_2_CO_3_, whose photo-assisted decomposition efficiency is considerably lower than that of Li_2_O_2_, thereby obstructing O_2_ diffusion pathways. In contrast, during charging, photogenerated electrons can penetrate the side-product layer more effectively to facilitate Li_2_O_2_ decomposition, resulting in a relatively minor impact on the OER process. Despite these constraints, the illuminated battery sustains a relatively low overpotential of 0.7 V, markedly superior to the 0.9 V in the dark. This demonstrates that photo-assistance with HEO@TiO_2_ significantly enhances metal–oxygen electron transfer efficiency, improves reaction reversibility, and effectively alleviates polarization.

Overall, HEO@TiO_2_ exhibits excellent photo-responsive properties and charge–discharge reversibility, providing a theoretical paradigm for developing high-performance photo-assisted LOBs.

### 2.9. *In situ*/*ex situ* reaction characterization of the photo-assisted HEO@TiO_2_ cathode electrode

Fig. 7d presents the *ex situ* XRD patterns of the HEO@TiO_2_ electrode after discharge–charge cycling under illumination. Unlike in the dark, no crystalline Li_2_O_2_ diffraction features (*e.g.*, (100) or (101) reflections) emerge after the initial full discharge. Critically, these peaks remain absent through 75 cycles, confirming that illumination suppresses crystalline Li_2_O_2_ formation and significantly enhances electrode reversibility.

To identify discharge products under photo-assisted conditions, XPS analysis was performed. The Li 1s spectrum (Fig. 7e) exhibits a dominant peak at 55.4 eV (Li_2_O_2_) after discharge, while the Li_2_CO_3_ signal (56.4 eV) is significantly attenuated. This confirms that illumination promotes amorphous Li_2_O_2_ as the primary discharge product, accelerating kinetics and inhibiting parasitic Li_2_CO_3_ formation.

To evaluate whether illumination promotes the decomposition of amorphous Li_2_O_2_ during charging, we employed DEMS. Due to the light-impermeable design of the DEMS cell, *in situ* illumination during cycling was infeasible. Instead, a near-*in situ* approach was adopted: first, the electrode was discharged at 0.3 mA for 1 h under illumination in a glass vial and then rapidly transferred to the DEMS cell for charging analysis. As shown in Fig. 7f, charging efficiency significantly increased, with the e^−^/O_2_ ratio approaching the theoretical value of 2.02. Critically, CO_2_ evolution remained negligible, indicating near-complete suppression of Li_2_CO_3_ byproducts. These results indicate that the illumination applied during the preceding discharge process promoted the formation of amorphous, film-like Li_2_O_2_ discharge products, which exhibited significantly improved degradability and reversibility during the subsequent charging process. This is fully consistent with the surface growth mechanism proposed in this paper and the suppression of the formation of the byproduct Li_2_CO_3_ under illumination (Fig. 7e, 8d and e). Furthermore, to assess reaction reversibility, we performed *in situ* Raman spectroscopy on the HEO@TiO_2_ cathode during prolonged cycling (16 h total: 8 h discharge, 8 h charge) at a current density of 600 mA g^−1^, acquiring spectra every 30 min. As shown in Fig. 7g and h, the characteristic Raman signal of Li_2_O_2_ progressively intensified from baseline levels during discharge, reaching its maximum intensity after 8 h. Subsequently, this signal gradually diminished during the charging phase and nearly vanished by the end of charging,^67^ providing strong evidence for the excellent charge–discharge reversibility of the electrode under illumination. Notably, the characteristic Raman peaks of HEO and TiO_2_ remained stable throughout the entire process, further confirming the outstanding electrochemical structural stability of this heterostructured material.

**Fig. 8:**
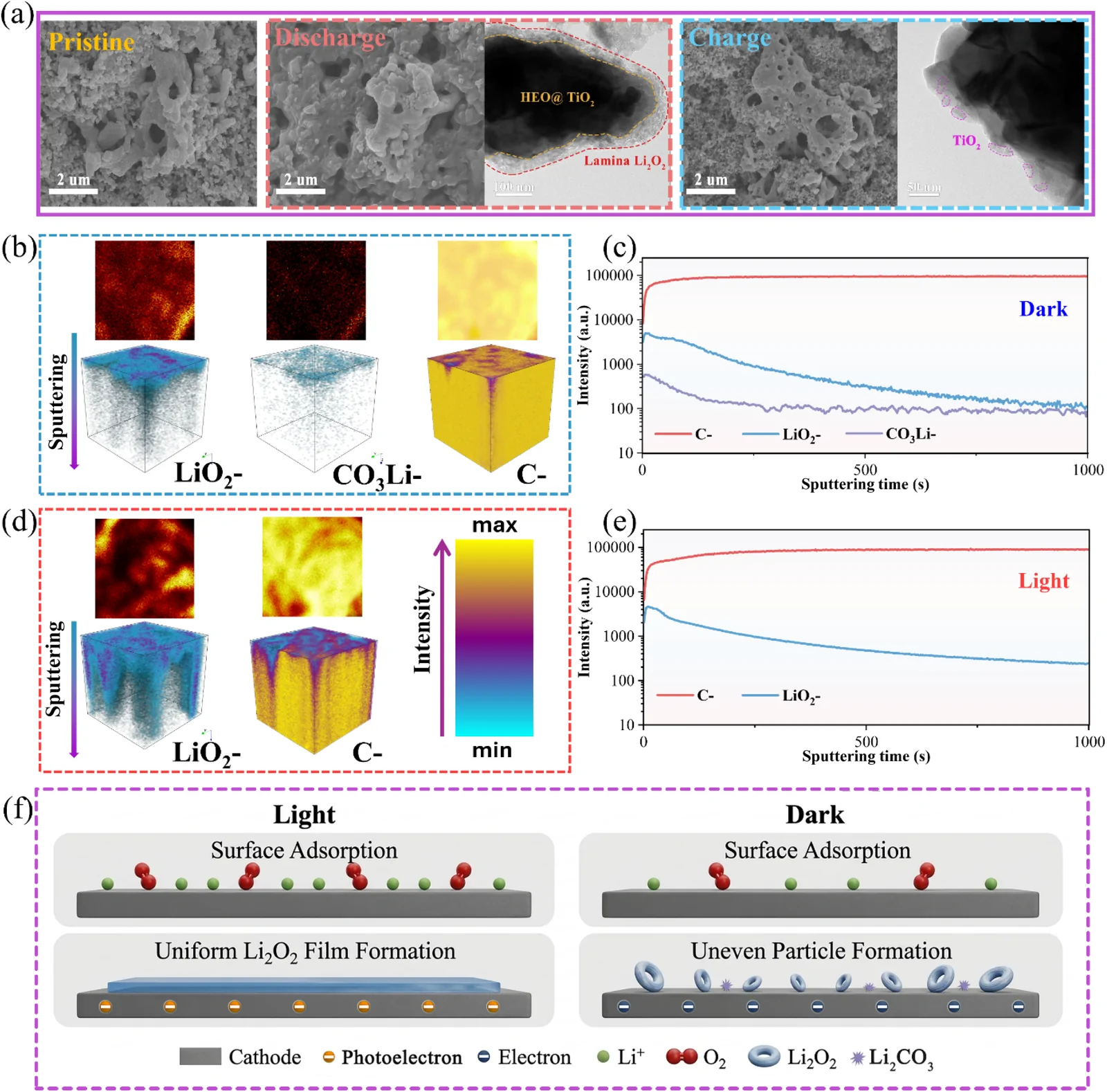
Analysis of the intermediate Li_2_O_2_ during the reaction. (a) SEM and TEM images of the HEO@TiO_2_ electrode under illumination: pristine state, fully discharged state and fully charged state. TOF-SIMS depth profiling distribution maps and corresponding 3D rendered images for (b) and (c) LiO_2_^−^, C^−^, and CO_3_Li^−^ signals under dark conditions and (d) and (e) under illumination, indicating the spatial distribution of the electrolyte/carbon matrix, discharge product (Li_2_O_2_), and byproduct (Li_2_CO_3_), respectively. (f) Proposed formation pathways of discharge products at the HEO@TiO_2_ cathode with and without illumination.

*Ex situ* SEM and TEM imaging visually captures the discharge/charge product evolution of the electrode surface under illumination (Fig. 8a). Following photo-assisted full discharge, the Li_2_O_2_ morphology shifts from the conventional toroidal structures to a continuous film-like deposit, ultimately forming a uniform thin film at the end of discharge. Similarly, in the SAED diffraction pattern of the discharged electrode in Fig. S31, the signals of Li_2_O_2_ and catalyst HEO@TiO_2_ were clearly detected. However, HRTEM revealed that Li_2_O_2_ exists in an amorphous state without obvious lattice fringes, which is consistent with the absence of Li_2_O_2_ signal peaks in the previous XRD pattern of the discharged electrode (Fig. 7a). This transition suggests that illumination accelerates reaction kinetics, modifies the Li^+^ concentration gradient within the electrolyte, and promotes rapid surface-mediated growth of Li_2_O_2_. Although precise film thickness quantification is challenging, the electrode surface morphology almost recovers after recharge. Lattice-resolved images (Fig. S28) demonstrate that the (211) plane of HEO and the (110) plane of TiO_2_ retain excellent lattice matching post-charging, indicating that cycling does not compromise the heterointerface integrity. Even after 75 cycles, the electrode morphology remains virtually identical to the initial state (Fig. S29), highlighting exceptional long-term structural stability and reaffirming high reversibility. To gain deeper insight into the differences in discharge product distribution under dark and illumination conditions and their impact on battery performance, we performed depth profiling using time-of-flight secondary ion mass spectrometry (TOF-SIMS). Under dark conditions, signals for LiO_2_^−^ (originating from Li_2_O_2_), CO_3_Li^−^ (originating from Li_2_CO_3_), and C^−^ (originating from the electrolyte/carbon substrate) were simultaneously detected as sputtering depth increased. These products were primarily enriched at the electrode/electrolyte interface, consistent with a typical solution-mediated growth mechanism (Fig. 8b and c).

In contrast, under illuminated conditions, only LiO_2_^−^ and C^−^ signals were observed, and their distribution was more uniform (Fig. 8d and e), highly consistent with the surface growth characteristics of amorphous film-like Li_2_O_2_. This suggests that illumination significantly increases nucleation site density, promoting a shift from solution growth to surface growth of the discharge product and effectively suppressing parasitic reactions. Based on the above experimental results, Fig. 8f provides a more intuitive illustration of the formation mechanism of Li_2_O_2_ under dark and illuminated conditions. During discharge, compared to the dark conditions, the HEO@TiO_2_ cathode under illumination exhibits a favourable surface growth mechanism for O_2_ adsorption and provides abundant nucleation sites for the deposition of discharge products, ultimately forming film-like products. This stands in stark contrast to the ring-shaped Li_2_O_2_ and small amounts of Li_2_CO_3_ byproducts observed in the dark. The amorphous film-like Li_2_O_2_ structure formed under illumination is more conducive to electron conduction and efficient decomposition during the OER. Through a built-in electric field with a reverse-blocking layer and an M–O covalent bond synergistic enhancement mechanism, the photo-assisted effect enables efficient separation of photogenerated carriers. Compared to dark conditions, illumination significantly enhances reaction kinetics, resulting in lower charge–discharge polarization, higher energy efficiency, faster charging rates, and superior cycling reversibility.

## 3 Conclusion

In summary, this study successfully constructed a light-responsive HEO@TiO_2_ heterostructure. At the heterointerface, the Ti–O bonds are significantly weakened, concurrently enhancing the covalency of adjacent metal M–O bonds. This effectively optimizes the hybridization degree between metal 3d and oxygen 2p orbitals, thereby substantially reducing the adsorption energy barriers for the LiO_2_ and O_2_ reaction intermediates. Furthermore, an S-scheme heterojunction with a reverse-blocking layer enriched with majority carriers forms at the interface between p-type HEO and n-type TiO_2_. The combination of the enhanced M–O covalency at the heterointerface and the built-in electric field associated with the reverse-blocking layer significantly improves the separation and migration efficiency of interfacial charges while promoting redox kinetics. Consequently, this synergistically enhances the reversible formation and decomposition capability of Li_2_O_2_, comprehensively improving the electrochemical performance of the LOB. Critically, the S-scheme heterojunction exhibits a distinct advantage for the spatial separation of photogenerated carriers under illumination, generating a larger redox potential difference. Consequently, the Li_2_CO_3_ discharge byproduct is virtually eliminated, the overpotential is lowered, and the charge/discharge rate is enhanced. Moreover, under light irradiation, the high-entropy material surface demonstrates a stronger electron enrichment effect, which further promotes the reduction reaction and significantly activates the high-entropy cocktail effect. This leads to a more pronounced increase in the discharge plateau voltage, fully leveraging the light-assisted enhancement effect. This composite cathode achieves an ultra-high specific capacity of 29 545 mA h g^−1^ in the dark and exhibits excellent stability over 867 cycles. Under light assistance, the discharge plateau voltage increases by 0.53 V, while the charge plateau voltage decreases by 0.32 V, yielding a remarkably high energy round-trip efficiency of 97.7% and an ultra-low charge/discharge overpotential of only 0.15 V. Additionally, under a 4 V constant-voltage limited-capacity charging mode, the charging rate is further increased by nearly 22.4%. This work demonstrates that the triple synergistic mechanism, comprising the high-entropy cocktail effect, interfacial M–O covalent bond enhancement, and S-scheme heterojunction anti-blocking layer, effectively drives efficient interfacial redox reactions. Importantly, this mechanism further enhances electrical performance under illumination, providing a novel and reliable interfacial engineering strategy and materials design concept for achieving high-performance LOBs and light-assisted enhanced catalysis.

## Author contributions

Bai Zheng: conceptualization, investigation, visualization, DFT simulations, writing – original draft and writing – review & editing. Qinghua Sun, Rourou Yi, Huiying Zhou, Wenju Xie, Fuli Xie: investigation. Jie Zhao, Yanhe Xiao, and Shuijin Lei: review and edited the manuscript as a whole in detail. Baochang Cheng: supervision, writing – review and editing, and finalizing the manuscript prior to submission.

## Conflicts of interest

There are no conflicts to declare.

## Supplementary Material

SC-017-D6SC05480C-s001

## Data Availability

The data that support the findings of this study are available in the supplementary information (SI) of this article. Supplementary information: experimental section, SEM, SAED, DFT calculation, XPS, Tauc plots, Mott–Schottky curves, theoretical work function, dielectric constant, charge–discharge curves, overpotentials, cycling performance, DRT spectra, XRD and XPS spectra at different charge/discharge stages, DEMS spectra, transient photovoltage, rate capability under illumination, CV and EIS curves under light and dark conditions, cycling stability, cycling stability, HRTEM images in the fully charged state and after 150 h of cycling under light illumination, element content table, and supporting data for this article. See DOI: https://doi.org/10.1039/d6sc05480c.
