# Supplementary material for: Reverse barrier layer and interfacial M–O bond reinforcement in an S-scheme high-entropy oxide heterojunction for efficient photo-assisted lithium–oxygen batteries
Source: Chem Sci. 2026 Aug 4;17(38):18379–95. doi: 10.1039/d6sc05480c (PMC13463299; doi:10.1039/d6sc05480c)
Supplement: Supplementary file 1 [file SC-017-D6SC05480C-s001.pdf]

Supplementary Information (SI) for Chemical Science.
This journal is © The Royal Society of Chemistry 2026

# Supporting Information

## Reverse barrier layer and interfacial M-O bond reinforcement in an S-scheme high-entropy oxide heterojunction for efficient photo-assisted lithium-oxygen batteries

Bai Zheng,[a] Qinghua Sun,[a] Rourou Yi,[a] Huiying Zhou,*[b] Wenju Xie,[c] Fuli Xie,[a] Jie Zhao,*[a] Yanhe Xiao,[a] Shuijin Lei[a] and Baochang Cheng*[a]

[a.] *School of Physics and Materials Science, Nanchang University, Jiangxi 330031, P. R. China*

[b] *School of Electronic Information and Physics, Central South University of Forestry and Technology, Hunan 410004, P. R. China*

[c.] *College of Ecology and Resources Engineering, Fujian Provincial Key Laboratory of Eco-Industrial Green Technology, Wuyi University, Fujian 354300, P. R. China*

**Email:** T20070600@csuft.edu.cn; jzhao@ncu.edu.cn; bcheng@vip.sina.com

# 1. Experimental Section

## 1.1. Main Materials

All chemical reagents were used as purchased, without further treatment. The materials include: $Fe(NO_3)_3{\cdot}9H_2O$, ≥99%, $Co(NO_3)_2{\cdot}6H_2O$, ≥99%, $Ni(NO_3)_2{\cdot}6H_2O$, ≥99%, $Mn(NO_3)_2{\cdot}4H_2O$,≥99%, $Zn(NO_3)_2{\cdot}6H_2O$, ≥99%, $Cr(NO_3)_3{\cdot}9H_2O$, ≥99%, Titanium butoxide (TBOT) ≥99%, Acetylacetone (Hacac, ≥99%), Polyvinylidene fluoride (PVDF), N-Methyl-2-pyrrolidone (NMP), Lithium metal foil (Li, 99.9%), Lithium bis(trifluoromethanesulfonyl)imide (LiTFSI, anhydrous, 99.5%), Tetraethylene glycoldimethyl ether (TEGDME, 99.5%) were purchased from Shanghai Aladdin Biochemical Technology Co., Ltd. Ethanol (EtOH, $CH_3CH_2OH$, ≥99.5%) was obtained from China National Pharmaceutical Group Chemical Reagents Co., Ltd. The separator (GF/D, Whatman), mesh-type button battery components (CR2032), conductive carbon black (SuperP), gas diffusion layers, and hydrophobic carbon paper were purchased from Canrd (Keluo De). Ultra-pure water (18.2 MΩ·cm) was prepared using the Direct-Q®8 UV-R ultra-pure water system from Merk Millipore.

## 1.2. Material Synthesis

### 1.2.1. Synthesis of High Entropy Spinel Oxide $FeCoNiMnZnCrO_x$ (HEO)

To prepare the high entropy oxide (HEO), 1 mmol of $Fe(NO_3)_3{\cdot}9H_2O$, 1 mmol of $Co(NO_3)_2{\cdot}6H_2O$, 1mmol of $Ni(NO_3)_2{\cdot}6H_2O$, 1mmol of $Mn(NO_3)_2{\cdot}4H_2O$, 1 mmol of $Zn(NO_3)_2{\cdot}6H_2O$, and 1 mmol of $Cr(NO_3)_3{\cdot}9H_2O$ were added to 10 mL of deionized water, followed by the addition of 0.6 g of glycine. The mixture was stirred thoroughly and then heated in an oven at 80 °C for 4 h to form a gel-like substance. The gel was then transferred to a muffle furnace and heated at a rate of 5 °C/min to 900 °C, where it was kept for 1 h. After natural cooling, a fluffy dendritic product was obtained. The product was ground to obtain the HEO powder.

### 1.2.2. Preparation of Low-Size Nano-Spherical $TiO_2$ Particles

200 μL of acetylacetone was added to 40 mL of anhydrous ethanol as a chelating agent to prevent $TiO_2$ particle agglomeration. The solution was magnetically stirred for 10 min to form a homogeneous pale-yellow solution. 0.5 mL of titanium butoxide solution was slowly added to the yellow solution under ultrasound, and the mixture was stirred for 30 min. The solution was then transferred to a 100 mL polytetrafluoroethylene (PTFE) high-pressure reaction vessel and heated in an oven at 180 °C for 12 h. After the reaction, the white precipitate was collected and washed by filtering three times with deionized water and once with anhydrous ethanol. The product was dried in a vacuum drying oven at 60 °C for 24 h and then ground for collection.

**1.2.3. Synthesis of HEO@$TiO_2$ Heterojunction by Modifying $TiO_2$ Particles on the Surface of HEO**

0.1 g of HEO powder was added to 30 mL of pre-prepared 0.1 M NaOH solution and heated at 70 °C with stirring for 1 h for hydroxylation of the HEO surface. The resulting hydroxylated solution was filtered through deionized water three times and anhydrous ethanol once until neutral. The black precipitate was collected and dried in a vacuum drying oven at 60 °C for 24 h to obtain hydroxylated HEO powder. To prepare the HEO@$TiO_2$ heterojunction, 0.2 g of hydroxylated HEO powder, 0.4 g of urea, and 0.2 g of hexadecyltrimethylammonium bromide were added to 50 mL of ethanol solution and stirred for 30 min to disperse the mixture evenly. 1 mL of acetylacetone was added as a chelating agent, and the mixture was stirred for an additional 10 min. Then, 0.5 mL of titanium butoxide solution was slowly added under ultrasound. After 20 min of ultrasonication, the solution was transferred to a 100 mL PTFE high-pressure reaction vessel and heated in an oven at 180 °C for 12 h. After the reaction, the gray-black precipitate was collected, washed by centrifuging three times with deionized water and once with a hydrous ethanol, and dried in a vacuum drying oven at 60 °C for 24 h. The final product, HEO@$TiO_2$ powder, was ground for collection.

**1.3. LOB Assembly**

**1.3.1. Electrode Preparation**

The catalyst samples, conductive agent (Super P), and binder (PVDF) were mixed in a 6:3:1 mass ratio, ground uniformly, and dispersed in a specified volume of N-methyl-2-pyrrolidone (NMP). The resulting mixture was sonicated for 4 h to obtain a slurry with suitable viscosity. The slurry was evenly sprayed onto pre-cut and pre-weighed carbon paper current collectors, followed by vacuum drying at 60 °C for 12 h. The cathode catalyst loading was approximately 0.3-0.6 mg $cm^{-2}$. Metallic lithium discs (Φ 14 mm) served as the anode, and glass fiber (Φ 19 mm, Whatman GF/D) was used as the separator. The electrolyte consisted of 1 mol $L^{-1}$ LiTFSI dissolved in tetraethylene glycol dimethyl ether (TEGDME).

### 1.3.2. Battery Assembly

All lithium-oxygen batteries used for testing were assembled in CR2032 mesh coin cell conFig.urations (including positive and negative electrode casings, spacers, and springs). The assembly was conducted in an argon-filled glove box ($H_2O$<0.1 ppm, $O_2$<0.1 ppm). The components were stacked in the following sequence: negative electrode casing, spring washer, spacer, lithium metal disc, separator, electrolyte, positive electrode, and porous positive electrode casing. The assembled components were sealed using a manual crimping machine under 50 MPa pressure for 10 s. After sealing, the cells were rested for 8 h to ensure thorough electrolyte infiltration before testing.

### 1.4. Material Characterization

The microstructure of the samples was examined using a field emission scanning electron microscope (FESEM, FEI Quanta 200FEG) and a transmission electron microscope (TEM, JEOL JEM 2100F) equipped with an energy dispersive spectrometer (EDS). Powder X-ray diffraction (XRD) analyses were conducted on a PANalytical Empyrean system with copper as the target (Cu $K_\alpha$, $\lambda$=1.5406 Å). Surface area and pore size distribution were assessed using the Brunauer-Emmett-Teller (BET) technique and Barrett-Joyner-Halenda (BJH) theory on a Micromeritics ASAP2460 instrument. Thermogravimetric analysis (TG) was performed on a Swiss Mettler TGA/DSC1 TG analyzer. X-ray photoelectron spectroscopy (XPS) was

performed using a Shimadzu (Kratos) Axis Supra+ spectrometer; XPS data were calibrated against the C 1s peak at 284.8 eV. Elemental composition of catalyst was determined via inductively coupled plasma (ICP) analysis using an EXPEC-6500 instrument. Photoluminescence (PL) spectra were adopted to investigate the influence of the full-space electric field on the photogenerated charge carrier transfer dynamics using a HORIBA FluoroMAX-4 spectrophotometer under excitation at 360 nm. The surface potential were monitored by the atomic force microscope (AFM, Bruker Dimension Icon XR) test system equipped with the Kelvin probe force microscopy (KPFM) module. The femtosecond transient absorption (fs-TA) measurements were performed under ambient conditions on a Helios pump-probe system (Ultrafast Systems LLC) in combination with an amplified femtosecond laser system (Coherent). The X-ray absorption near-edge structure (XANES) of Fe K-edge were monitored at beamline BL02B of Shanghai Synchrotron Radiation Facility using the transmission mode. Time-of-flight secondary ion mass spectrometry (TOF-SIMS) was tested by IONTOF 5, Münster, Germany.

**1.5. LOB Testing**

Battery performance was evaluated in a sealed system filled with oxygen. Cyclic voltammetry (CV) measurements were performed using a Chenhua CHI 760E and Autolab PGSTAT302N electrochemical workstation in the voltage range of 2.0-4.5 V (vs. $Li/Li^+$) at a scan rate of 0.1 mV $s^{-1}$. Electrochemical impedance spectroscopy (EIS) was carried out at open-circuit potential over a frequency range of 0.01–100 kHz with an amplitude of 5 mV. Constant-current charge-discharge performance was tested using a LAND CT 201A battery testing system in the voltage range of 2.2-4.5 V (vs. $Li/Li^+$). The cycling stability of the batteries was evaluated under various current densities and fixed cutoff capacities. Moreover, the discharge mid-potential and charge mid-potential were selected as the representative charge/discharge plateau criteria, and the resulting potential difference was taken as the overpotential test result.

Unless otherwise specified, current density and charge-discharge capacities were calculated based on the mass of the catalytic active material (catalyst) in the cathode.

The discharge-charge products were analyzed using ex-situ SEM, XRD, and XPS. For this, the cells were disassembled in the glove box, and the cycled electrodes were rinsed with TEGDME to remove residual electrolyte, followed by vacuum drying. The cleaned electrodes were sealed in laminated plastic packaging inside the glove box and then transferred for external analysis.

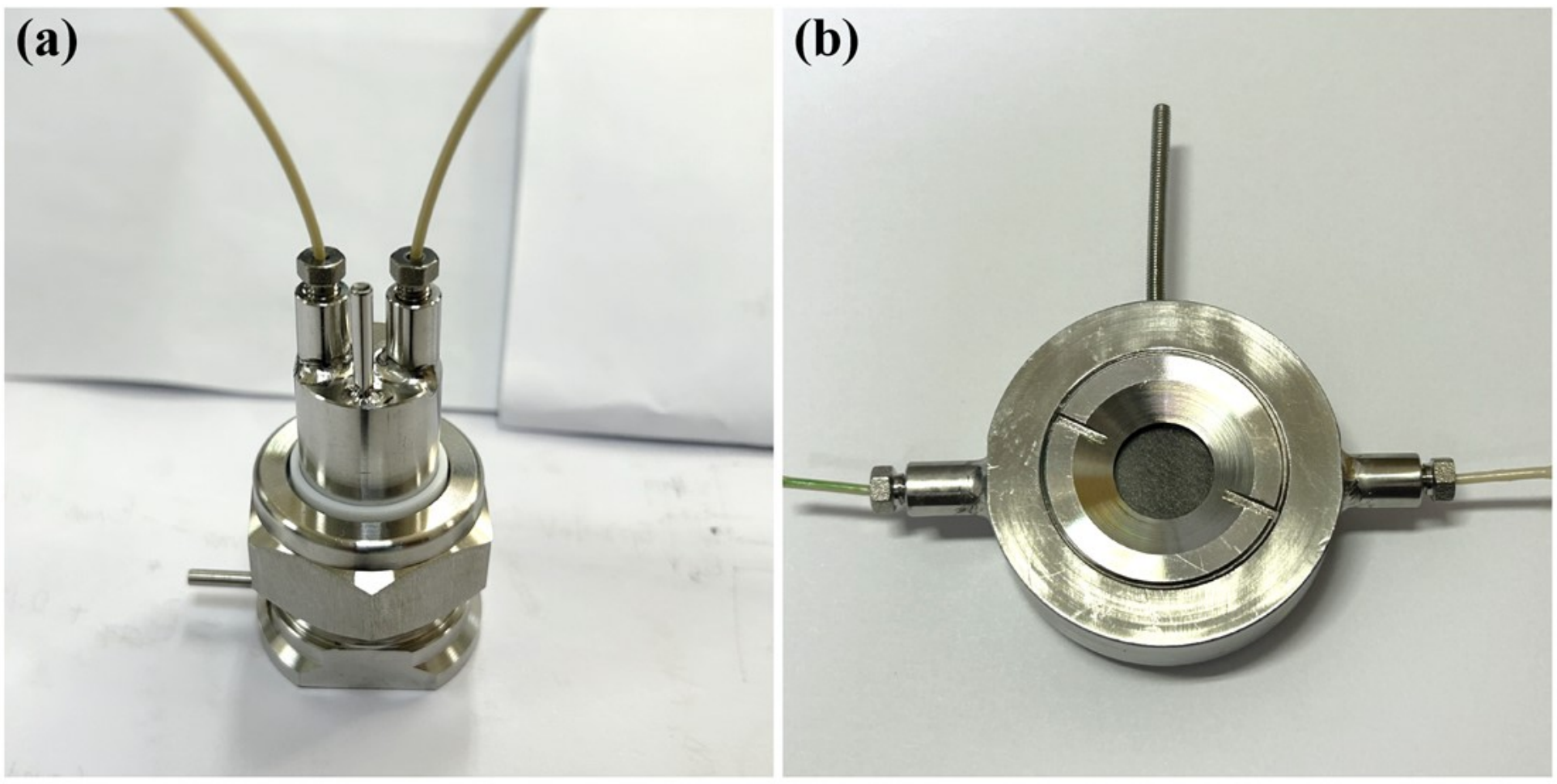


**Fig. S1.** (a) Swagelok cell for in situ differential electrochemical mass spectrometry; (b) In-situ Raman cell for LOB testing.

In-situ Raman and in-situ differential electrochemical mass spectrometry (DEMS) were employed to analyze discharge-charge products. In-situ Raman testing was conducted using a customized Raman cell coupled with a Raman spectrometer (InVia Reflex, Renishaw, UK) and a battery testing system. The in-situ Raman cell for lithium-oxygen battery testing is shown in Fig. S1b. Gas analysis during battery charge-discharge was conducted using an in-situ electrochemical mass spectrometer (DEMS, Shanghai Zero Leakage QAS 100 Li) with a custom Swagelok battery assembly (Fig. 2Sa), using a discharge gas flow rate of 3.09 $mL \cdot min^{-1}$ in a 40:1 (V/V) $Ar/O_2$ mixture during discharge and high-purity argon during charge, maintaining a constant current of 0.3 mA and a 1 h charge-discharge duration. To clearly

observe gas evolution during discharge and charge, the catalyst loading for DEMS was 10 times higher than that used for performance testing, with a current density of 400 mA $g^{-1}$. The Swagelok cell design is shown in Fig. 1a.

**1.6. Light-Assisted Battery Testing and Photoelectrochemical Measurements.**

A 2032-type positive electrode shell with an aperture diameter of 5 mm was used, with simulated sunlight source (AM1.5G, Beijing NBET Solar-500T) set at a fixed power of 100 mW $cm^{-2}$ to continuously illuminate the battery cathode. In a 0.2 M $Na_2SO_4$ solution (pH=7.0), a three-electrode system was established for cathodic photoelectrochemical reactions, employing a Pt foil counter electrode and an Ag/AgCl reference electrode.

**1.7. Theoretical Simulation Calculations**

Density Functional Theory (DFT) simulations of LOBs were carried out using the Vienna Ab initio Simulation Package (VASP). The exchange-correlation potential was described using the Perdew-Burke-Ernzerhof Generalized Gradient Approximation (GGA-PBE). The Projector Augmented-Wave (PAW) method was used to treat interactions between ionic cores and valence electrons. Kohn-Sham single-electron states were expanded with a plane-wave basis set with a kinetic energy cutoff of 450 eV. To study surface reaction mechanisms, a vacuum region of 15 Å was used in the vertical direction. Brillouin zone integration utilized the Monkhorst-Pack sampling method, with a grid density of 3×3×1 for optimization. During geometry optimization, the maximum force on atoms was relaxed below 0.02 eV/Å with total energy convergence at $1\times10^{-5}$ eV. Gibbs free energy of intermediates was calculated using the Nørskov method. Adsorption energy ($E_{ads}$) was defined as: $E_{ads}=E_{ad/sub}-E_{ad}-E_{sub}$, where $E_{ad/sub}$ is the energy of the occupied substrate, $E_{ad}$ is the energy of the adsorbate, and $E_{sub}$ is the energy of the substrate. Free energy changes (G) were calculated per step using: $G=E_{ads}+\Delta ZPE-T\Delta S$, where ΔZPE is the zero-point energy change, ΔS is the entropy change between adsorbate and substrate, and T is set at 298.15 K. ΔZPE was determined through vibrational frequency analysis. Additionally, for the +U correction applied to the transition metal oxide system, the U values for Fe, Co, Ni, Mn, Zn, Cr, Ti, and O were set to 4.0, 3.3, 6.4, 3.9, 0, 3.5, 0, and 0, respectively. The zero U values are attributed to the fully occupied spin channels, where no unpaired orbital electrons are present.

## 2. Figures

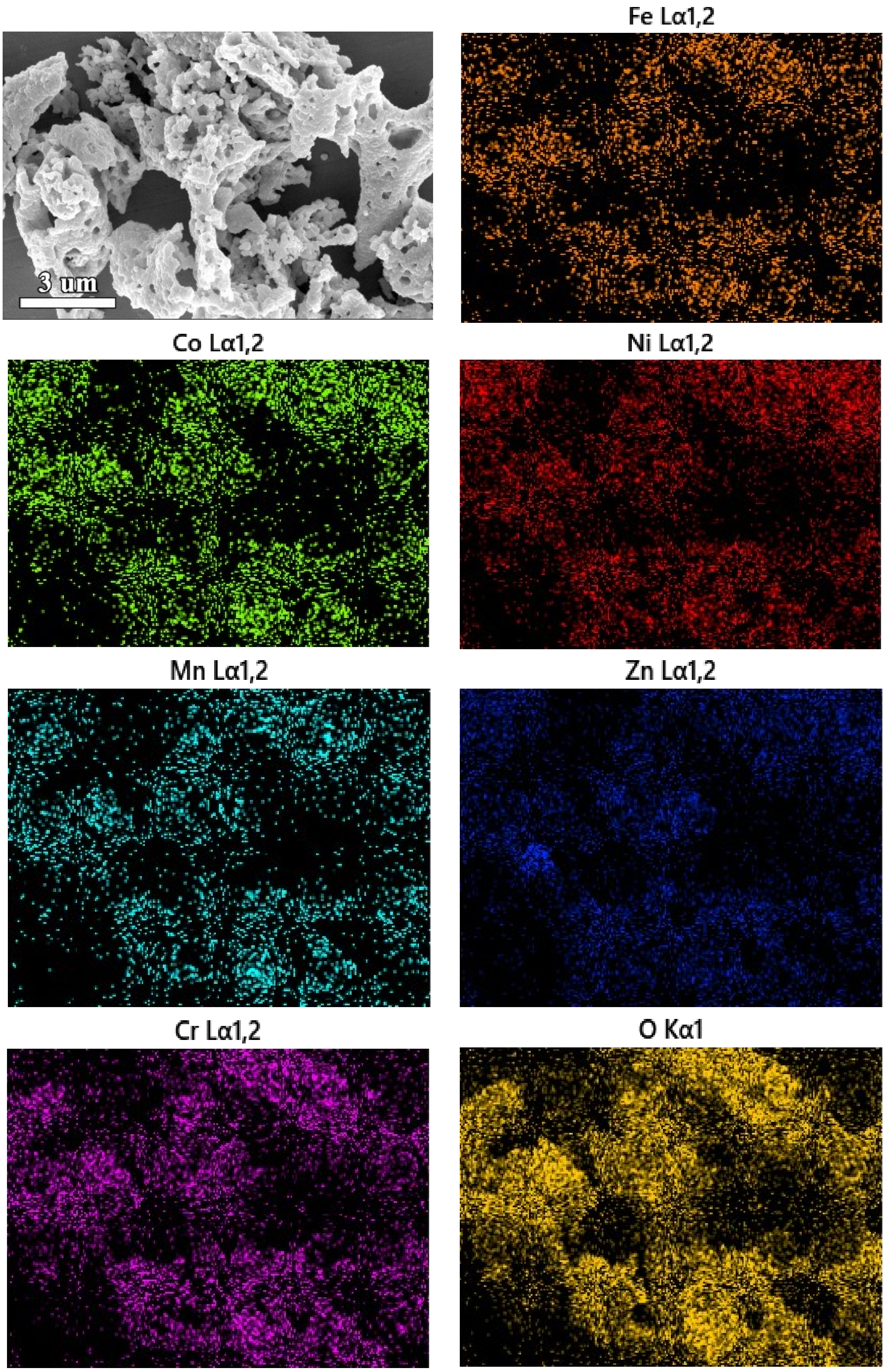


**Fig. S2.** FESEM images of HEO and corresponding mapping diagram of Fe, Co, Ni, Mn, Zn, Cr and O elements, respectively.

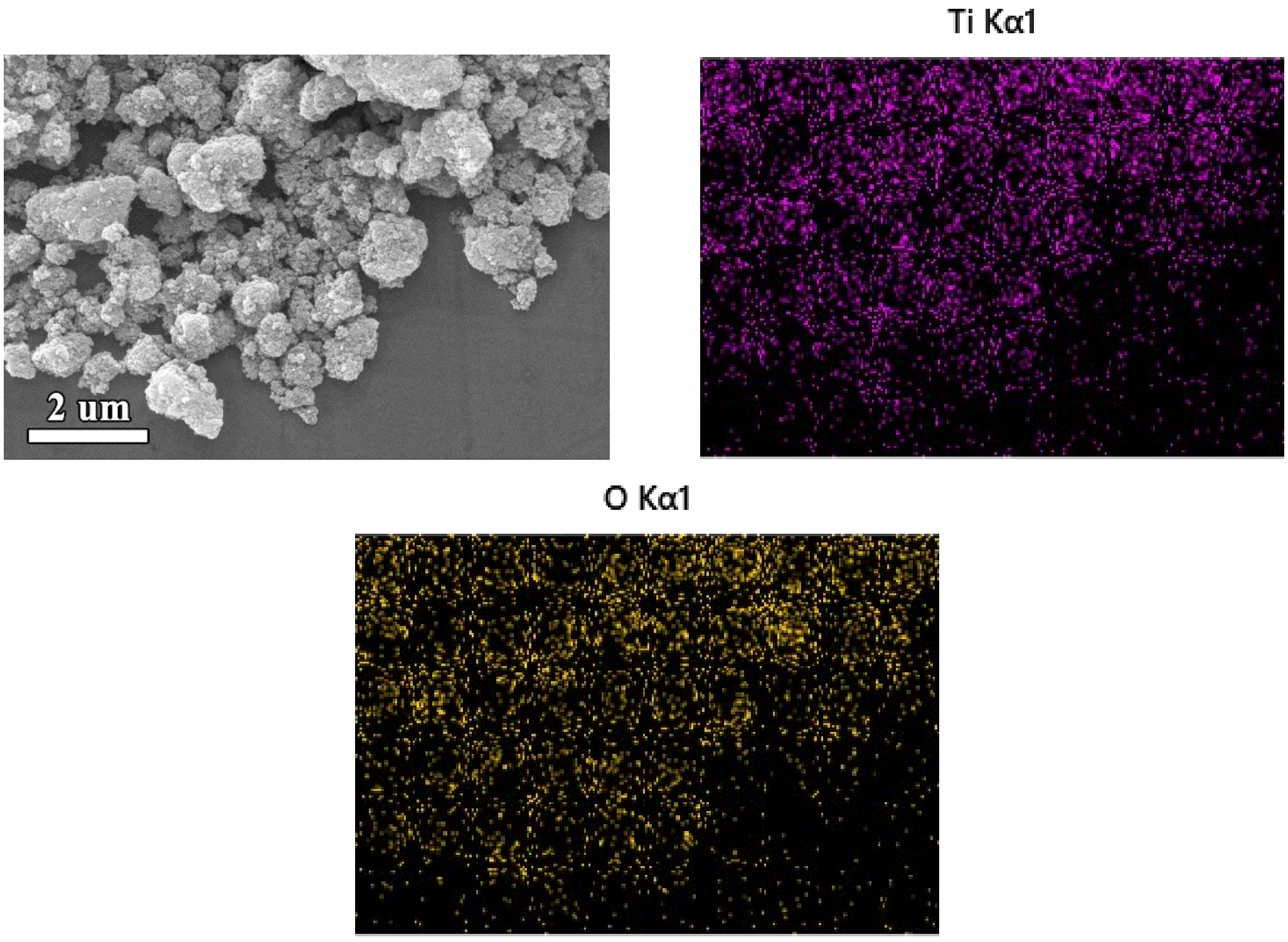


**Fig. S3.** FESEM images of $TiO_2$ and corresponding mapping diagram of Ti and O elements, respectively.

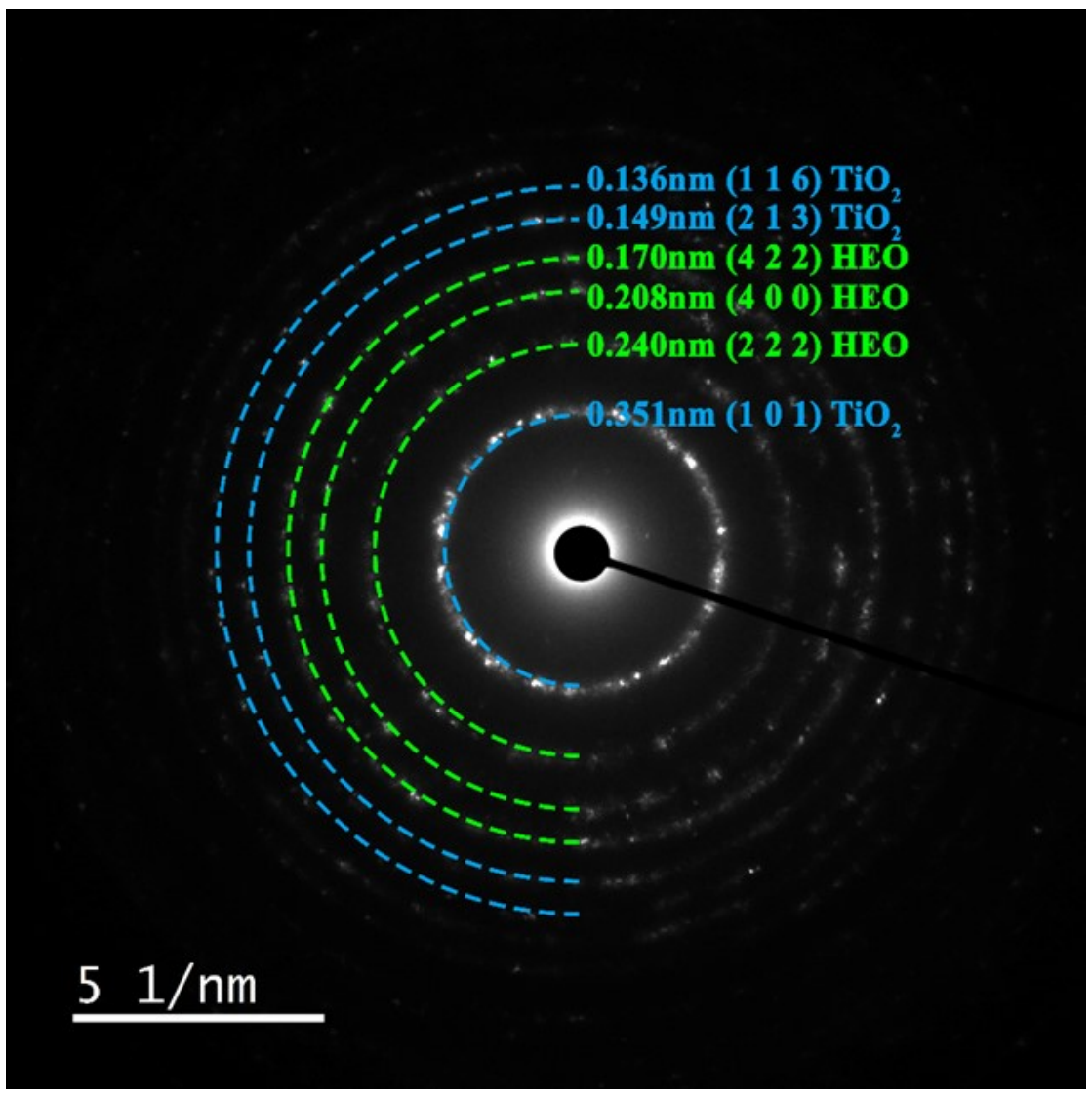


**Fig. S4.** SAED pattern of HEO@$TiO_2$.

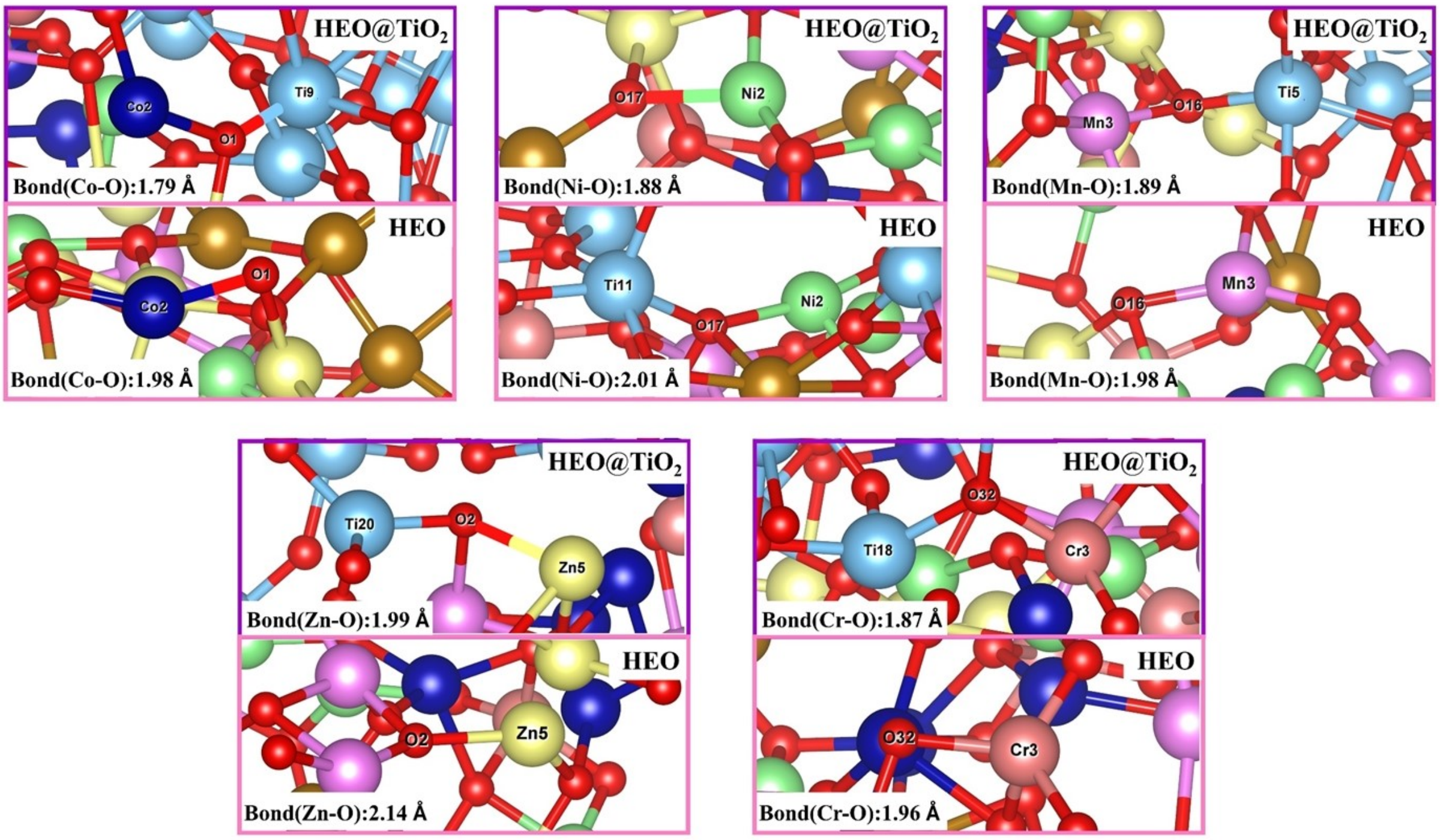


**Fig. S5.** Comparison of bond strengths before and after forming heterojunctions between Co-O, Ni-O, Mn-O, Zn-O, and Cr-O.

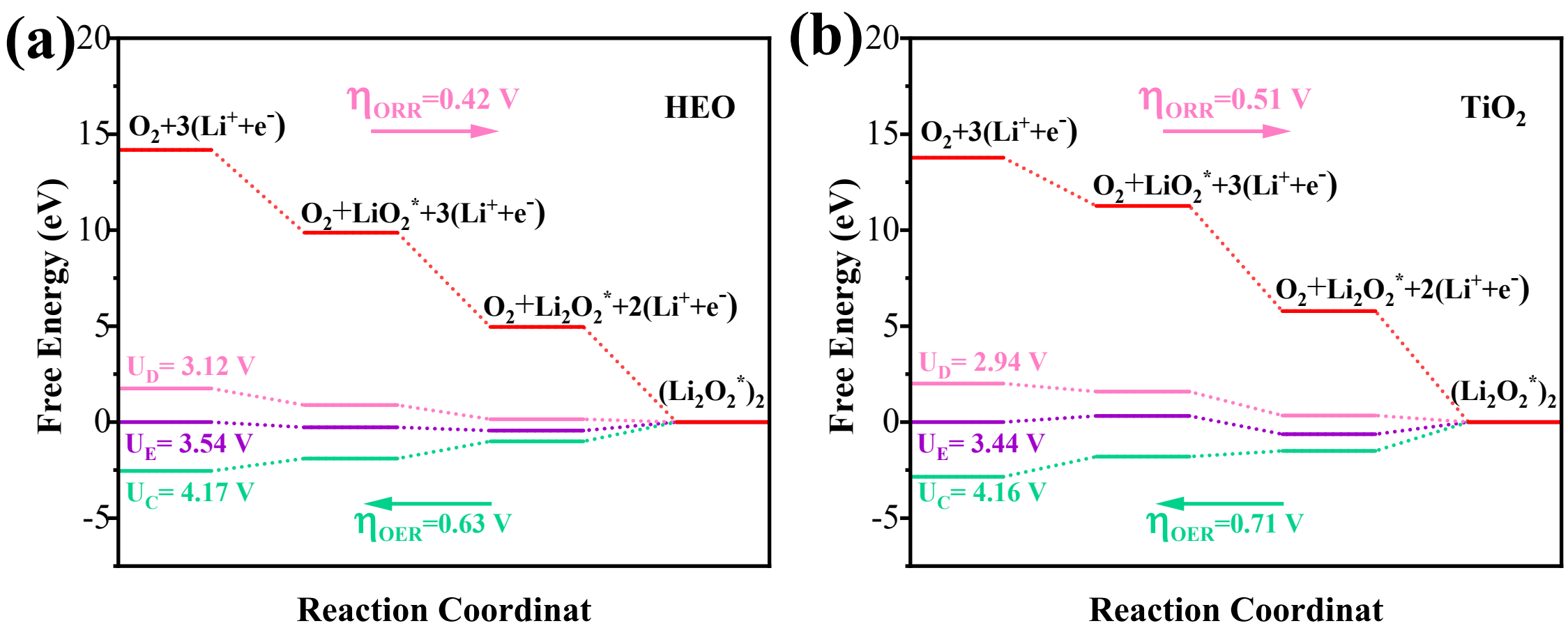


**Fig. S6.** Free energy diagram of the surface redox reaction for HEO and $TiO_2$.

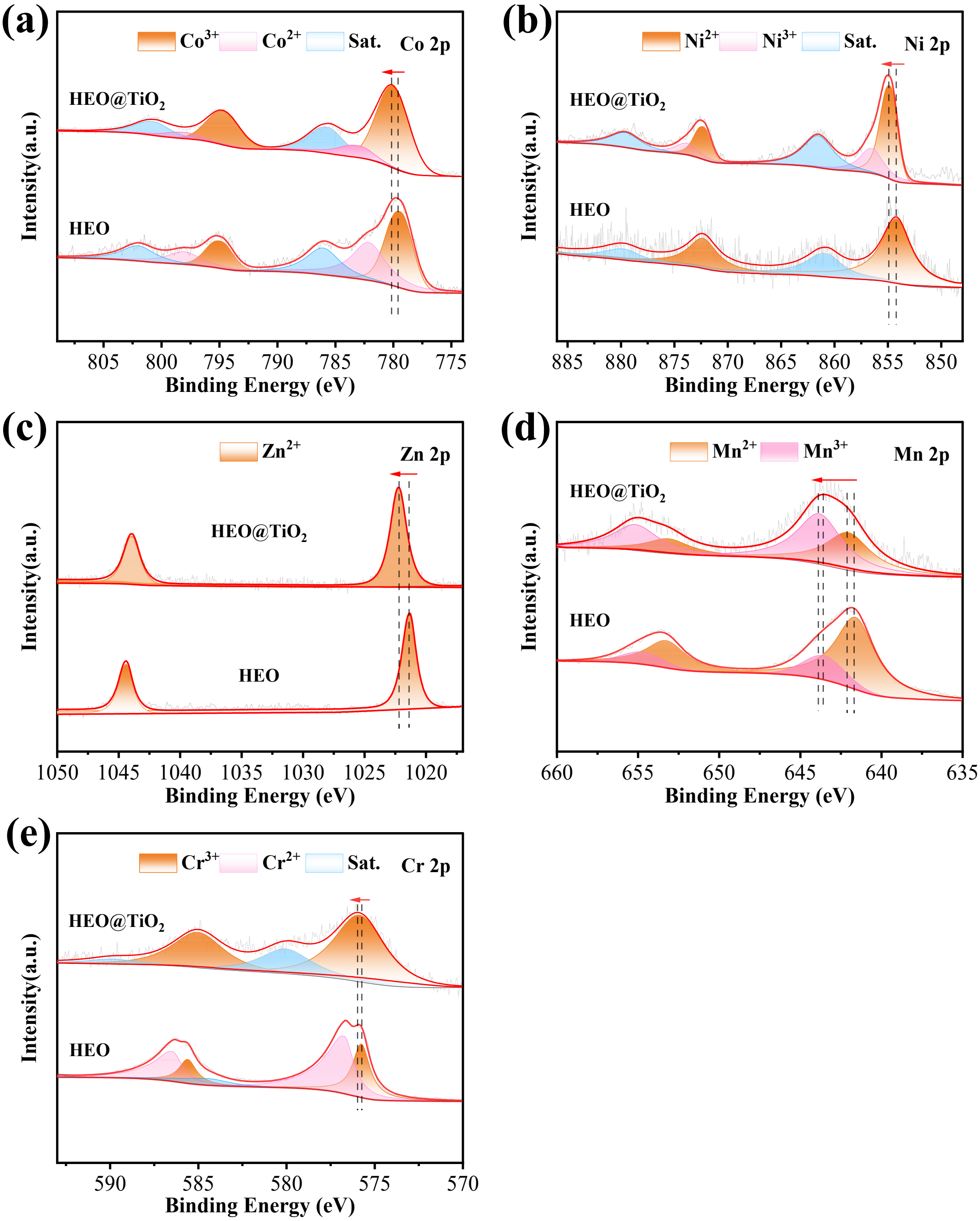


**Fig. S7.** Comparison of XPS spectra for (a) Co-2p, (b) Ni-2p, (c) Mn-2p, (d) Zn-2p, and (e) Cr-2p.

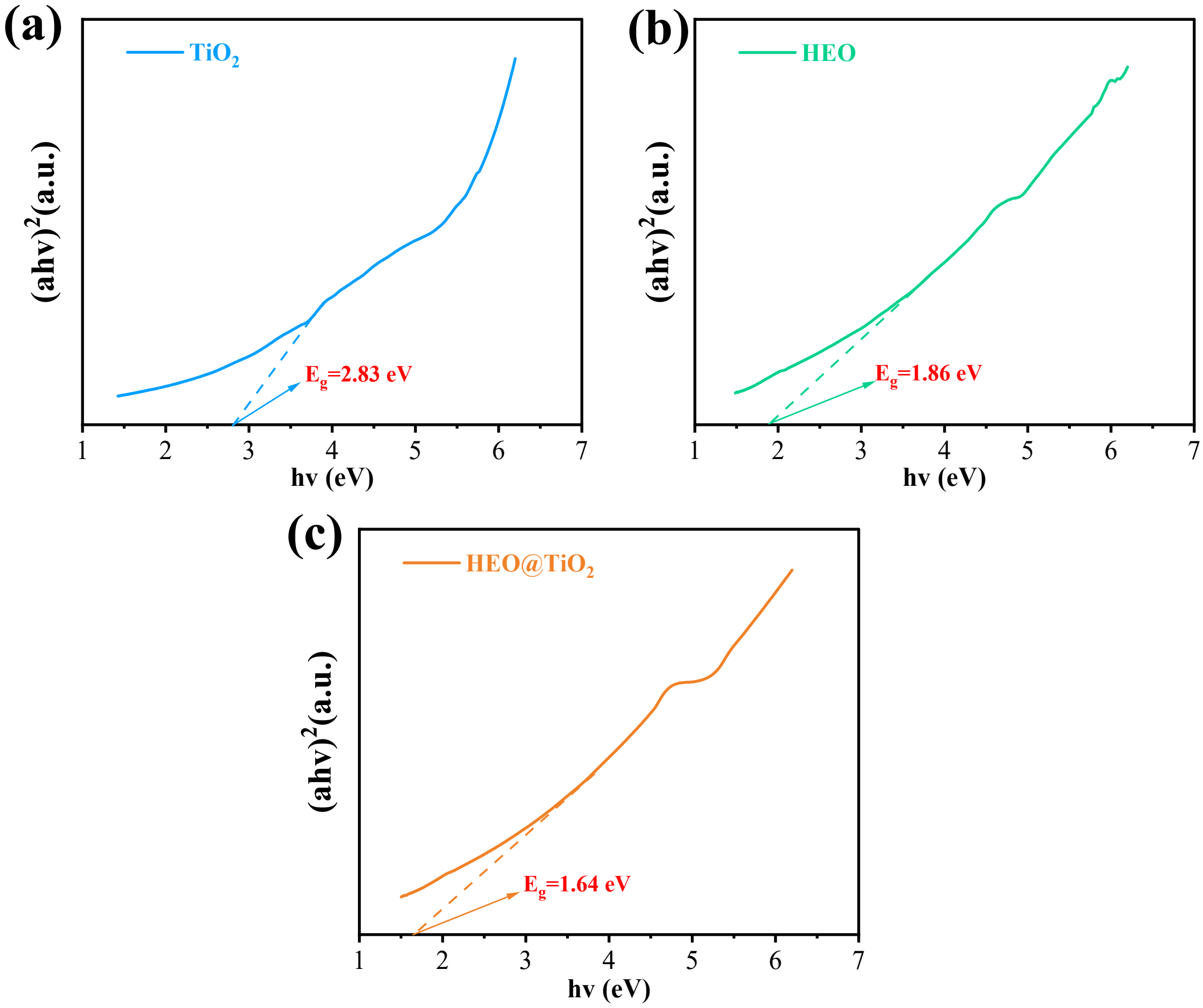


**Fig. S8**. Tauc plots for (a) $TiO_2$, (b) HEO, and (c) HEO@$TiO_2$.

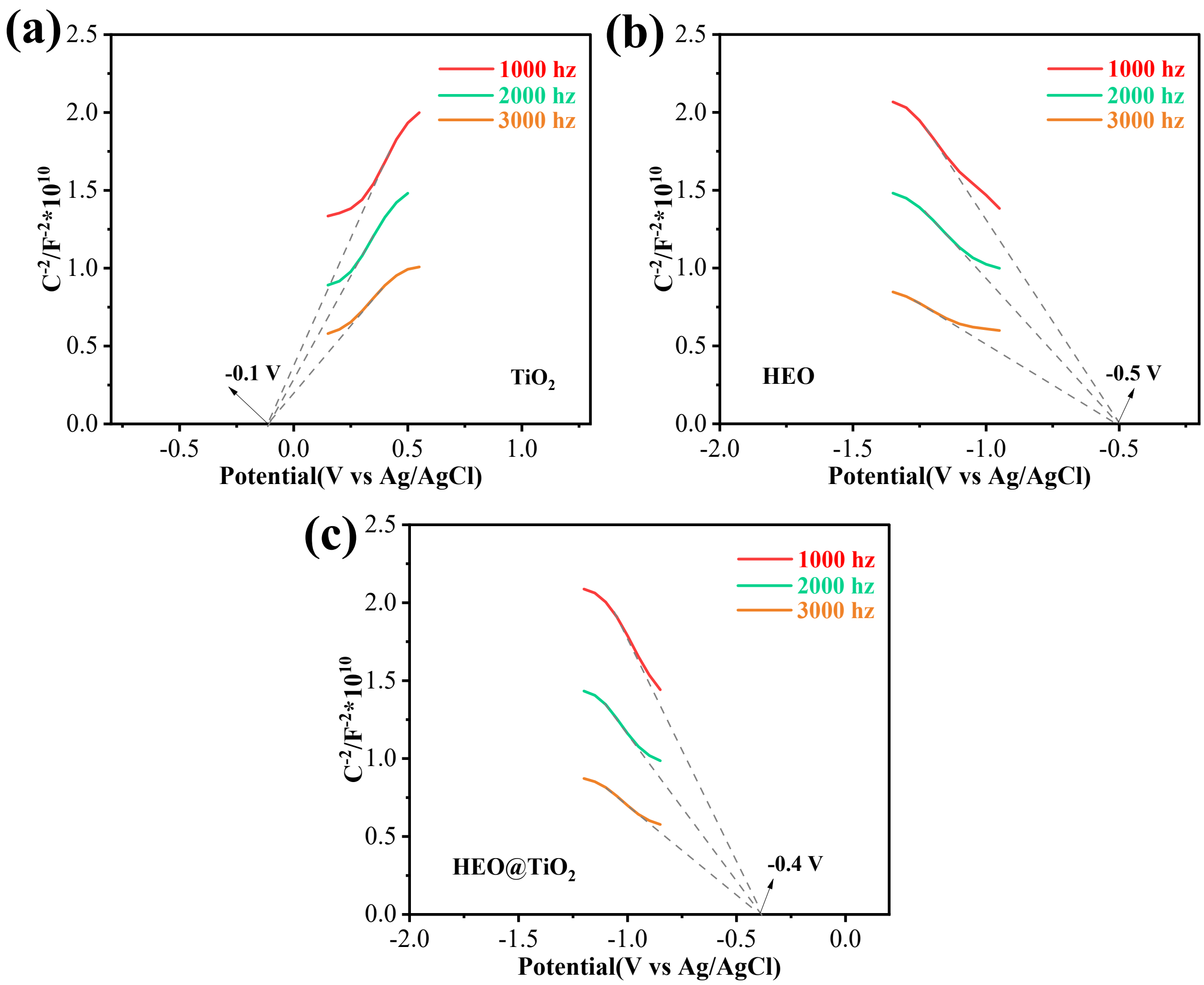


**Fig. S9.** Mott-Schottky curves for (a) $TiO_2$, (b) HEO, and (c) HEO@$TiO_2$.

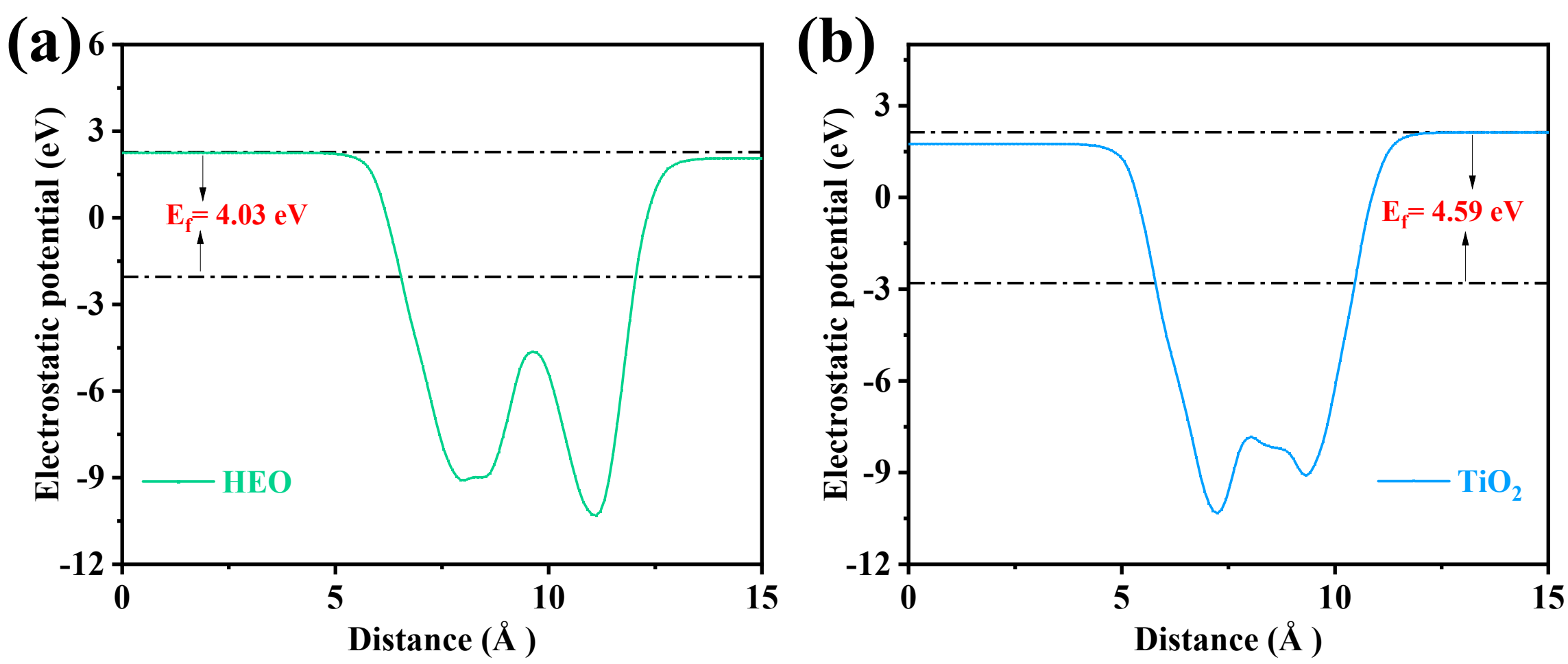


**Fig. S10.** (a) Theoretical work function of HEO and (b) $TiO_2$.

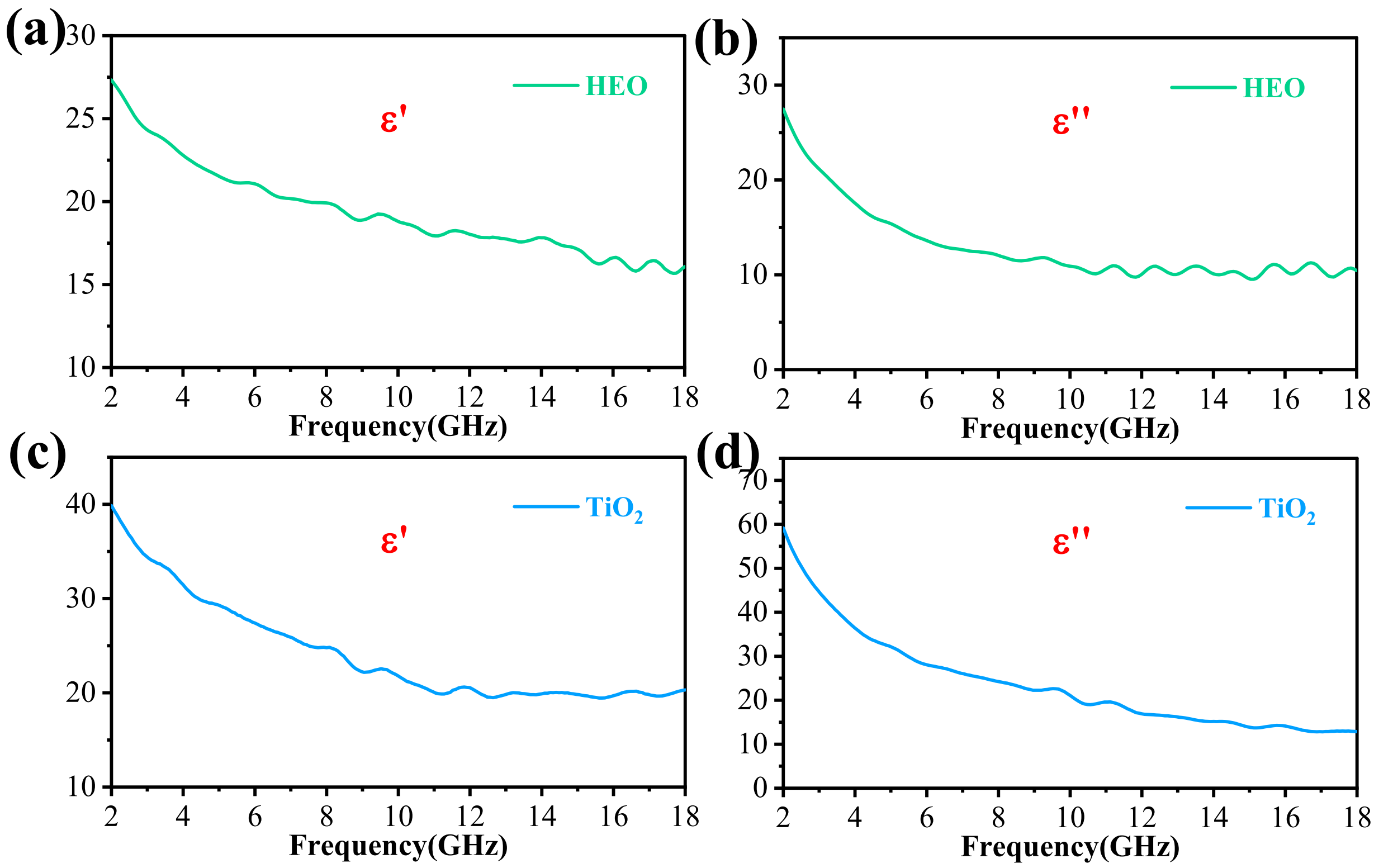


**Fig. S11.** Real part (ε') and imaginary part (ε'') of the dielectric constant for (a-b) HEO and (c-d) $TiO_2$.

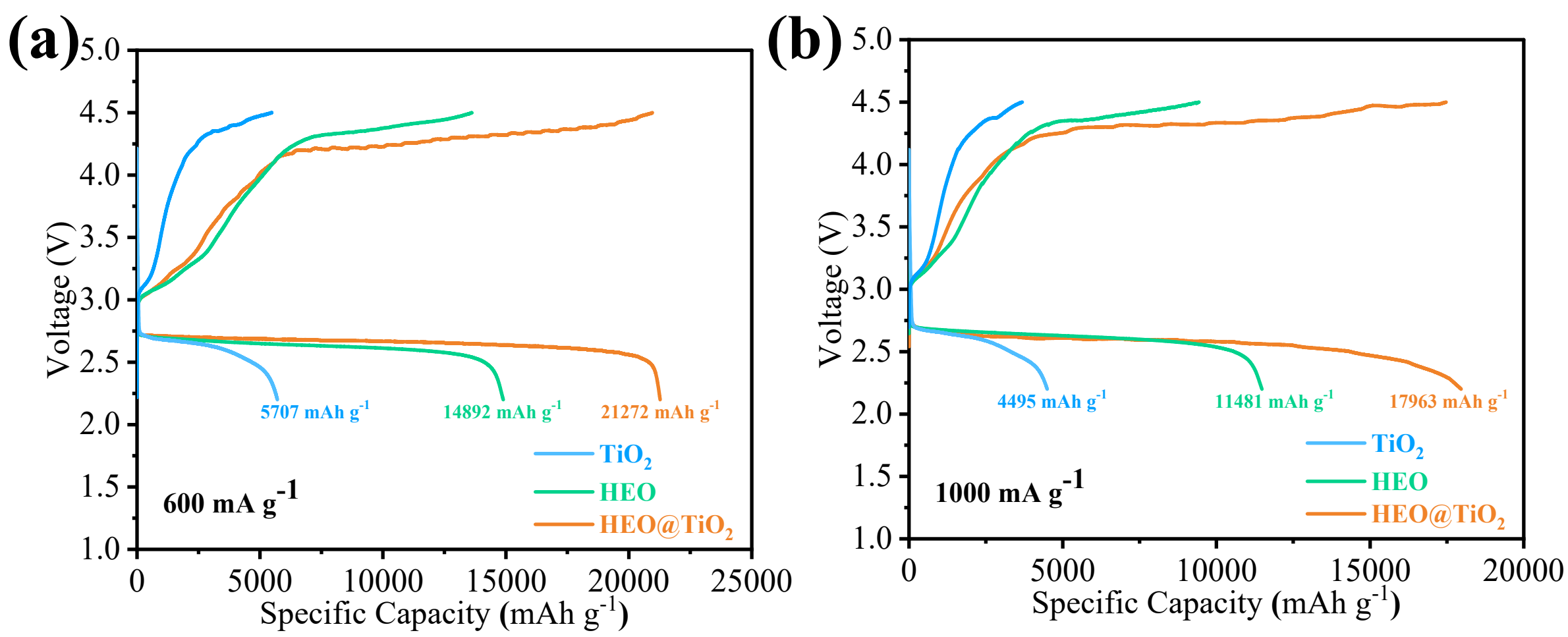


**Fig. S12.** Comparison of the first full charge-discharge curves for batteries assembled with different cathode materials at (a) 600 $mAg^{-1}$ and (b) 1000 $mAg^{-1}$ current densities.

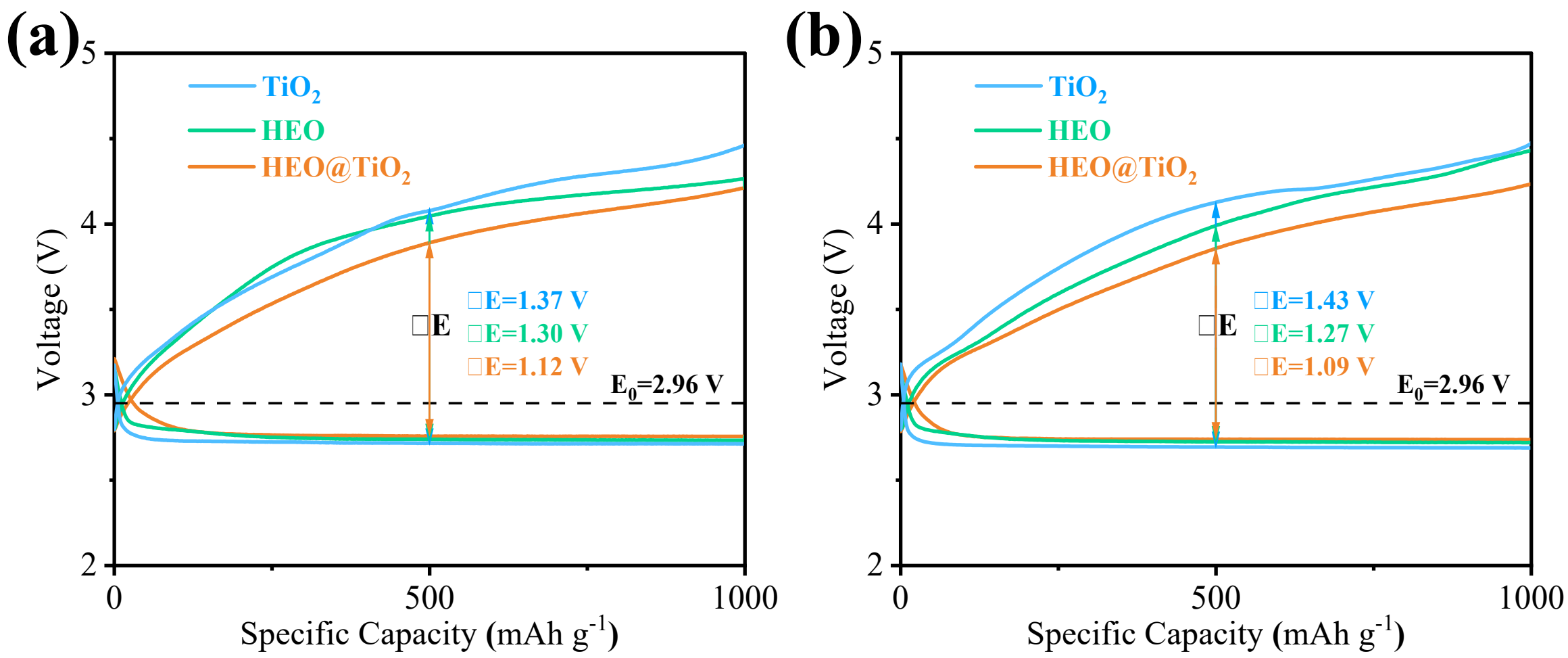


**Fig. S13.** Comparison of the first cycle overpotentials for LOBs assembled with different cathode materials at (a) 600 $mAg^{-1}$ and (b) 1000 $mAg^{-1}$ current densities, with a capacity limit of 1000 mAh $g^{-1}$.

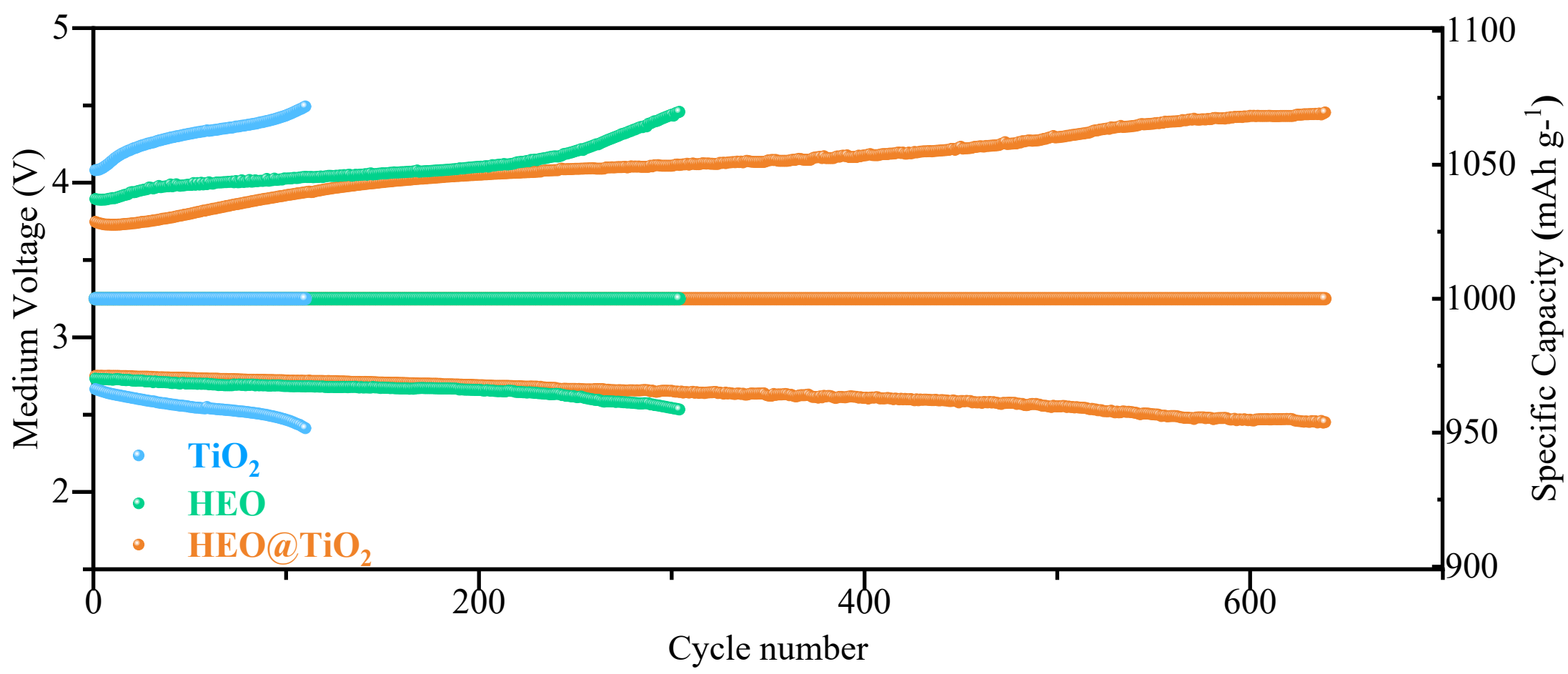


**Fig. S14.** Comparison of cycling performance for LOBs assembled with different cathode materials at a current density of 1000 $mAg^{-1}$and a capacity limit of 1000 mAh $g^{-1}$.

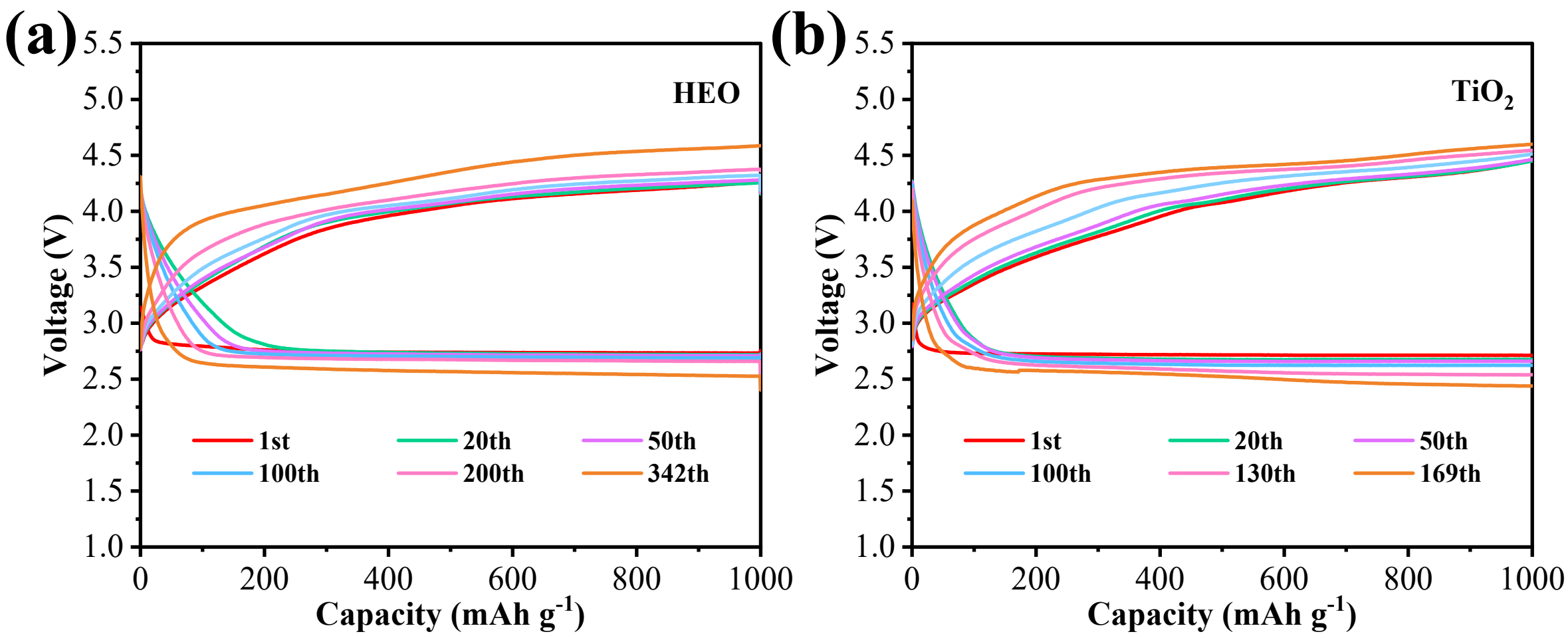


**Fig. S15.** (a) HEO and (b) $TiO_2$ cathode partial charge/discharge curves under a current density of 600 $mAg^{-1}$ and a capacity limit of 1000 mAh $g^{-1}$.

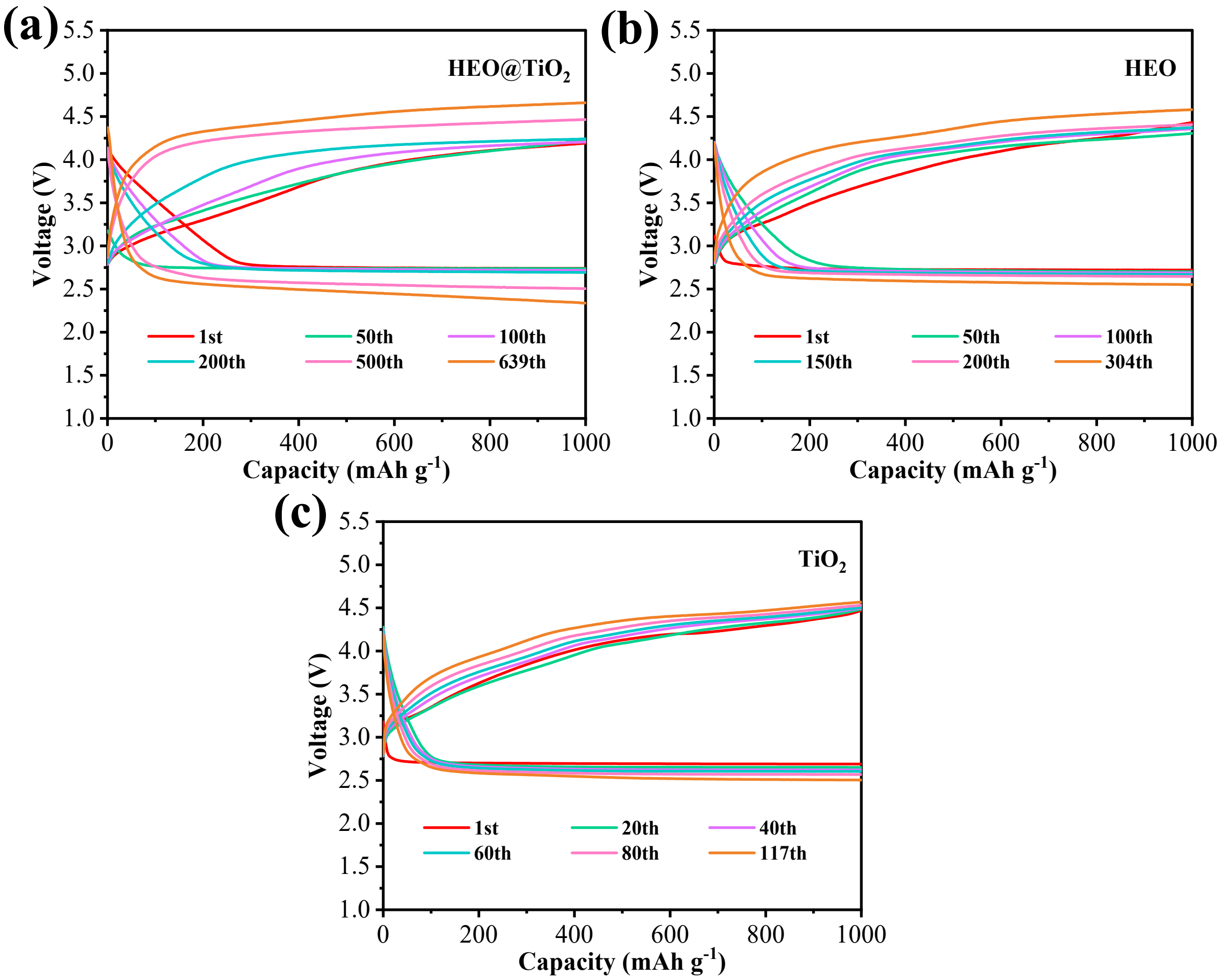


**Fig. S16**. Partial charge/discharge curves for LOBs assembled with different cathode materials at a current density of 1000 $mAg^{-1}$ and a capacity limitof1000 mAh $g^{-1}$.

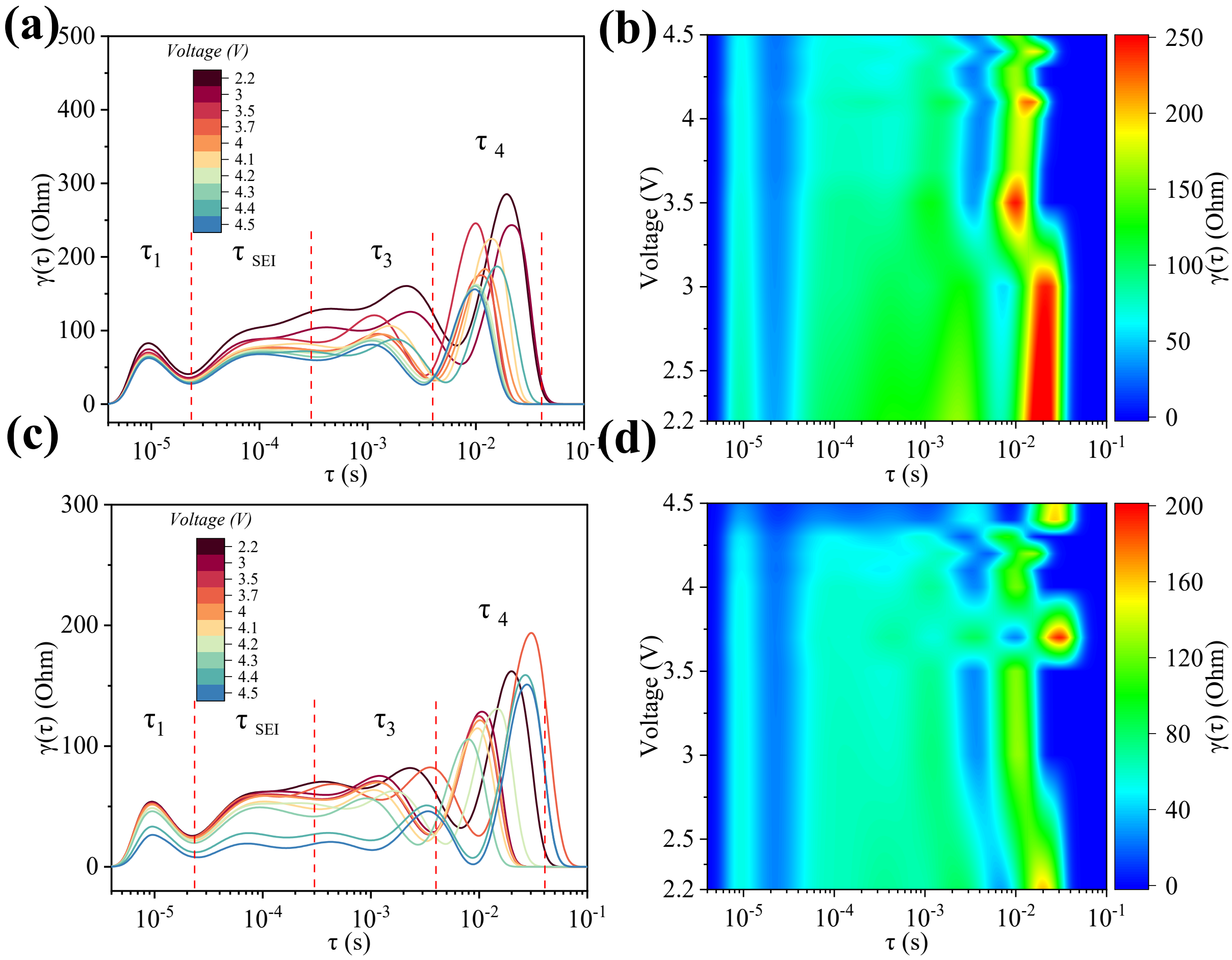


**Fig. S17.** (a-b) DRT spectra and corresponding contour plots of in-situ electrochemical impedance transformation for (a-b) $TiO_2$ and (c-d) HEO electrodes.

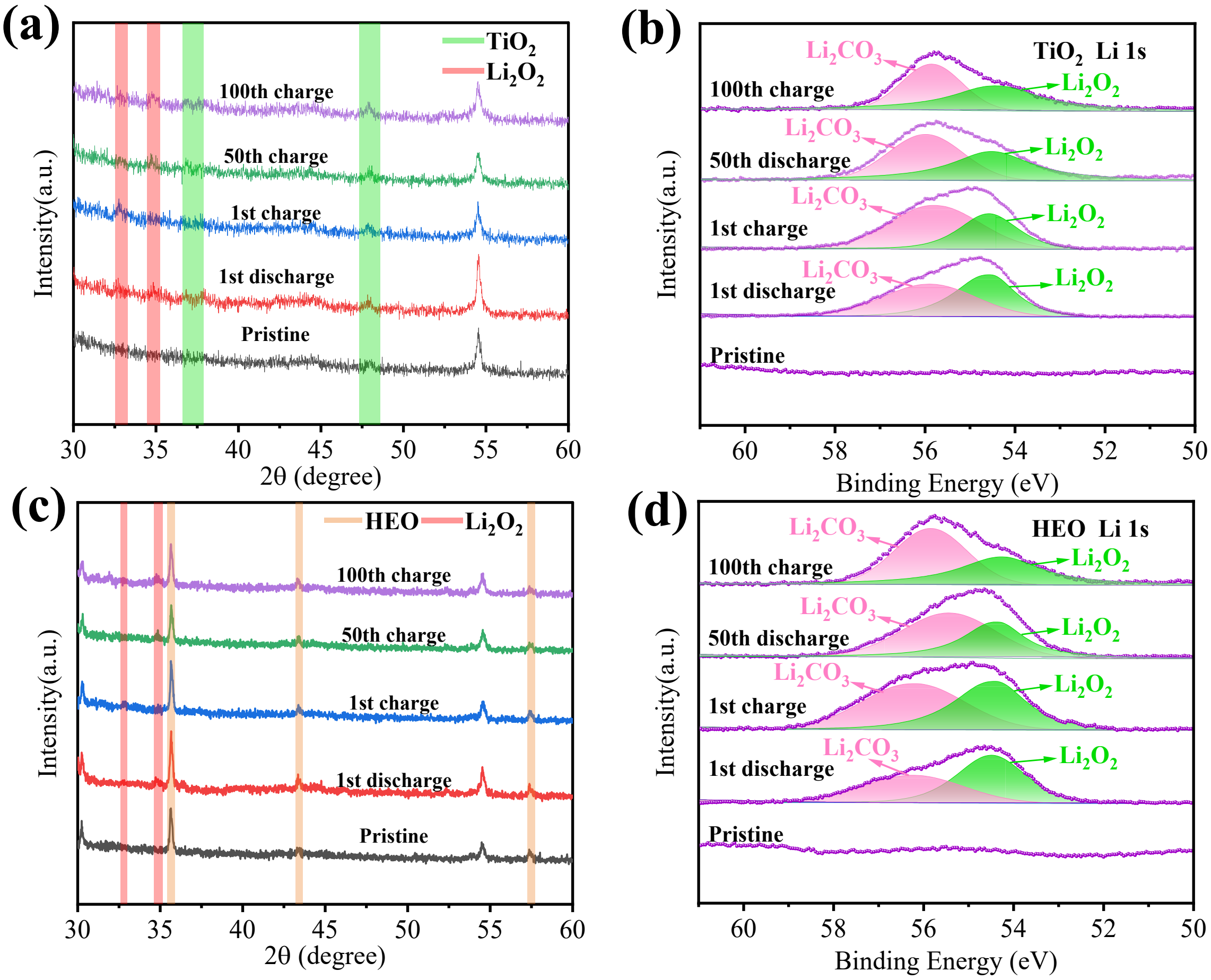


**Fig. S18.** (a-b) XRD and corresponding XPS spectra of (a-b) $TiO_2$ and (c-d) HEO electrodes at different stages of charge/discharge.

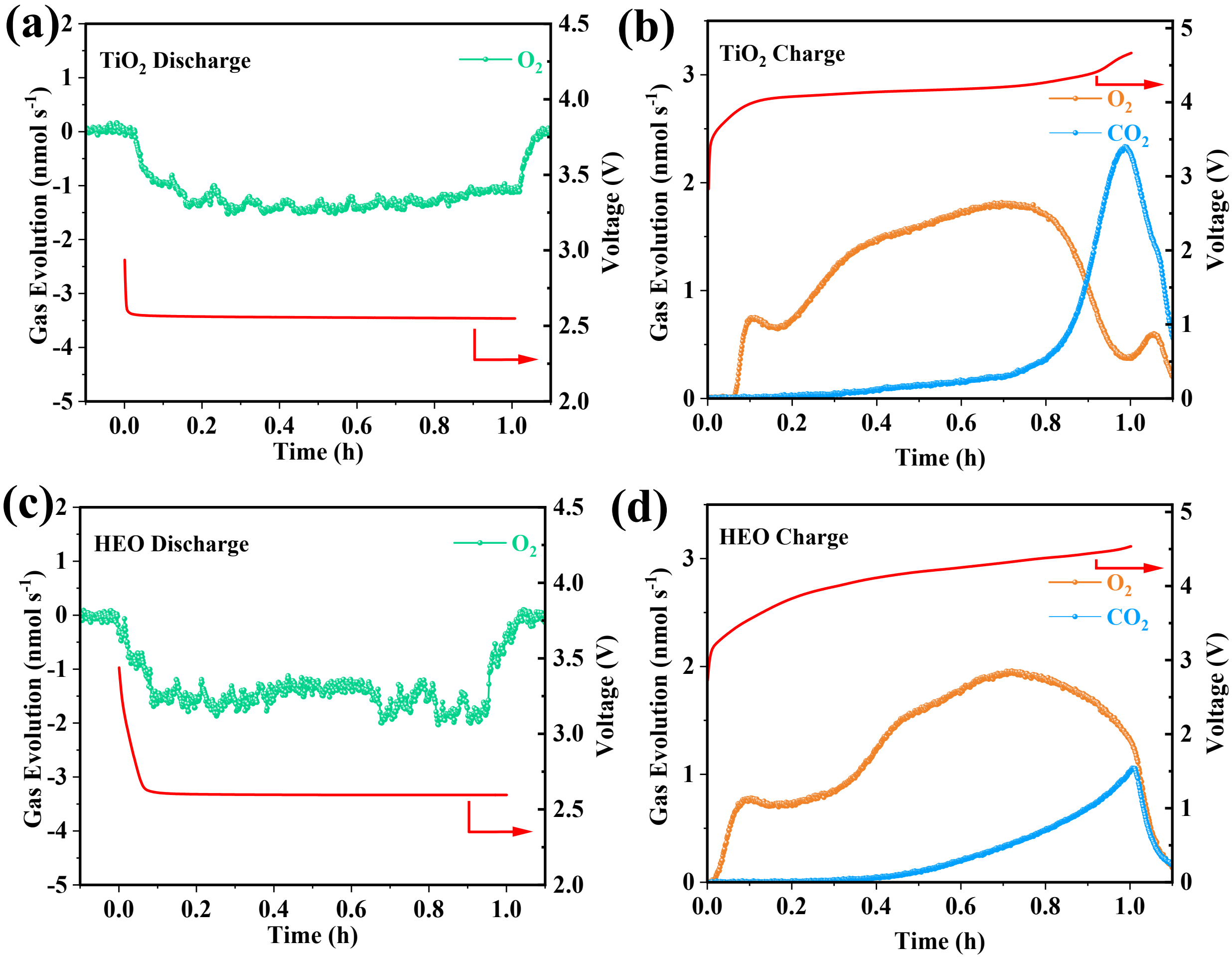


**Fig. S19.** (a-b) Discharge and recharge DEMS spectra for (a-b) $TiO_2$ and (c-d) HEO electrodes.

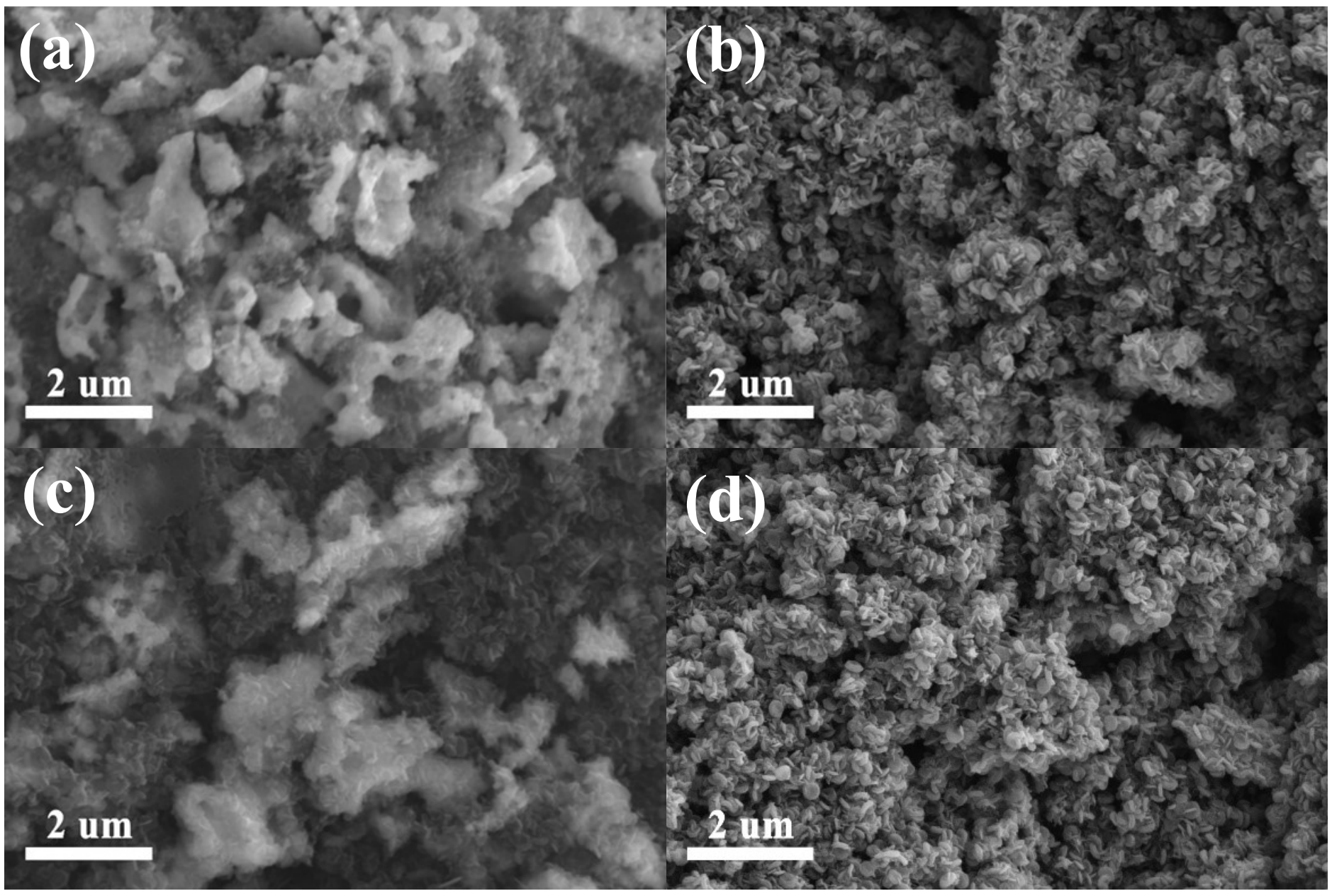


**Fig. S20.** SEM images of HEO electrodes at (a) initial, (b) fully discharged, (c) recharged, and (d) after 100 cycles.

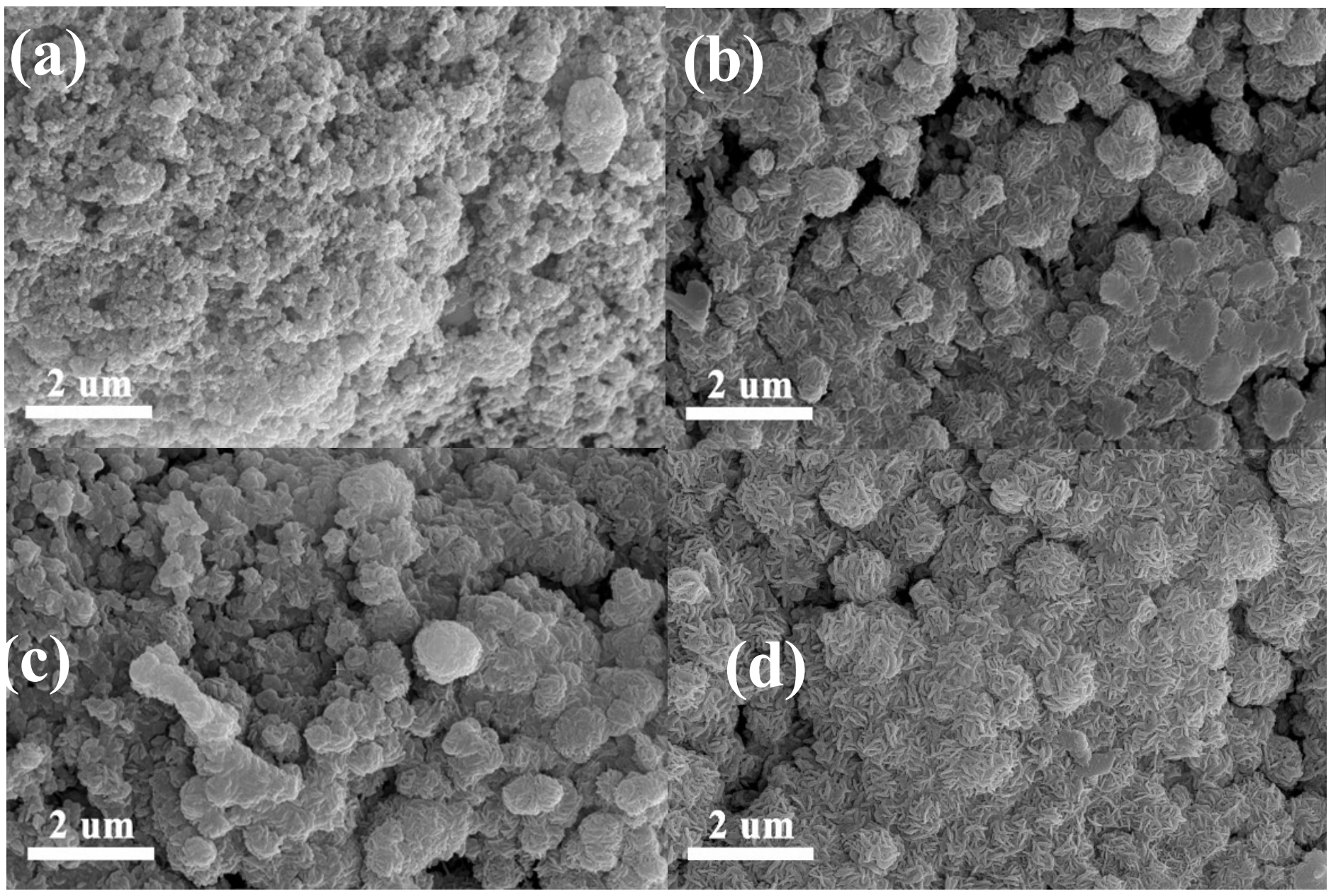


**Fig. S21.** SEM images of $TiO_2$ electrodes at (a) initial, (b) fully discharged, (c) recharged, and (d) after 100 cycles.

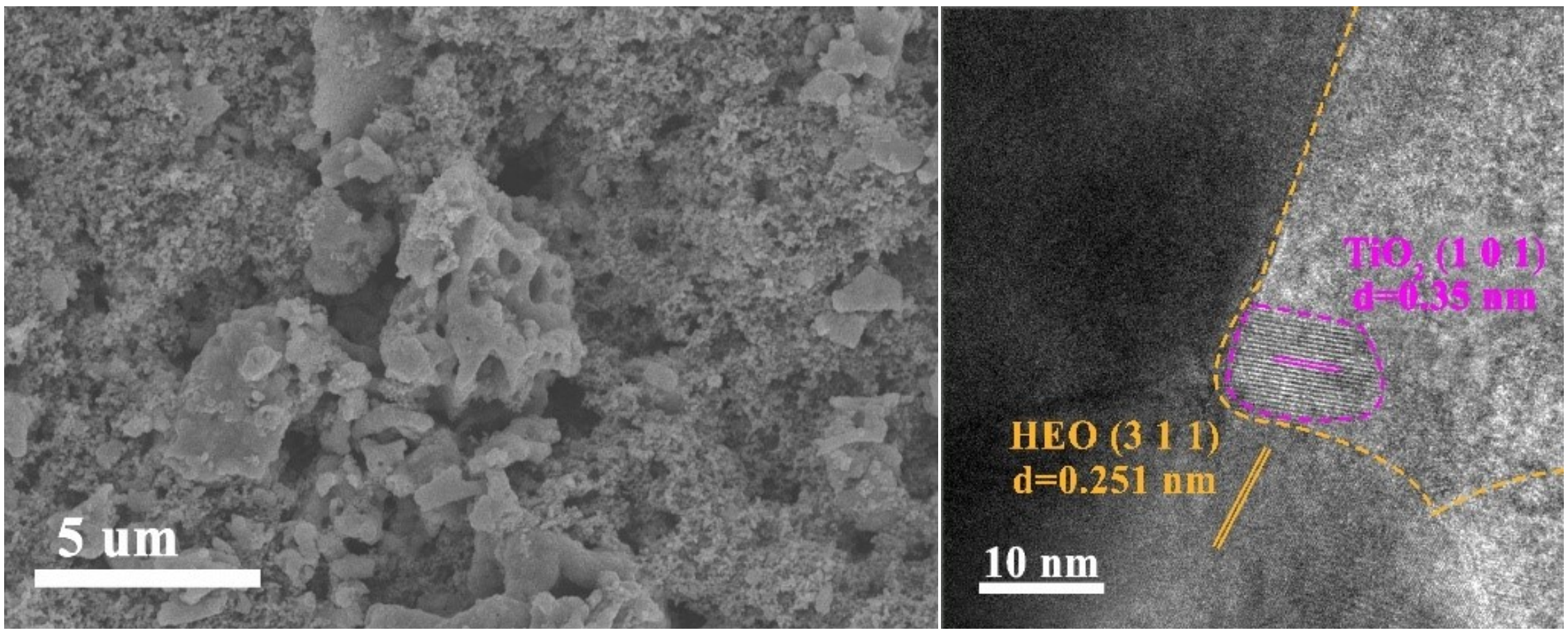


**Fig. S22.** (a) SEM and (b) TEM images of HEO@$TiO_2$ electrodes after 500 cycles.

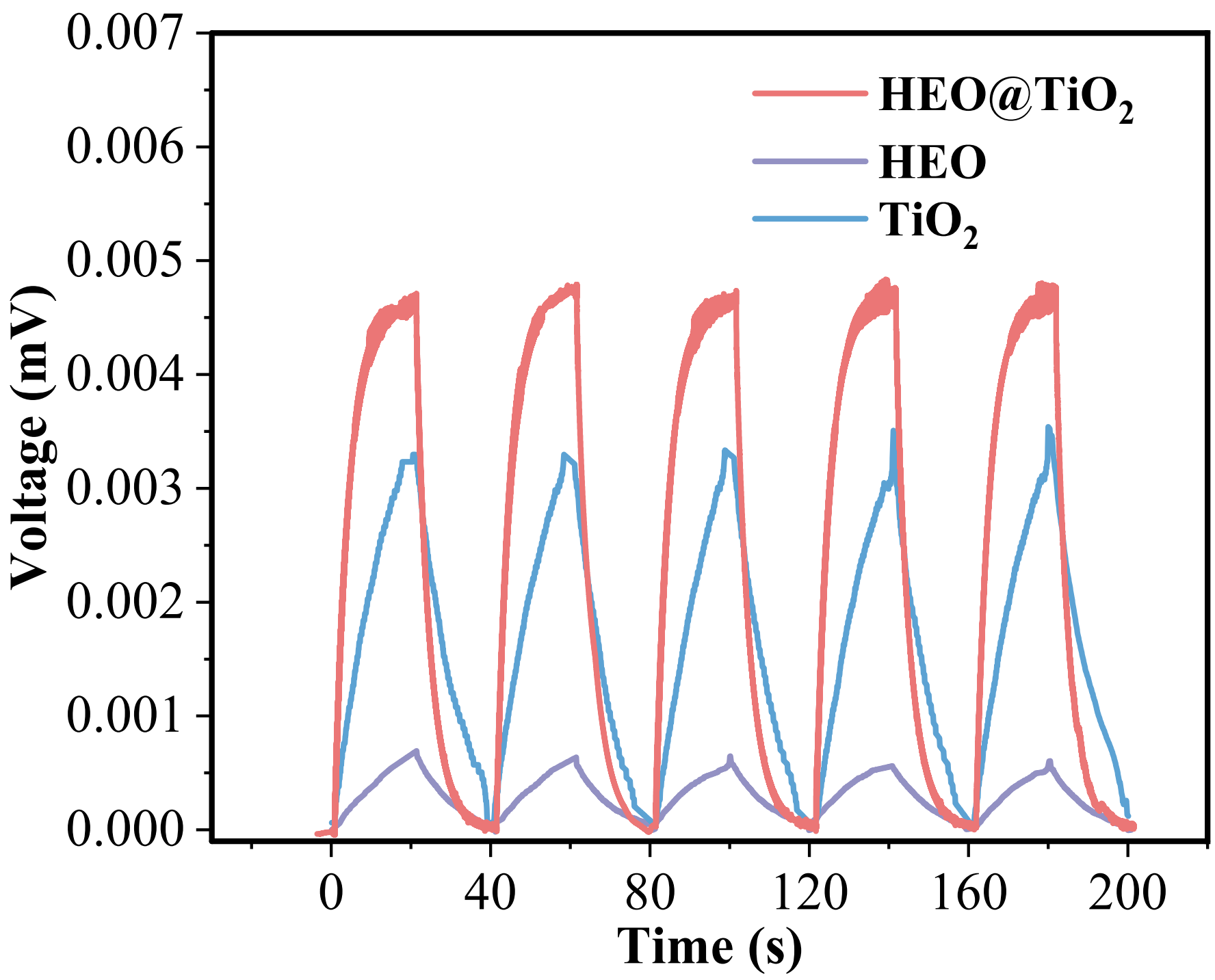


**Fig. S23.** Transient photovoltage of $TiO_2$ 、HEO and HEO@$TiO_2$

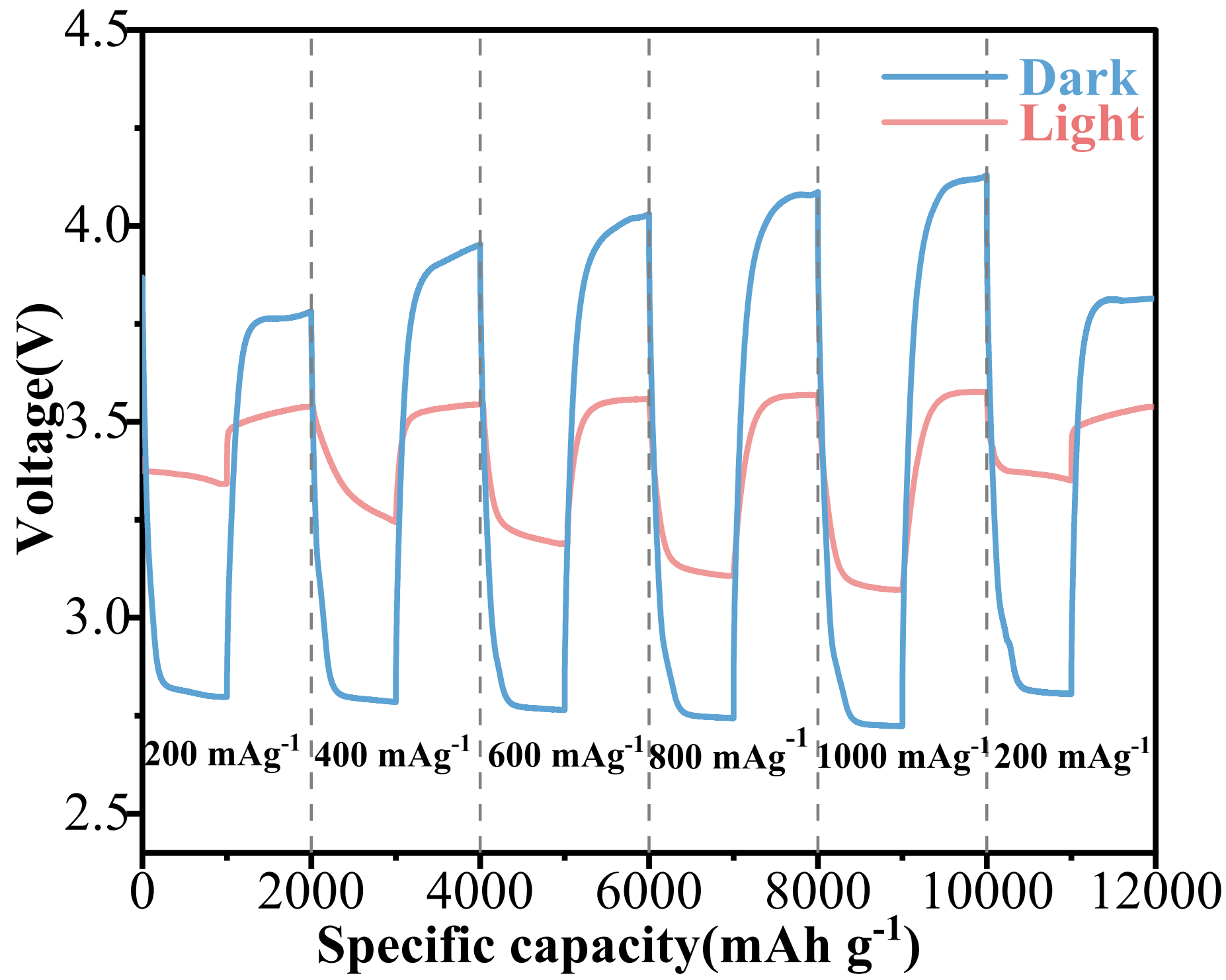


**Fig. S24.** Rate capability under illumination for HEO@$TiO_2$.

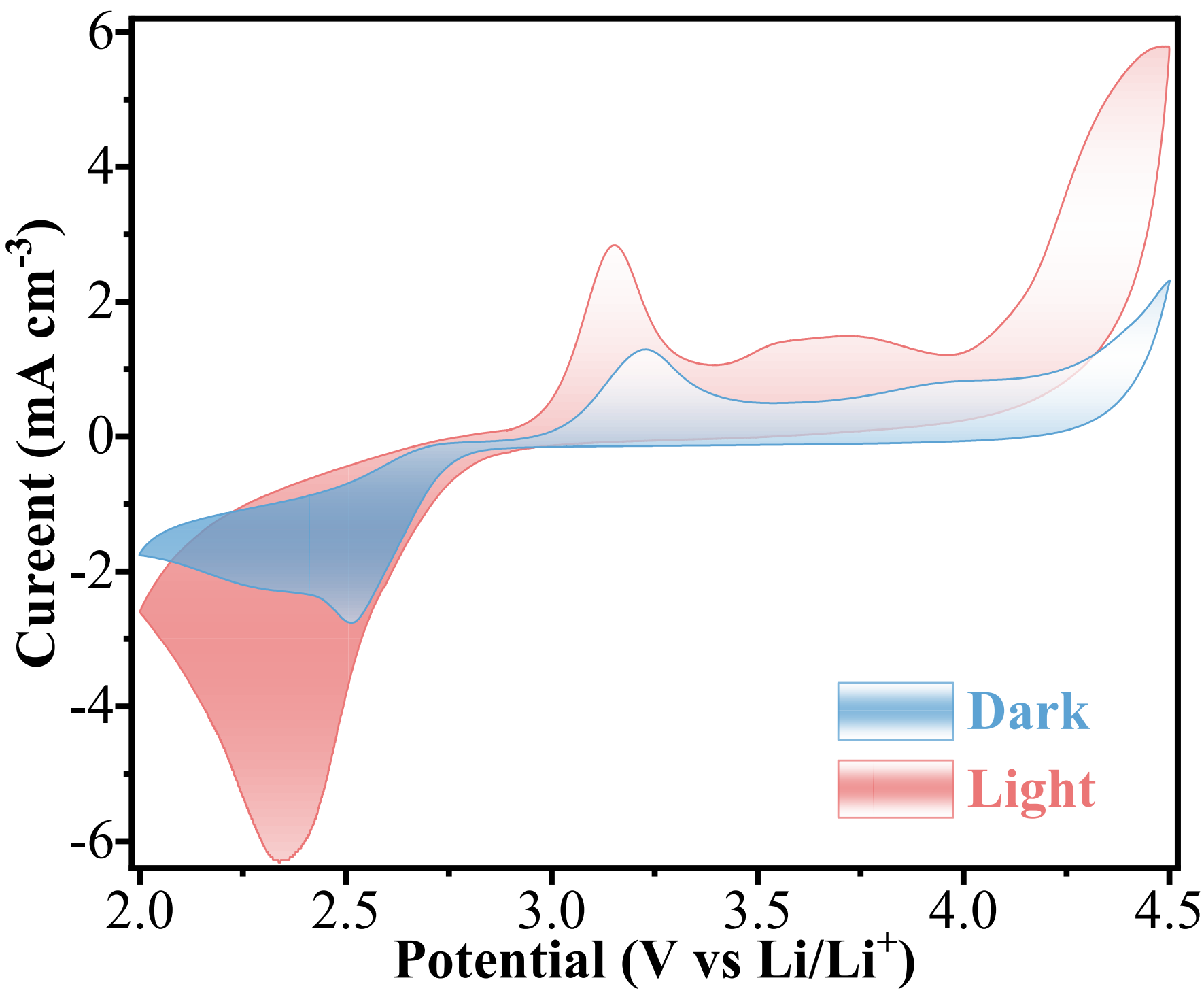


**Fig. S25.** Comparison of CV curves under light and dark conditions.

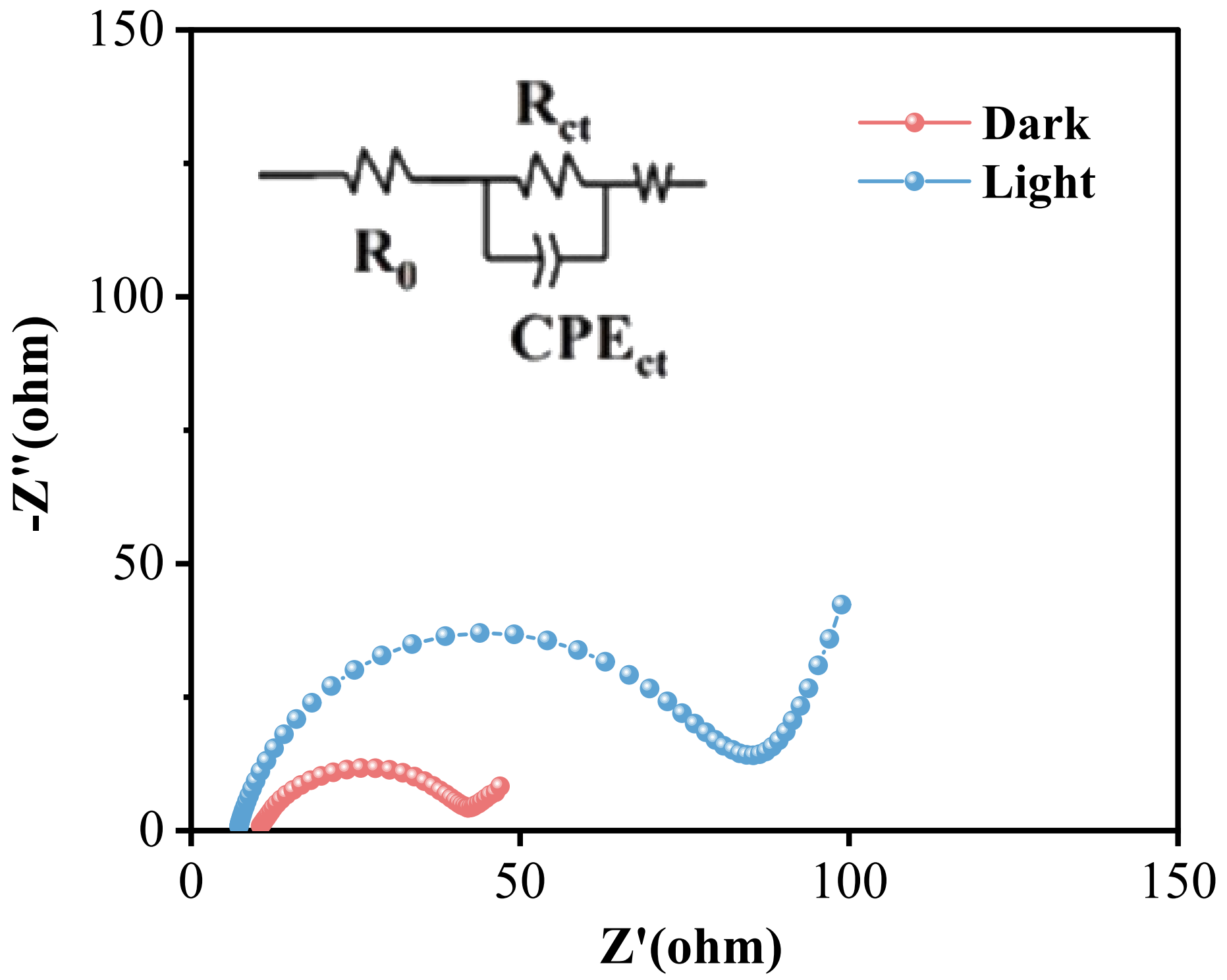


**Fig. S26.** EIS comparison of HEO@$TiO_2$ electrodes under dark/light conditions.

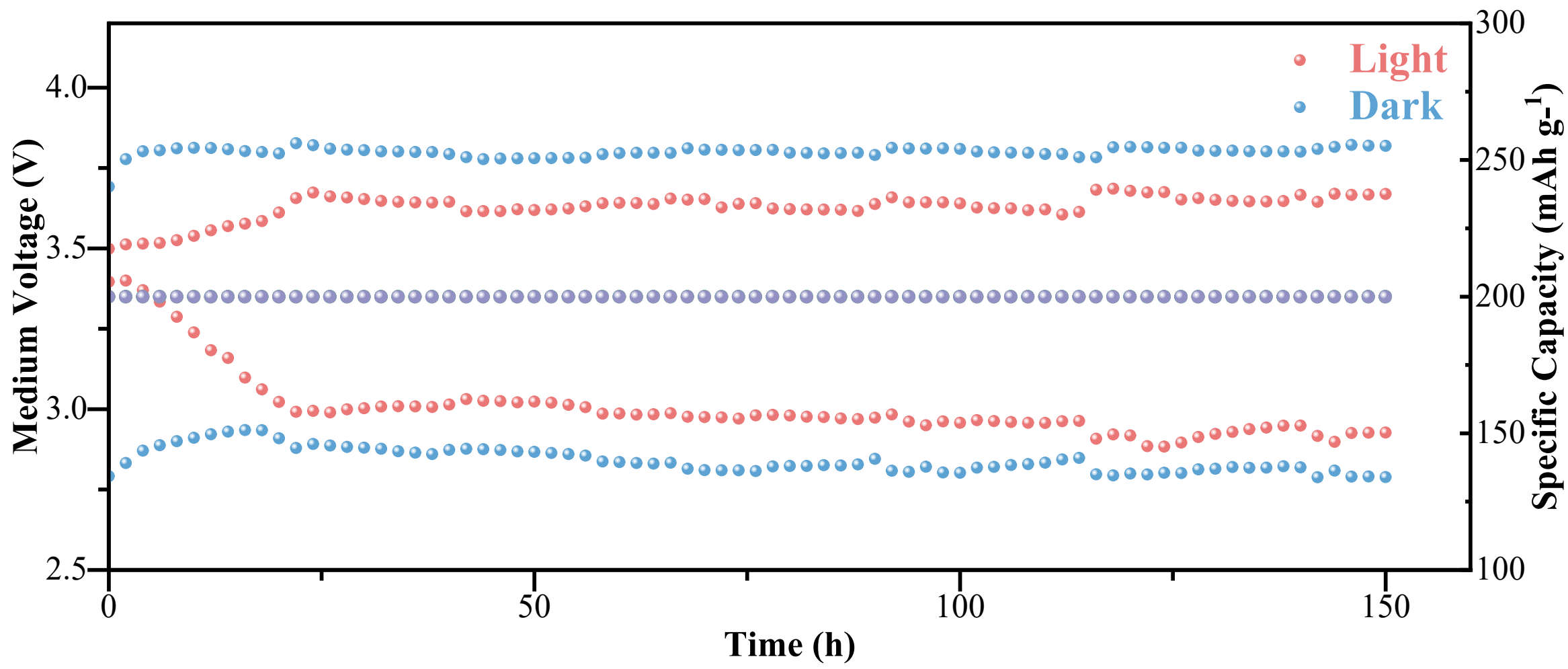


**Fig. S27.** Cycling stability at 200 mA $g^{-1}$ with a limited specific capacity of 200 mAh $g^{-1}$.

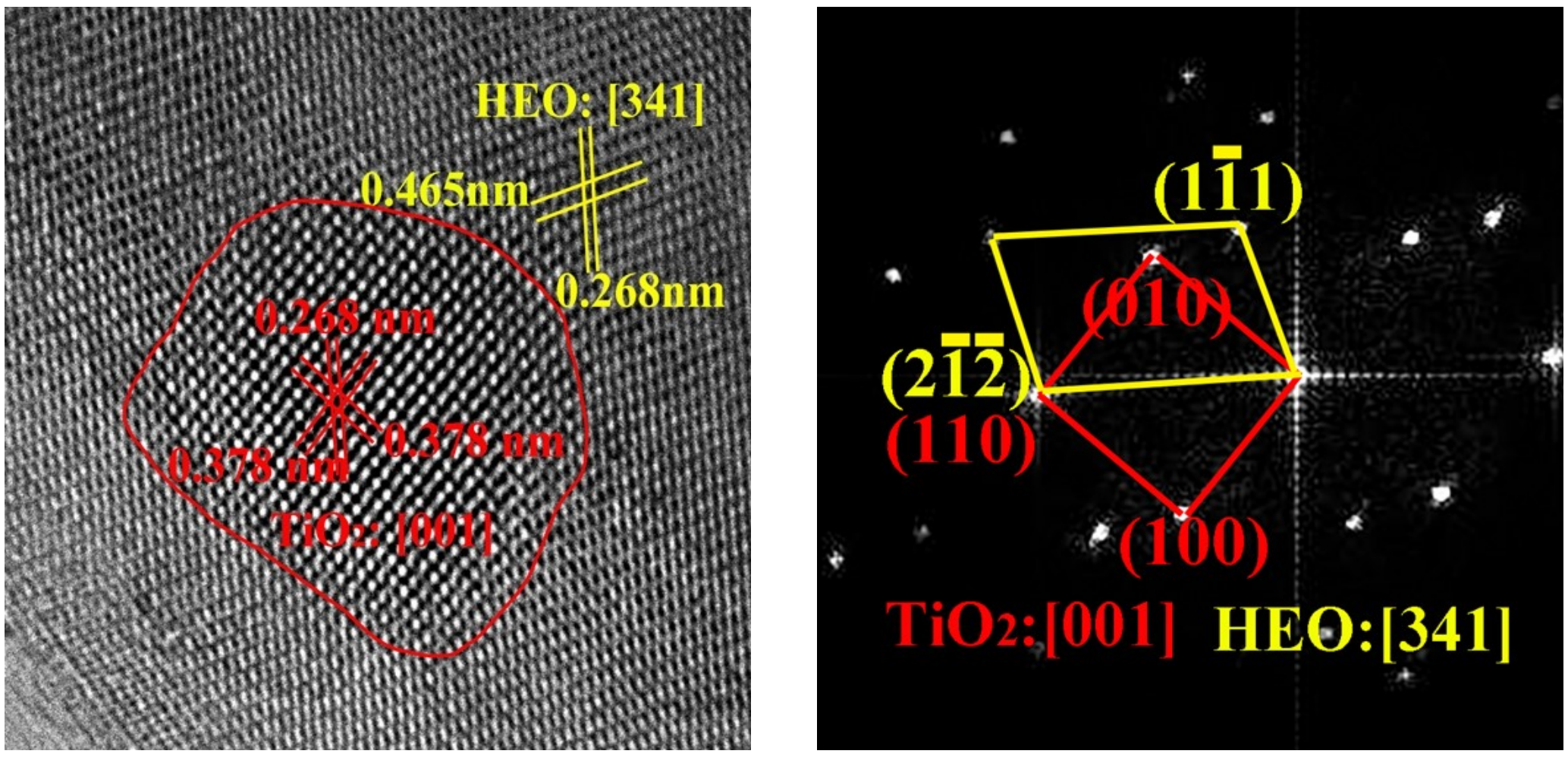


**Fig. S28.** HRTEM image and FFT pattern of the HEO@$TiO_2$ electrode in the fully charged state.

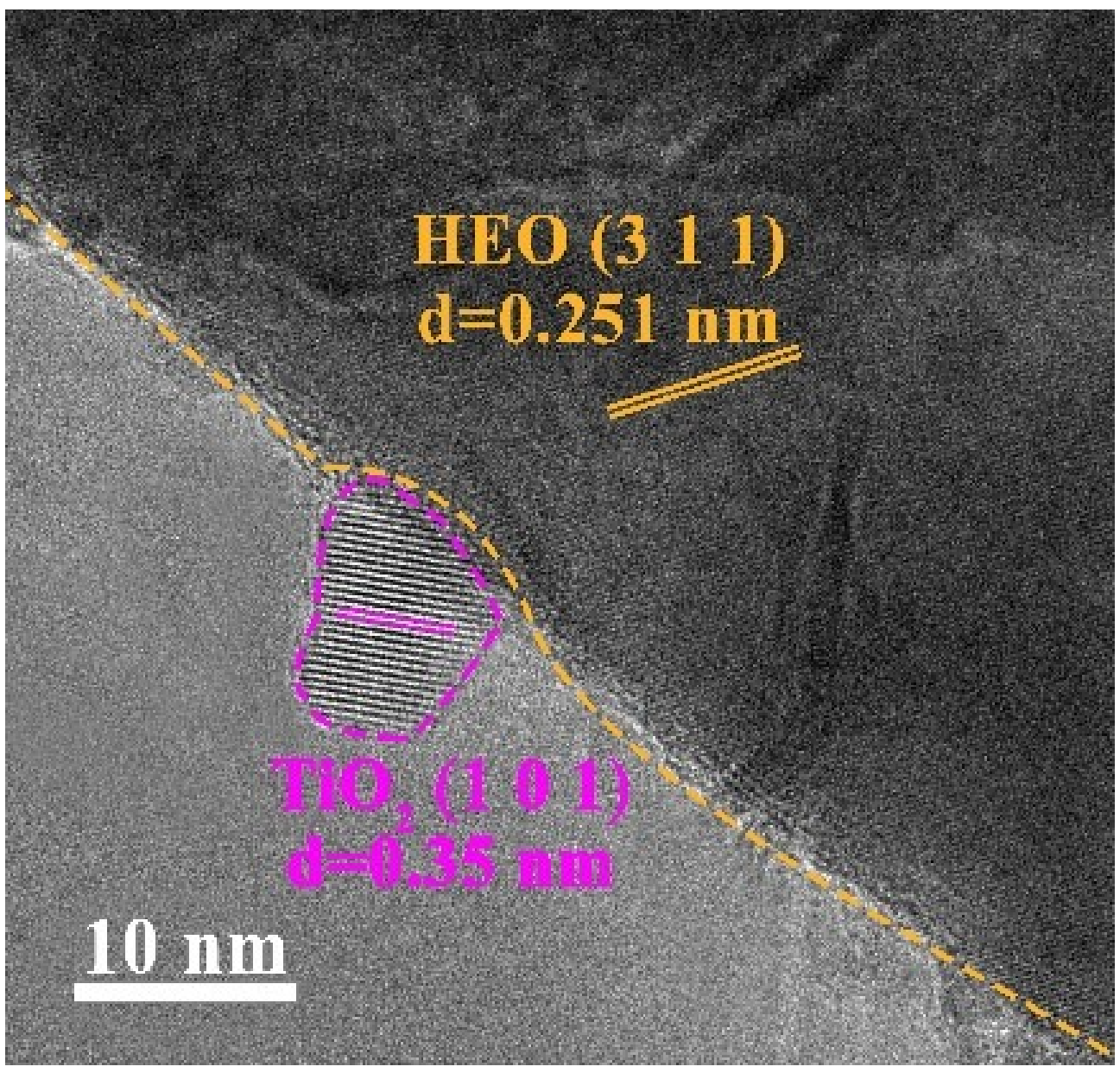


**Fig. S29.** TEM images of HEO@$TiO_2$ electrodes after 150 h of cycling under light illumination.

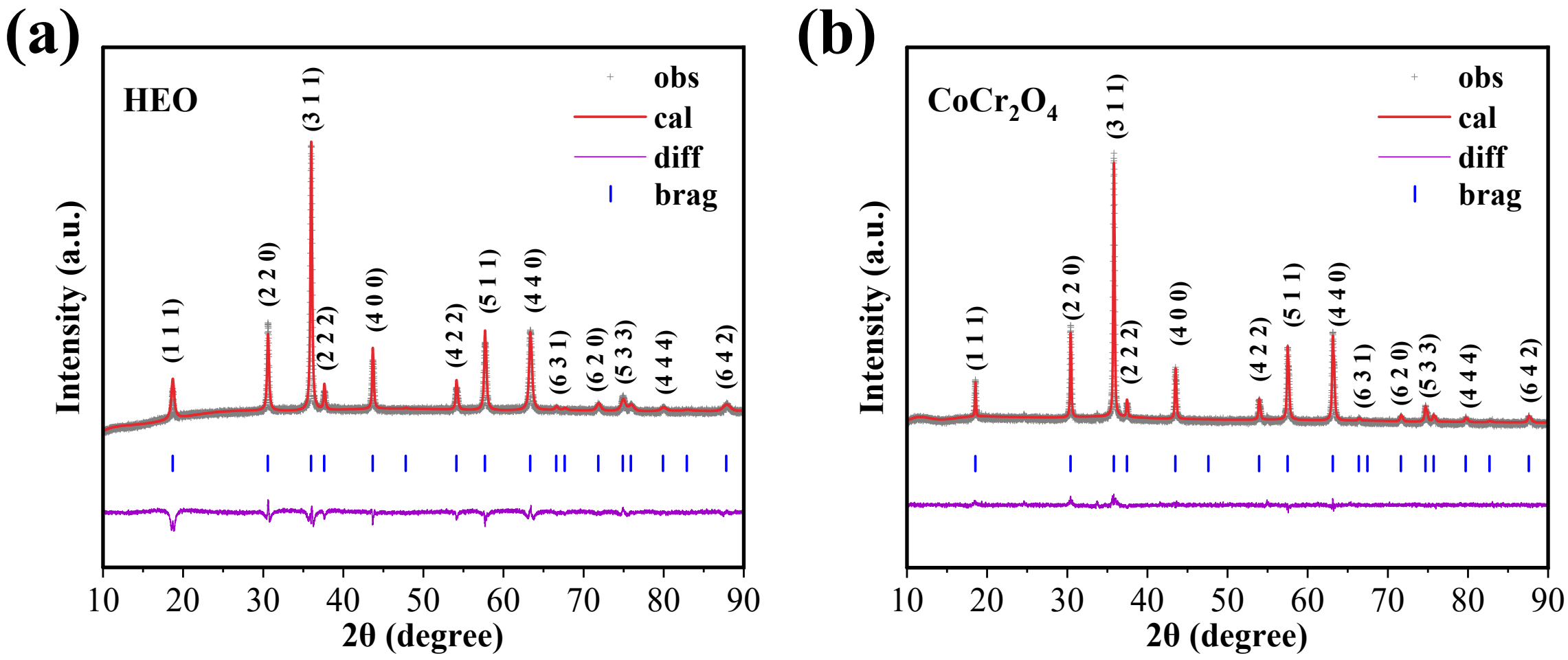


**Fig. S30.** XRD patterns of (a) HEO and (b) $CoCr_2O_4$ with Rietveld fitting. The experimental data are displayed by +, the refined pattern is displayed as a red line superimposed on the experimental data, the blue vertical line indicates the position of the Bragg peak of the spinel core structure, and the difference is displayed in Purple.

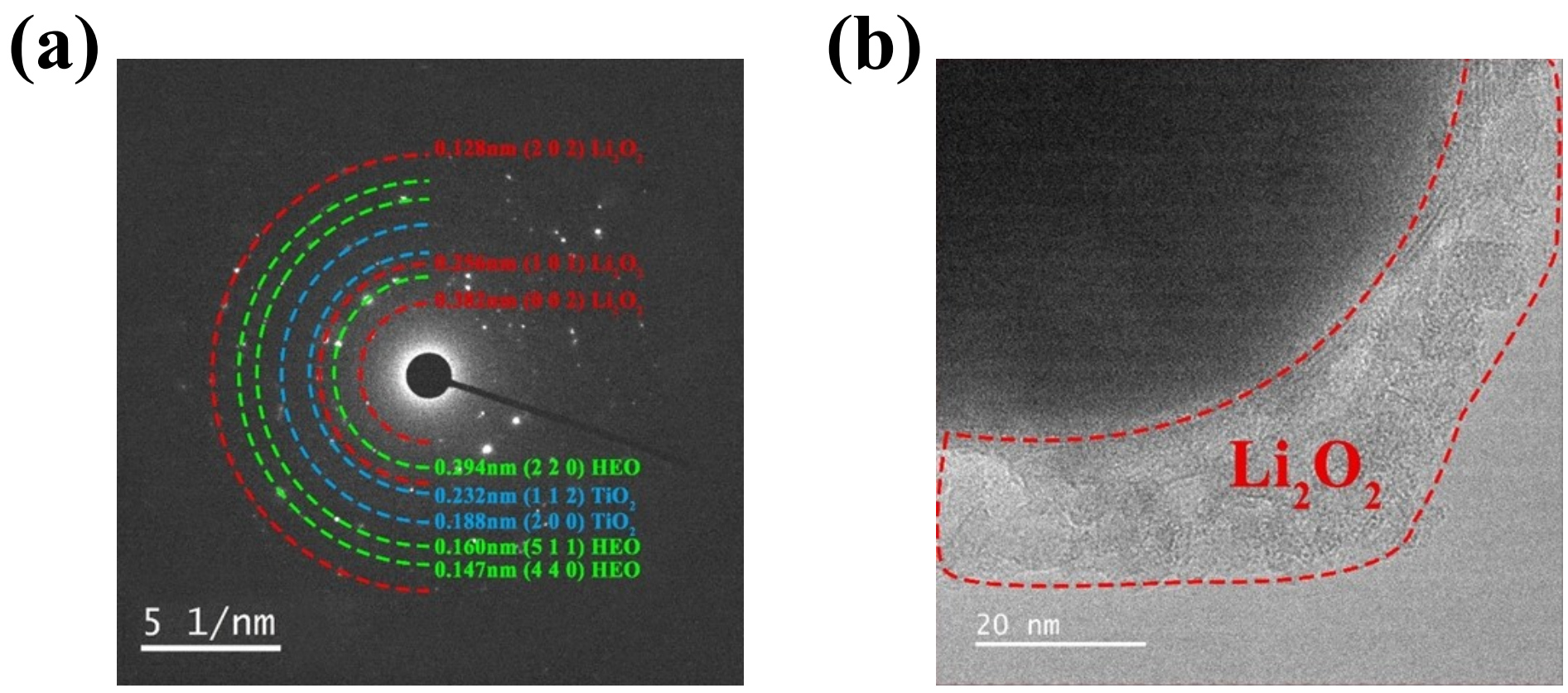


**Fig. S31.** (a) SAED diffraction pattern and (b) HRTEM image of the discharged $HEO@TiO_2$ electrode.

## 3. Table

**Table S1.** Contents of element in HEO and HEO@$TiO_2$ from SEM.

| Element | HEO (At%) | HEO@$TiO_2$ (At%) |
|---|---|---|
| Fe | 7.22 | 8.91 |
| Co | 7.70 | 8.78 |
| Ni | 6.97 | 9.66 |
| Mn | 7.17 | 9.12 |
| Zn | 6.93 | 8.62 |
| Cr | 7.13 | 9.11 |
| O | 56.88 | 45.08 |
| Ti | --- | 0.72 |

**Table S2.** The element contents in HEO and HEO@$TiO_2$ by ICP.

| Element | HEO (mg/L) | HEO@$TiO_2$ ( mg/L ) |
|---|---|---|
| Fe | 4.41 | 4.19 |
| Co | 5.15 | 4.94 |
| Ni | 4.27 | 3.95 |
| Mn | 4.83 | 4.66 |
| Zn | 4.35 | 3.84 |
| Cr | 5.05 | 4.72 |
| Ti | --- | 1.29 |

**Table S3.** Comparison of cycle number of HEO@$TiO_2$ with reported electrocatalysts at different current densities and cutoff capacities.

| Catalyst | Current density (mA $g^{-1}$) | Special capacity (1000 mA h $g^{-1}$) | Cycle number | Reference |
|---|---|---|---|---|
| **HEO@$TiO_2$** | **600** | **1000** | **867** | **This work** |
| **HEO@$TiO_2$** | **1000** | **1000** | **639** | **This work** |
| P-$NiCo_2O_4$ | 200 | 1000 | 70 | [1] |
| NCP@NCO/Ni | 800 | 500 | 409 | [2] |
| $NiRu_3$─N/rGO | 200 | 500 | 220 | [3] |
| $ZnMoO_4$ | 500 | 600 | 580 | [4] |
| HEA@Pt-PtSAs | 1000 | 1000 | 470 | [5] |
| $PtFe_c$/NC | 200 | 1000 | 208 | [6] |
| Ir@$CeO_2$-O | 500 | 500 | 221 | [7] |
| $Ti_{0.87}O_2$/MXene | 200 | 600 | 425 | [8] |
| LaSr(5TM)$O_3$ | 200 | 500 | 226 | [9] |
| MnTe/MnO | 200 | 1000 | 350 | [10] |

**Table S4.** EXAFS fitting parameters at the Fe K-edge for various samples. (a) CN, coordination number; (b) R, distance between absorber and backscatter atoms; (c) σ2, Debye-Waller factor to account for both thermal and structural disorders; (d) ΔE(0), inner potential correction; R factor indicates the goodness of the fit. S02 was fixed to 0.7 for Fe. A reasonable range of EXAFS fitting parameters: 0.700<S02<1.000; CN>0; σ2Å2＞0; |ΔE0|<15 eV; R factor<0.02.

| Sample | Shell | $CN^a$ | $R$(Å)$^b$ | $\sigma^2$ (Å$^2$)$^c$ | $\Delta E_0$(eV)$^d$ | R factor |
|---|---|---|---|---|---|---|
| Fe Foil | Fe-$Fe_1$ | 8* | 2.47 | 0.0049 | 6.38 | 0.0049 |
| | Fe-$Fe_2$ | 6* | 2.85 | 0.0057 | | |
| $Fe_2O_3$ | Fe-O | 2* | 1.91 | 0.0051 | -8.01 | 0.0121 |
| | Fe-O | 4* | 2.02 | 0.0069 | | |
| $Fe_3O_4$ | Fe-O | 4* | 1.94 | 0.0067 | -6.51 | 0.0162 |
| HEO | Fe-O | 3.5 | 1.92 | 0.0049 | 1.21 | 0.010 |
| | Fe-O-Fe/$Ti_1$ | 3.5 | 2.96 | 0.0098 | | |
| | Fe-O-Fe/$Ti_2$ | 9.0 | 3.47 | 0.0091 | | |
| HEO@$TiO_2$ | Fe-O | 4.2 | 1.95 | 0.0056 | -3.05 | 0.0076 |
| | Fe-O-Fe/$Ti_1$ | 4.1 | 2.95 | 0.0084 | | |
| | Fe-O-Fe/$Ti_2$ | 9.2 | 3.46 | 0.0099 | | |

| Sample | Lattice parameters/Å | | | Volume/ $Å^3$ | Reliability factors | | Microstrain |
|---|---|---|---|---|---|---|---|
| | a | b | c | | $R_p$/% | $R_{wp}$/% | |
| $CoCr_2O_4$ | 8.14518 | 8.14518 | 8.14518 | 531.8 | 0.98% | 1.37% | 512.7 |
| HEO | 8.37156 | 8.37156 | 8.37156 | 579.4 | 1.86% | 2.25% | 989.5 |

**Table S5.** Rietveld refinement data for $CoCr_2O_4$ and HEO.

**References**


[1] S. Shoukat, Y. Dou, M. Gao, D. Wan, M. Shoaib, S. Iqbal, A. Tariq, I. Rafique, A. Said, S. Li, I. Haider, J. Wang, *Chem. Eng. J.*, 2025, **513**, 162908.

[2] S. Karunarathne, G. E. Perez, W. P. S. L. Wijesinghe, F. Orange, Y. Y. Kannangara, C. R. Ratwani, C. Sandaruwan, A. Mija, A. R. Kamali, A. M. Abdelkader, *J. Mater. Chem. A*, 2025, **13**, 11344-11357.

[3] T. Bai, J. Wang, W. Song, H. Zhang, Z. Han, F. Ji, C. Zhang, W. Zhai, X. Wang, Y. Wu, D. Gao, J. Lu, L. Ci, D. Li, *Adv. Funct. Mater.*, 2025, **35**, 2424014.

[4] M. Yu, G. Yi, X. Zhang, X. Ding, Z. Zou, H. Ma, Z. Zhao, Y. Fan, *Adv. Compos. Hybrid Mater.*, 2025, **8**, 141.

[5] K. Cheng, L. C. J. Smulders, L. I. Van der wal, J. Oenema, J. D. Meeldijk, N. L. Visser, G. Sunley, T. Roberts, Z. Xu, E. Doskocil, H. Yoshida, Y. Zheng, J. Zečević, P. E. De jongh, K. P. De jong, *Scienc*, 2022, **377**, 204.

[6] Y. Rao, J. Yang, J. Tian, W. Ning, S. Guo, H. Zhou, *Angew. Chem. Int. Ed.*, 2025, **64**, e202418893.

[7] Y. Zhao, Z. Wang, S. Guan, Y. Gao, S. Gong, G. Han, P. Gao, S. Lou, Y. Zhu, X. Li, *Chem. Eng. J.*, 2024, **500**, 156972.

[8] D. Zhang, G. Zhang, R. Liu, R. Yang, X. Li, X. Zhang, H. Yu, P. Zhang, B.-W. Li, H. Hou, Z. Guo, F. Dang, *Adv. Funct. Mater.*, 2025, **35**, 2414679.

[9] J. Hao, Z. Zhuang, K. Cao, G. Gao, C. Wang, F. Lai, S. Lu, P. Ma, W. Dong, T. Liu, M. Du, H. Zhu, *Nat. Commun.*, 2022, **13**, 2662.

[10] S. Yin, D. Yan, Y. Yan, S. Liu, Q. Lu, X. Guan, Q. Zhang, Y. Xing, P. Yang, S. Zhang, *Small*, 2024, **20**, 2406525.
